# Target-Centric Multiplexed Screening of an Herbal Extract Identifies a Novel Dual A_2A_/A_2B_ Receptor Antagonist for Cancer Immunotherapy

**DOI:** 10.1021/acscentsci.5c01843

**Published:** 2026-03-13

**Authors:** Hongyue Liu, Xinyu Yang, Jingyi Xu, Yuefei Wang, Zichen Zhao, Ke Quan, Aoqi Gao, Yang Wang, Jingbo Wu, Fei Li, Zhaoyu Zhang, Yuanyuan Ma, Yuan Weng, Ying Chen, Liping Sun, Gaojie Song, Yibing Shan, Xin Chai, Bingjie Zhang, Weiqiang Lu, Wenqing Shui

**Affiliations:** † iHuman Institute, 387433 ShanghaiTech University, Shanghai 201210, China; ‡ School of Life Science and Technology, 387433 ShanghaiTech University, Shanghai 201210, China; § University of Chinese Academy of Sciences, Beijing 100049, China; ∥ Shanghai Key Laboratory of Regulatory Biology, Institute of Biomedical Sciences and School of Life Sciences, 198170 East China Normal University, Shanghai 200241, China; ⊥ 58301 Tianjin University of Traditional Chinese Medicine, Tianjin 301617, China; # Department of Urology and Department of Pathology, 71529 The Fifth People’s Hospital of Shanghai, Fudan University, Shanghai 201100, China; ∇ Hainan Academy of Medical Sciences, Hainan Medical University, Haikou 571199, Hainan, China; ○ Engineering Research Center of Tropical Medicine Innovation and Transformation of Ministry of Education, Hainan Provincial Key Laboratory for Research and Development of Tropical Herbs and Haikou Key Laboratory of Li Nationality Medicine, School of Pharmacy, Hainan Medical University, Haikou 571199, China; ◆ State Key Laboratory of Innovative lmmunotherapy, School of Pharmaceutical Sciences, Shanghai Jiao Tong University, Shanghai 200240, China

## Abstract

Medicinal herbs contain natural products (NPs) possessing rich scaffolds valuable for drug discovery, particularly in oncology. While most NP-derived cancer therapeutics directly kill tumor cells, emerging opportunities lie in modulating antitumor immunity. However, target-annotated NPs for cancer immunotherapy remain scarce. Herein we established a multiplexed platform combining virtual screening, affinity selection-mass spectrometry, and metabolomics profiling to identify bioactive NPs targeting the adenosine 2A receptor (A_2A_R), a master regulator of tumor immunosuppression. Screening the crude extract of a medicinal herb and isolating the active constituent resulted in the discovery of a novel dual antagonist for A_2A_R/A_2B_R with preferential activity on A_2A_R. This compound, ER-15, adopts a unique binding mode as revealed by structural modeling, MD simulations, mutagenesis, and SAR analysis. Functionally, ER-15 reversed adenosine-mediated immunosuppression and augmented the immune checkpoint inhibitor therapy in both the animal model and patient-derived tumor organoids, supporting its therapeutic potential in anti-PD-1-resistant tumors. Therefore, our strategy is expected to overcome traditional NP discovery bottlenecks, enabling efficient identification of target-annotated novel leads for drug development.

## Graphical abstract

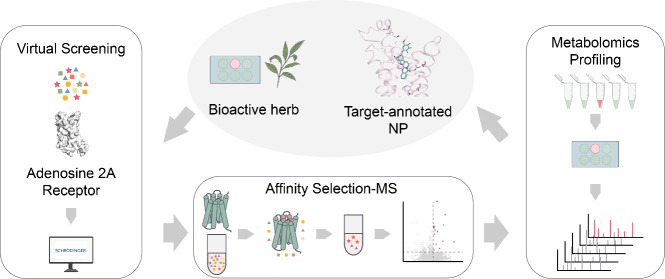

## Introduction

Natural products (NPs), derived from medicinal herbs and microorganisms, represent a rich reservoir for drug discovery due to their wide chemical space and evolutionarily optimized properties.^1–3^ NPs and their structural analogues have historically made a major contribution to pharmacotherapy, especially for cancer and infectious diseases.^2–4^ In regard to cancer therapeutics, whereas the majority of NP-based drugs directly induce death or inhibit proliferation of cancer cells, an important new opportunity lies in the potential of NPs to trigger or enhance host immune responses against cancer cells.^3^ However, identification and characterization of NPs with bioactivity specific for cancer immunotherapy is still in its infancy. Though a few natural products capable of modulating tumor immunosuppression have been reported, most of them were discovered from cell-based phenotypic screens where molecular targets and structural mechanisms remain elusive, impeding further drug development.^5–9^

Identification of new NPs with desired bioactivity and characterization of their molecular mechanism of action represent long-standing challenges for NP-based drug discovery. Classical activity-guided compound isolation starts with biological screening of crude extracts, followed by laborious fractionation of the bioactive extract in order to isolate the active constituents. This process of NP isolation and identification is largely hindered by the difficulty in capturing trace-amount bioactive compounds and elucidating their molecular target(s) underlying the cellular phenotype.^10^ On the other hand, target-based bioassay screening and virtual screening have been applied to NP compound libraries, which is typically restrained by the limited diversity or insufficient quantities of available purified NP compounds.^11–14^ Notably, affinity-selection mass spectrometry (AS-MS) allows for directly capturing and detecting chemical ligands from complex matrices toward kinases or viral proteases.^15–19^ Particularly, our lab previously adapted the AS-MS technique to screening herbal extracts, leading to the discovery of new-scaffold agonists for a G protein-coupled receptor (GPCR), serotonin receptor 2C, with distinct pharmacological properties.^20^

In this study, to accelerate the identification of new NPs with defined molecular mechanisms suitable for cancer immunotherapy, we selected another GPCR protein, the adenosine A_2A_ receptor (A_2A_R), as our primary target. A_2A_R is activated by the endogenous ligand, adenosine, which is a metabolic byproduct enriched in the tumor microenvironment.^21^ Activation of the adenosine-A_2A_R pathway not only mediates immunosuppression directly but also triggers increased expression of other immune checkpoints, including PD-1, TIM-3 and CTLA-4.^22^ Therefore, targeting this master pathway has been recognized as a promising approach for those with tumors resistant to existing therapies, including immune checkpoint inhibitors.^22,23^ Moreover, considering the coexpression and functional interplay of A_2A_R and the adenosine A_2B_ receptor (A_2B_R), dual blockade of A_2A_R and A_2B_R signaling represents another attractive strategy that is manifested by two synthetic dual antagonists that alleviate immunosuppression and enter clinical trials for multiple cancer types.^22–28^

Herein, we built a multiplexed screening platform by integrating virtual screening, AS-MS, and metabolomics profiling to enable confident identification of a new-scaffold A_2A_R antagonist (ER-15) from the complex matrix of a Traditional Chinese Medicinal (TCM) herb. Guided by AS-MS, we then isolated this low-abundance component from the herbal extract for bioactivity characterization. ER-15 exhibits dual antagonism at A_2A_R and A_2B_R with a stronger potency at A_2A_R. Interestingly, structural modeling, MD simulations, and experimental validation indicate that this novel antagonist adopts a binding mode distinct from known A_2A_R ligands. Furthermore, we demonstrated the high efficacy of ER-15 in alleviating immunosuppression and augmenting the anti-PD-1 immunotherapy using T cell activation assays and tumor models.

## Results

### Bioactivity Screening of Herbal Extracts

A_2A_R is known to induce Gs-coupled cAMP signaling upon activation.^29^ To select natural herbs that may contain A_2A_R antagonists, we first measured the inhibitory activity of 30 crude extracts from 13 TCM herbs on A_2A_R-mediated cAMP accumulation in HEK293 cells. To maximize the structural diversity of NPs in these extracts, we prepared crude extraction materials using several solvents of varying polarity to obtain the aqueous phase, ethyl acetate phase, and total alkaloids (AP/EP/TA) separately from specific herbs (Figure S1A and Table S1). In this single-dose bioactivity screening assay, the total alkaloid extracts from three herbs, i.e., *Evodia rutaecarpa* (ER), *Aristolochia debilis* (AD), *Coptis chinensis* (CC), and the ethyl acetate phase extract of *Lycium barbarum* (LB) achieved more than 50% inhibition of A_2A_R-induced cAMP signaling, with the ER and AD extracts showing the strongest activity of 97% and 91% inhibition, respectively (Figure S1A).

The total alkaloid extracts of ER and AD were then assessed by dose–response cAMP accumulation assays to determine their antagonistic activities at three receptors within the adenosine subfamily. The ER extract exhibited dose-dependent antagonistic activity at both A_2A_R and A_2B_R, with IC_50_ of 35.4 μM and 304 μM, respectively (assuming the average molecular weight of chemical ingredients in the extract is 500 Da), yet showed no activity at A_1_R (Figures 1A-C). The AD extract also exhibited dual antagonistic activity at A_2A_R and A_2B_R with weaker potency than the ER extract, and no activity at at A_1_R (Figures 1A-C). The other two extracts from herbs CC and LB, failed to display dose-dependent activity at either A_2A_R or A_2B_R (Figures S1B, C).

**Figure 1:**
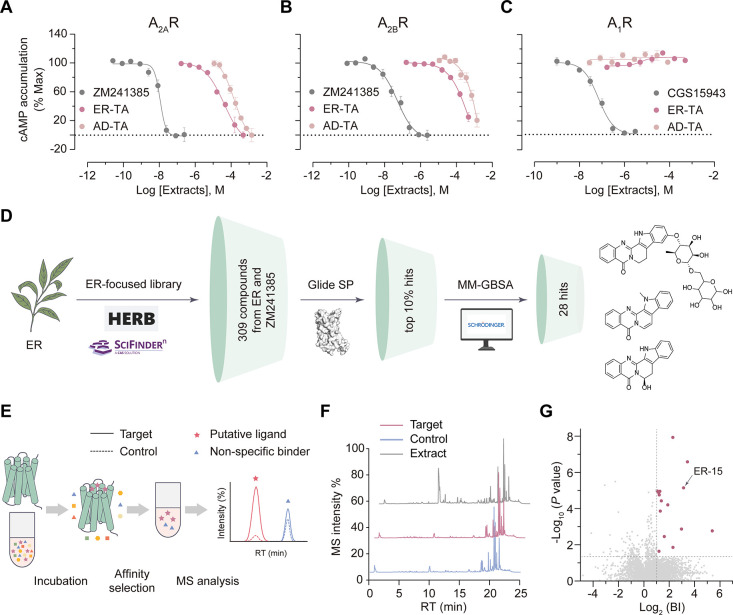
**Identification of putative A**_**2A**_ **R ligands from a bioactive TCM herb.** (**A-C**) Dose-response curves of cAMP accumulation assays in A_2A_R (**A**), A_2B_R (**B**) or A_1_R (**C**)-transfected HEK293 cells treated by the total alkaloid extracts from ER or AD (ER-TA, AD-TA). The estimated concentrations of herbal extracts were calculated from weight (assuming an average molecular weight of 500 Da). ZM241358, reference antagonist for A_2A_R and A_2B_R; CGS15943, reference antagonist for A_1_R. Data are shown as means ± SEM from three independent experiments. (**D**) Virtual screening workflow for identifying A_2A_R ligands from an ER-focused compound library. Chemical structures of top 3 docking hits are shown. (**E**) Scheme for AS-MS screening of GPCR ligands. Figure was created with BioGDP.com. (**F**) Representative total ion chromatograms (TICs) of the ER extract, target (A_2A_R-associated), and control (control protein-associated) samples. (**G**) AS-MS screening of the ER extract resulted in 15 hit compounds (red dots). The binding index (BI) is defined as the ratio of the compound’s MS intensity detected in the target *vs* control from four indepedent experiments. Positive binders are those with an average BI > 2 (*P* < 0.01, *n* = 4).

Taken together, we speculated that both ER and AD extracts contain antagonistic compound(s) with subtype selectivity toward A_2A_R and A_2B_R or either receptor. Given the higher inhibitory potency of the ER extract, it was selected for subsequent bioactive NP screening.

### Multiplexed Screening for Bioactive NP Identification

To identify the constituent(s) in the ER extract responsible for the dual A_2A_R/A_2B_R antagonism, we integrated three orthogonal screening approaches that are either binding-based or bioactivity-based. First, we conducted a small-scale virtual screen by generating a focused library composed of 308 compounds previously identified from ER that are reported in literature^30,31^ or curated in natural product databases including HERB^32^ and TCMHD.^33^ These compounds were subjected to molecular docking using Glide, targeting the orthosteric binding site in the inactive A_2A_R crystal structure^34^ (PDB ID: 4EIY). The top 10% of good scoring compounds were retained and further processed by Prime MM-GBSA for binding free energy calculation. To verify the scoring accuracy and sampling efficiency of docking, we redocked two known A_2A_R antagonists of different potency, a synthetic compound ZM241385 (IC_50_ of 10 nM) and a natural product theophylline (IC_50_ of 3 μM), which gave rise to reasonable docking scores and binding energy (Figure S2). Based on these two reference ligands analysis, we filtered the previously retained compounds and visually inspected their binding poses to select 28 candidates as the docking hits (Figure 1D and Table S2).

At the meantime, we performed AS-MS to directly capture potential binders to the A_2A_R protein from the ER extract. In this experiment, a thermostabilized A_2A_R variant^34^ was purified, immobilized on the magnetic beads, and incubated with the ER extract. Ligands that are specifically bound to A_2A_R were subsequently dissociated from the receptor–ligand complex and analyzed by ultrahigh-performance liquid chromatography coupled to high-resolution mass spectrometry (UPLC-HRMS). By measuring the MS peak intensity of each compound associated with the target protein relative to the control, we were able to identify the A_2A_R ligands against nonspecific binders (Figures 1E, F). The validity of our AS-MS approach was confirmed with a reference compound mixture in which six known A_2A_R ligands were distinguished from six unrelated compounds based on an AS-MS-derived binding index (BI) (Figure S3). Applying the same BI threshold, our AS-MS screen of the ER extract toward the A_2A_R protein led to the identification of 15 distinct MS features that were tentatively assigned to 20 ER constituents (Figure 1G and Table S3). Like the docking hits, these AS-MS hits are also considered as putative A_2A_R ligands.

Prefractionation has been shown to reduce the chemical complexity of crude extracts, eliminate interfering substances, and improve hit rates in NP drug discovery.^2,3^ To complement the two binding-based screening approaches, we introduced the third analytics, metabolomics profiling, which allows the identification of bioactivity-associated components in different fractions. We first separated the ER extract into five subfractions (F1–F5) using the same UPLC method as AS-MS and collecting each subfraction at a 5 min interval (Figure 2A). Subfractions F2, F3, and F4 inhibited cAMP accumulation in both A_2A_R- and A_2B_R-transfected cells, with greater potency than the initial extract. In contrast, F1 and F5 showed little to no activity (Figures 2B, C). However, in untransfected cells, F2 and F4 potently suppressed forskolin-stimulated cAMP accumulation at high doses, while F3 had only a slight inhibitory effect (Figure 2D). This suggests constituents in F2 and F4 likely acted through off-target inhibition of adenylate cyclase rather than receptor antagonism. In addition, reanalysis of our AS-MS data revealed that more than half of the MS features (8 out of 15) representing putative A_2A_R ligands were eluted within the time window corresponding to F3 (10–15 min) (Figure 2E). Altogether, we concluded that F3 is the subfraction most likely to enrich antagonizing compounds that are specific for A_2A_R or for both A_2A_R and A_2B_R.

**Figure 2:**
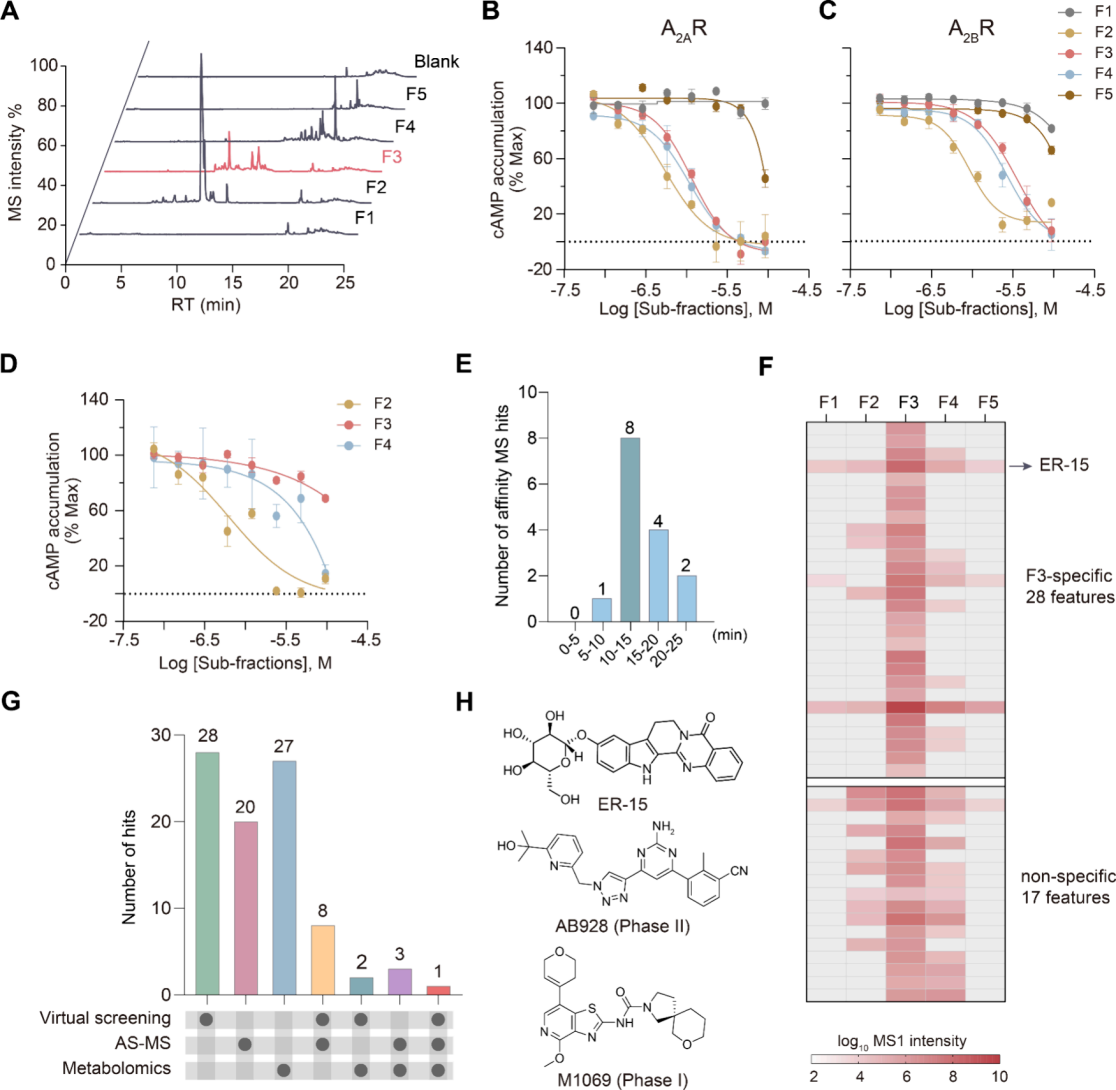
**Identification of the most confident hit ER-15 with the multiplexed screening strategy.** (**A**) TICs of the five ER subfractions and a blank control in the metabolomics experiment. (**B–C**) Dose–response curves of cAMP accumulation assays in A_2A_R- (**B**) or A_2B_R-transfected (**C**) HEK293 cells treated by individual subfractions. (**D**) Dose–response curves of cAMP accumulation assays in forskolin-stimulated HEK293 cells by individual subfractions. Data in (**B-D**) are shown as means ± SEM from three independent experiments. (**E**) The number of AS-MS hits in different retention time (RT) ranges. (**F**) Differential analysis of ER constituents based on MS intensities yielded 28 F3-specific MS features including the ER-15 feature (upper) and 17 nonspecific features (lower). See Methods for metabolomics data analysis. (**G**) Total numbers of screening hits yielded by three different approaches (left three bars) and numbers of intersecting hits coidentified from two or three approaches (right four bars). Intersecting hits from three approaches yielded one hit (red bar), ER-15, coidentified by all. (**H**) The chemical structures of ER-15 and two synthetic dual A_2A_R/A_2B_R antagonists currently in clinical trials.

Next, we performed metabolomics profiling of these five subfractions to identify chemical constituents specifically present in subfraction F3. Among 45 MS features detected in F3 which can be assigned to ER constituents according to their accurate mass and MS/MS spectra, 28 features were exclusively detected or significantly enriched by >100-fold increase of intensity in F3 (Figure 2F). These F3-specific MS features matched to 27 ER constituents are considered metabolomics screening hits (Table S4).

Intersecting hits from three orthogonal screening approaches resulted in 8 compounds coidentified by virtual screen and AS-MS screen which both depend on receptor–ligand binding whereas the bioactivity-correlated metabolomics screening yielded 2 or 3 hits overlapping with either set of binding-based screening hits (Figure 2G). Remarkably, only one compound, ER-15, was coidentified by all three methods (Figure 2G). It is tentatively assigned to rutaecarpine-10-*O-β*-d-glucopyranoside^35^ based on our MS analysis. Compared to synthetic A_2A_R/A_2B_R dual antagonists reported to date,^24–28^ ER-15 possesses a characteristic indoloquinazoline alkaloid skeleton with a glucopyranosyl moiety attached to C-10 position of rutaecarpine (Figure 2H), and it has never been functionally characterized since initial isolation.^35^

### AS-MS-Guided Compound Isolation and Bioactivity Characterization

In our attempt to isolate ER-15 for bioactivity measurement, we started with the total alkaloid extract of ER which was fractionated sequentially using flash column chromatography (FCC) and preparative high-performance liquid chromatography (prep-HPLC) (Figure 3A). During the multiround fractionation, the AS-MS screening data provided critical guidance by specifying the expected retention time (RT) and accurate mass of the compound of interest. By analyzing each subfraction with UPLC-HRMS and matching the compound features with AS-MS data, we were able to obtain a preparative subfraction Fr.H which showed MS features of ER-15 in the expected RT range (Figure 3B).

**Figure 3:**
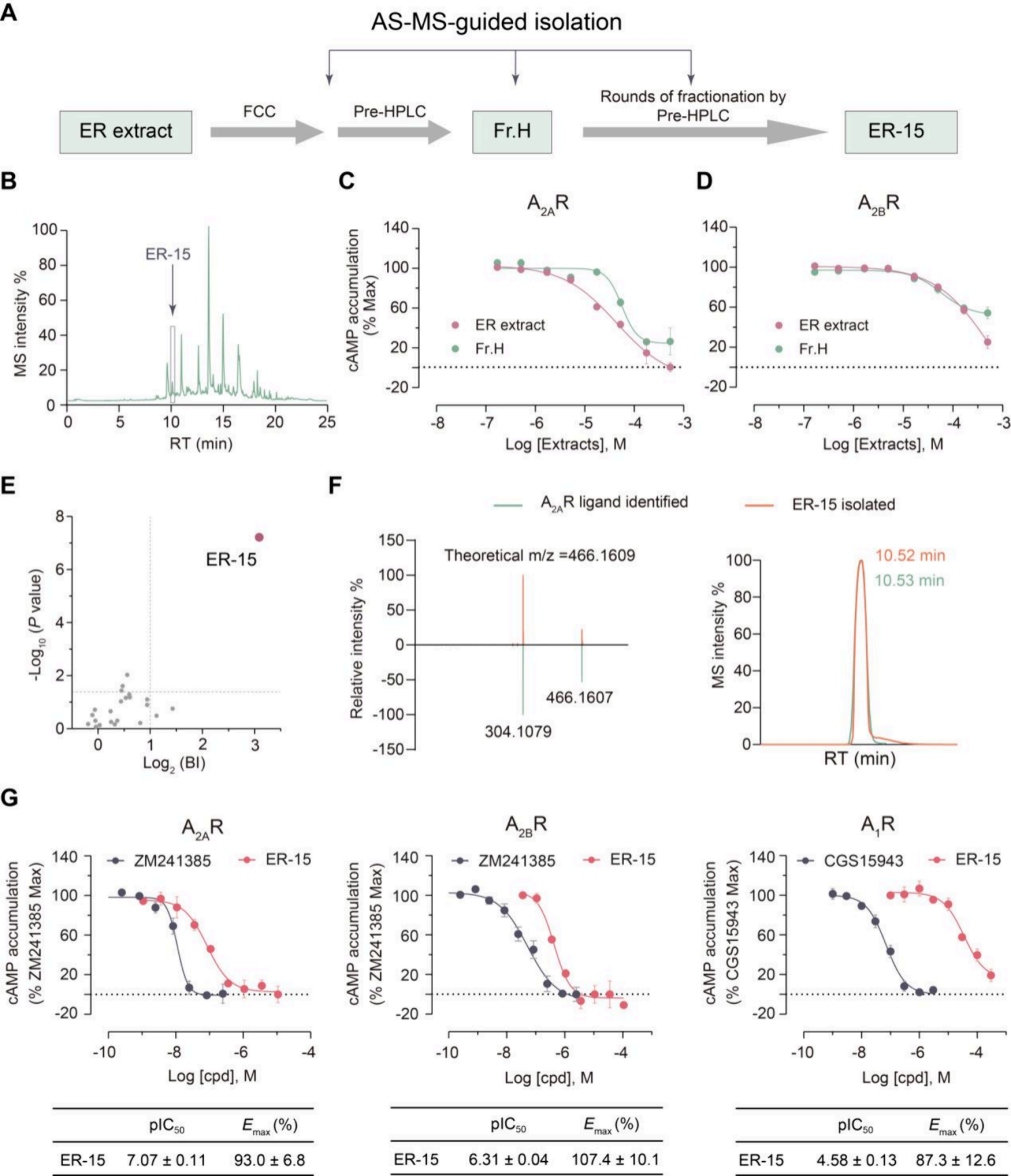
**AS-MS-guided compound isolation and bioactivity measurement of ER-15.** (**A**) The schematic workflow of AS-MS-guided compound isolation. (**B**) Representative TIC of fraction Fr.H indicates presence of ER-15 and other constituents. (**C–D**) Dose–response curves of cAMP accumulation assays in A_2A_R (**C**) or A_2B_R-transfected (**D**) HEK293 cells treated by Fr.H or the ER extract. Data are shown as means ± SEM from three independent experiments. (**E**) AS-MS analysis of Fr.H confirmed ER-15 as the single hit (red dot). BI, binding index. (**F**) Overlaying MS/MS spectra and LC peaks of the isolated compound ER-15 and the A_2A_R ligand identifeid in initial screening. (**G**) Dose–response curves of cAMP accumulation assays in A_2A_R, A_2B_R, and A_1A_R-transfected HEK293 cells treated by ER-15 (red) or a reference compound, with the IC_50_ and *E*_max_ (normalized to reference) values of ER-15 summarized below. Data are shown as means ± SEM from three independent experiments.

Before conducting more extensive fractionation to purify ER-15, we measured the bioactivity of Fr.H which was a mixture of ER-15 and other herbal constituents. To our disappointment, Fr.H exhibited reduced activity at A_2A_R and similar activity at A_2B_R compared to the ER extract (Figures 3C, D), keeping us to wonder whether ER-15 is a false positive hit. At this puzzling stage, we performed AS-MS analysis of Fr. H, which, gratifyingly, yielded ER-15 as a single strong hit with BI > 8 indicating its high affinity to A_2A_R protein (Figure 3E). Thus, we suspected the compromised activity of Fr.H was probably attributed to interfering substances in the subfraction that counteracted the antagonism of ER-15.

Reassured by our AS-MS result, we subjected Fr.H to three additional rounds of fractionation with prep-HPLC, leading to the isolation of 3.2 mg of ER-15 from 2.3 g of the initial ER extract. The chemical structure of ER-15 was confirmed by 1D and 2D NMR analysis (Note S1). Almost identical RT and MS/MS fragmentation was observed for the isolated compound and the putative ligand identified in the AS-MS screen, which verified our purification of the desired ligand from the crude extract (Figure 3F).

In cellular bioactivity assays, ER-15 suppressed cAMP accumulation induced by either adenosine receptor with stronger potency at A_2A_R (IC_50_ = 85.1 nM) than A_2B_R (IC_50_ = 489.8 nM), demonstrating a subtype selectivity profile consistent with the ER extract (Figure 3G). Of note, ER-15 showed much weaker activity at A_1_R (IC_50_ = 26.3 μM) and no antagonistic effect in the forskolin-stimulated cells (Figure 3G, Figure S4A). Moreover, ER-15 showed no antagonistic activity against a panel of 17 additionally tested class A GPCRs (Table S5). We further observed that ER-15’s antagonizing activity against A_2A_R and A_2B_R was in large retained at increased agonist concentrations (Figure S4B, C). Collectively, guided by AS-MS analysis, we successfully purified this low-abundance compound ER-15, and demonstrated its highly selective dual antagonism on A_2A_R and A_2B_R.

To assess the success rate of bioactive NP identification from different screening platforms, we further obtained 10 compounds that were identified by one or two screening approaches (Figure S5). Six compounds were purified through multiround fractionation from the ER extract (see Methods), and four compounds were purchased from commercial sources. Among them, only one compound (ER-5) which was coidentified by virtual screen and AS-MS screen showed weak antagonistic activity at A_2A_R (IC_50_ = 39.7 μM) while the remaining ones were all inactive (Figure S5). Although we cannot rule out the possibility that certain screening hits bind to A_2A_R without affecting receptor-mediated cAMP signaling, our multiplex screening strategy allows prioritization of the most confident hit with desired bioactivity to be isolated at the first place.

### Distinct Binding Mechanism of ER-15

Given that ER-15 is a strong antagonist of A_2A_R with appreciable selectivity against A_2B_R, we first investigated its binding mechanism for the primary target by employing molecular docking based on an antagonist-bound A_2A_R crystal structure^34^ or the AlphaFold3 (AF3) model.^36^ Molecular docking yielded a favorable docking score (−12.58 kcal/mol) and MM-GBSA binding free energy (−95.36 kcal/mol) within the orthosteric pocket of A_2A_R (Figure 4A). AF3 prediction generated a similar docking pose with the same set of interaction residues as observed in the docking model (Figure 4A). Independent of structural modeling, we conducted molecular dynamics (MD) simulations of the ligand binding process as guided by physical interactions, which indicates ER-15 can swim into the same pocket and adopt the AF3-predicted binding pose (Figure 4B, Figure S6A).

**Figure 4:**
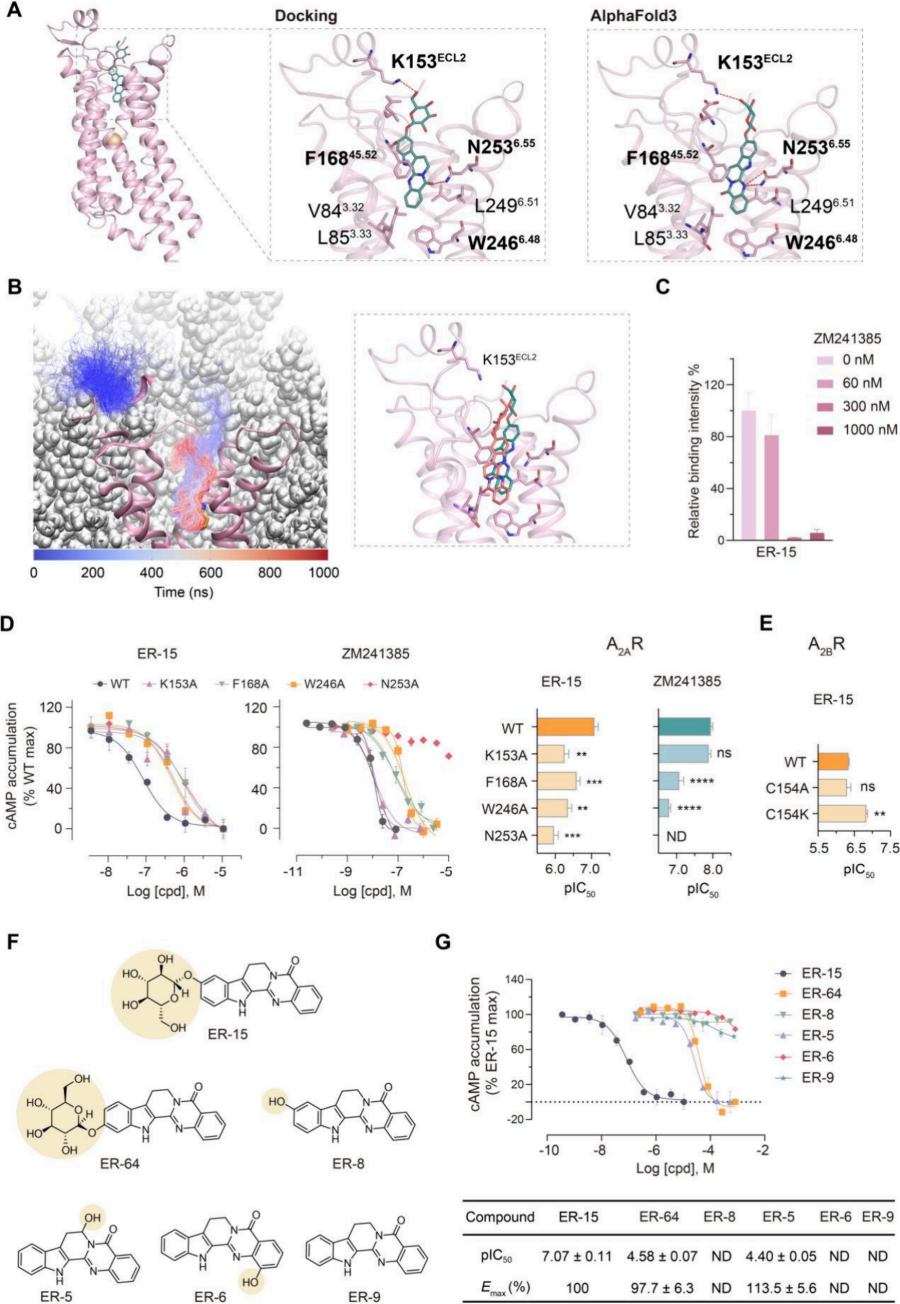
**Revealing the binding mechanism of ER-15.** (**A**) Binding poses of ER-15 generated by molecular docking with an antagonist-bound A_2A_R structure (PDB ID: 4EIY) or AF3 prediction. (**B**) In MD simulations, ER-15 swims into the orthosteric binding pocket of A_2A_R (pink) in the lipid bilayer, arriving at the pose predicted by AF3. The trajectory of ER-15 movement is color-coded from blue to orange along the simulation time (left). In the close-up view (right), the final pose of ER-15 yielded by the swimming simulation (orange) is superimposed with the AF3-predicted pose (green). (**C**) AS-MS-based competition assay of ER-15 binding to A_2A_R protein with increasing concentrations of ZM241385. Relative binding percentages were determined from the MS intensity of ER-15 associated with A_2A_R in the presence of ZM241385 relative to DMSO. (**D**) Dose–response curves of cAMP accumulation assays on ER-15 or ZM241385 in HEK293 cells expressing A_2A_R wild-type (WT) or mutants, with pIC_50_ values shown on the right. (**E**) Antagonistic potency of ER-15 on A_2B_R WT or mutants measured by cAMP accumulation assays. (**F**) The chemical structures of ER-15 analogues for SAR analysis. (**G**) Dose–response curves of cAMP accumulation assays in A_2A_R-transfected HEK293 cells treated by ER-15 and analogues (upper) and corresponding IC_50_ and *E*_max_ values (lower). In (**C-E, F**), data are shown as means ± SEM from three independent experiments. Statistical significance was assessed using a one-way ANOVA with Dunnett’s multiple-comparison test. **P < 0.01, ***P < 0.001, ****P < 0.0001; ns, no significance. ND, no antagonistic activity determined.

In the ER-15 binding mode, its rigid rutaecarpine core occupies the orthosteric pocket by forming a hydrogen bond with N253^6.55^, an aromatic stacking interaction with F168^45.52^, and hydrophobic interactions with residues including V84^3.32^, L85^3.33^, W246^6.48^, and L249^6.51^ (Figure 4A). Of note, while these interactions are shared by several reported A_2A_R antagonists,^37–40^ the unique glucopyranosyl moiety in ER-15 pointing to the extracellular side is engaged in a hydrogen bond with K153 in the receptor’s extracellular loop 2 (ECL2) (Figure 4A). Furthermore, in our all-atom MD simulations of the ER-15-bound A_2A_R complex model, we observed high motions of the glucopyranosyl moiety of ER-15 which could facilitate its intermittent but persistent interaction with K153^ECL2^ (Figure S6B).

To verify the orthosteric binding mode of ER-15, we conducted an AS-MS-based competition assay in which the relative binding intensity of ER-15 to A_2A_R protein was measured in an excess of the orthosteric ligand ZM241385. Binding of ER-15 to A_2A_R was competed off by an increasing amount of ZM241385, supporting that ER-15 occupies the same orthosteric pocket as ZM241385 (Figure 4C). We then performed site-directed mutagenesis to assess the contribution of individual residues in the pocket to the ER-15 antagonism on A_2A_R-induced cAMP signaling. Mutations of antagonist-shared interaction residues including F168A, W246A, and N253A all caused a significant reduction or abolishment in the potency of both ER-15 and ZM241385 compared to the wild-type (Figure 4D and Table S6). In contrast, for the compound-specific residue K153^ECL2^, its substitution to Ala reduced the potency of ER-15 by 6.7-fold yet had no effect on ZM241385, which corroborates the unique interaction between the glucopyranosyl group of ER-15 and K153^ECL2^ (Figure 4D and Table S6). All A_2A_R mutants showed cell surface expression similar to the wild-type (Figure S7).

We also utilized AF3 to model the ER-15 binding mode in the secondary target A_2B_R. For residues V85^3.32^, L86^3.33^, F173^45.52^, W247^6.48^, V250^6.51^, N254^6.55^ in the A_2B_R orthosteric pocket that are conserved within the adenosine receptor subfamily and critical for antagonist binding to A_2A_R, they are predicted to form the same set of key interactions with the rutaecarpine core of ER-15 (Figure S8A). However, K153^ECL2^ in A_2A_R is not conserved and corresponds to residue C154^ECL2^ in A_2B_R (Figure S8B). Interestingly, in the modeled structure, the ECL2 region of A_2B_R demonstrates increased flexibility than that of A_2A_R, and C154^ECL2^ in A_2B_R appears distant from ER-15, making it unlikely to form any interaction with the compound (Figure S8A). As expected, the mutation C154A did not affect the ER-15 antagonistic activity on A_2B_R (Figure 4E). Remarkably, substitution of C154^ECL2^ to K was able to enhance the weak potency of ER-15 on A_2B_R by 3.0-fold compared to wild-type, to a level approximating its potency on A_2A_R (Figure 4E). Both C154A and C154 K mutants showed cell surface expression similar to the wild-type (Figure S7), Collectively, these results indicate that although the binding mechanism of ER-15 is mainly conserved between A_2A_R and A_2B_R, its stronger activity on A_2A_R is likely contributed by the unique interaction with K153^ECL2^.

In addition, we conducted structure–activity relationship (SAR) analysis to verify the binding model underlying the ER-15 antagonism, focusing on the role of its glucopyranosyl moiety. Five ER-15 analogues retaining the core scaffold of ER-15 but with repositioned, replaced or removed glucopyranosyl groups were isolated from ER or purchased (Figure 4F). Repositioning this moiety from C-10 to C-11 (ER-64) reduced antagonistic potency against A_2A_R-mediated cAMP signaling by over 300-fold, while hydroxyl group substitution (ER-8) abolished activity (Figure 4G). Further modifications, including glucopyranosyl removal or hydroxyl repositioning (ER-5, ER-6, ER-9), either substantially diminished or eliminated antagonism (Figures 4F, G). Thus, our SAR analysis confirms the glucopyranosyl’s critical role in suppressing the adenosine receptor activation by ER-15.

### ER-15 Enhances T Cell Activation and Cancer Cell Elimination

Activation of adenosine receptors A_2A_R and A_2B_R is known to profoundly impair T cell activation.^22,23^ To assess the impact of ER-15 on this process, we first utilized the human T lymphocyte cell line (Jurkat T cells) stimulated with ionomycin (Iono) and phorbol 12-myristate 13-acetate (PMA).^28^ Consistent with previous findings, the adenosine receptor agonist NECA significantly suppressed the expression of granzyme B (GZMB), a key marker of T cell activation and cytotoxic function.^41,42^ Importantly, compound ER-15 treatment dose-dependently restored the GZMB expression, effectively counteracting the inhibitory effect of NECA (Figure 5A). In parallel, NECA markedly up-regulated the expression of immunoinhibitory receptors PD-1 and TIM-3 ^43^ (both *P* < 0.001), whereas ER-15 almost completely abolished this NECA-induced elevation of PD-1 and TIM-3 expression (Figures 5B, C). Of note, the efficacy of ER-15 in these T cell activation assays was comparable to that of AB928, a dual A_2A_R/A_2B_R antagonist in phase II clinical trial, at the same dose (10 μM).

**Figure 5:**
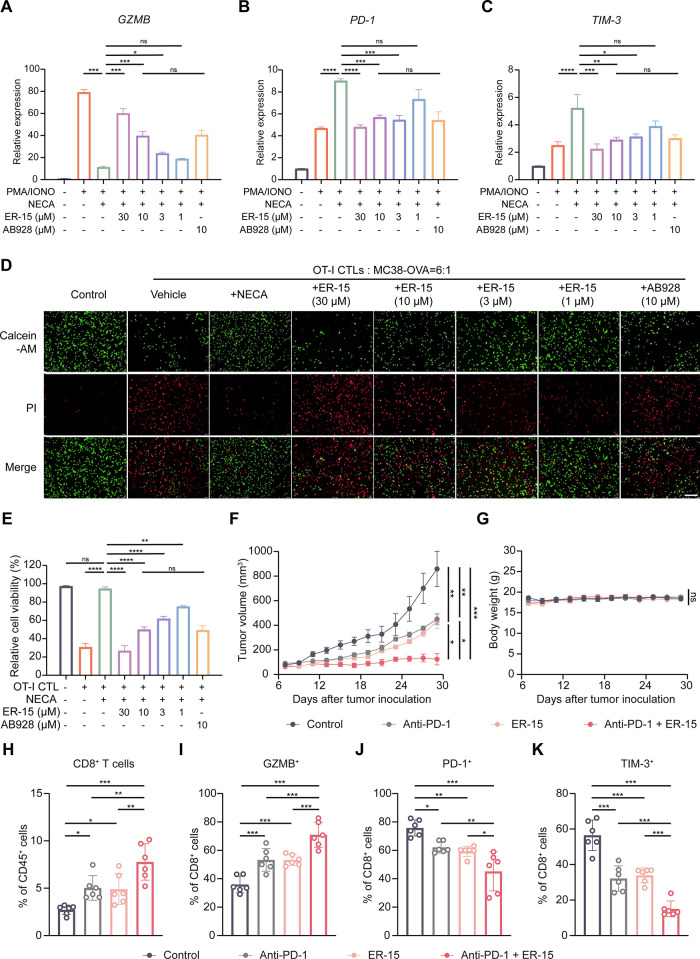
**ER-15 enhances T cell effector functions and induces robust antitumor immune responses.** (**A-C**) Q-PCR analysis of T cell activation markers in an *in vitro* stimulation model. Human Jurkat T cells were stimulated with ionomycin (Iono,1 μg/mL) and PMA (20 ng/mL) in the presence of NECA (1 μM) with increasing concentrations of ER-15 or a phase II trial drug AB928 for 5 h. Transcript levels of GZMB (**A**), PD-1 (**B**) and TIM-3 (**C**) were measured by Q-PCR. (**D**) Representative fluorescence images of OT-I CTLs cocultured with MC38-OVA cancer cells. Cocultures were treated with NECA (1 μM) with or without ER-15 or AB928. Live cancer cells were stained with Calcein-AM (green) and dead cells with propidium iodide (red). Scale bar = 200 μm. (**E**) Quantification of the relative killing ability of OT-I CTLs in (**D**). (**F**) Growth curves in MC38 tumor-bearing C57BL/6 mice at day 7 to day 29 after inoculation (*n* = 6). C57BL/6 mice inoculated with MC38 cancer cells were injected intraperitoneally with vehicle, 50 mg/kg ER-15, 2.5 mg/kg anti-PD-1 antibody, or their combination starting at day 7 after inoculation. (**G**) Body weight curves of MC38 tumor-bearing C57BL/6 mice treated as in (**F**). (**H–K**) Flow cytometry analysis of the frequency of CD8^+^ T cell gated on CD45^+^ cells (**H**), and the frequency of GZMB^+^ cells (**I**), PD-1^+^ cells (**J**) and TIM-3^+^ cells (**K**) gated on CD45^+^CD8^+^ T cells as in (**F**). Data were from three independent experiments.

We next assessed the effect of ER-15 on T cell-mediated tumor cell cytotoxicity in a coculture system. Naïve OT-I cytotoxic T lymphocytes (CTLs) were cocultured with MC38-OVA cells—a murine colon carcinoma line stably expressing ovalbumin and presenting the SIINFEKL peptide recognized by OT-I CTLs.^44^ As expected, NECA treatment substantially impaired OT-I CTLs–mediated killing of MC38-OVA cancer cells as revealed by live/dead cell imaging (Figures 5D, E). Strikingly, compound ER-15 robustly restored OT-I CTLs cytotoxicity under NECA-induced suppression, thereby enhancing the elimination of MC38-OVA cancer cells (Figures 5D, E). Of note, the alkaloid extract of ER showed evident cytotoxicity toward OT-I CTLs (IC_50_ = 0.9 μM) and MC38-OVA cells (IC_50_ = 3.7 μM). But the isolated compound ER-15 exhibited negligible direct cytotoxicity (IC_50_ > 100 μM) and had no effects on the cell cycle or apoptosis for both cell types (Figures S9A-H).

### ER-15 Induces Antitumor Immunity and Improves the Efficacy of the Anti-PD-1 Therapy

Encouraged by our T cell activation and killing assay results, we sought to assess the *in vivo* antitumor efficacy of ER-15. To this end, immunocompetent C57BL/6 mice were subcutaneously implanted with MC38 colon cancer cells and intraperitoneally administrated with ER-15 beginning on day 7 postinoculation. Of note, ER-15 treatment significantly suppressed the tumor growth compared to vehicle-treated controls, as measured by tumor volume (Figure 5F and Figure S10A). Flow cytometry analysis of MC38 tumors revealed 1.8-fold more CD8^+^ T cells, 17.2% more GZMB^+^CD8^+^ T cells, 16.5% fewer PD-1^+^CD8^+^ T cells and 22.6% fewer TIM-3^+^CD8^+^ T cells in ER-15-treated mice compared to vehicle-treated controls (Figures 5H–K and Figure S10B). These findings indicate that ER-15 enhances CD8^+^ T cell–mediated anticancer immunity in the tumor microenvironment.

We then investigated whether ER-15 improves the efficacy of the immune checkpoint blockade therapy. As reported previously, mice bearing MC38 tumors were only modestly responsive to the anti-PD-1 monotherapy.^45^ Indeed, monotherapy with ER-15 or anti-PD-1 marginally inhibited tumor growth by 41.4% or 38.6%. Remarkably, the combination of ER-15 and anti-PD-1 treatment significantly reduced tumor growth by 85.4% (Figure 5F and Figure S10A). In addition, there was no significant alteration in the mouse body weight in any groups (Figure 5G). Flow cytometry analysis of tumors from the combination therapy group showed a 1.5-fold increase in frequency of CD8^+^ T cells and a 17.6% increase in GZMB^+^CD8^+^ T cells. This was accompanied by a reduction of 16.9% and 17.1% in the proportions of PD-1^+^ and TIM-3^+^ CD8^+^ T cells, respectively, compared to anti-PD-1-treated mice (Figures 5H–K). Immunofluorescence analysis further showed that treatment with ER-15 alone or in combination with anti-PD-1 led to an increased infiltration of progenitor-exhausted CD8^+^ T cells (Tpex; CD8^+^TCF-1^+^) and a concomitant decrease in terminally exhausted CD8^+^ T cells (Ttex; CD8^+^TIM-3^+^) (Figure S10C). These findings demonstrate that ER-15 is able to synergize with the anti-PD-1 therapy to enhance the antitumor immunity and thus improve the therapeutic efficacy *in vivo*.

### ER-15 Arguments the Efficacy of the Anti-PD-1 Therapy in Patient-Derived Tumor Organoids (PDOs)

Patient-derived tumor organoids (PDOs) faithfully recapitulate the histopathological architecture and immune cell repertoire of the original tumor microenvironment, enabling patient-specific modeling of the immunotherapeutic responses.^46^ To further validate the translational relevance of our findings, we generated PDOs from surgically resected primary colorectal carcinoma specimens using the air–liquid interface method.^47^ Histological analysis confirmed that these organoid cultures preserved the characteristic architecture of their parental tumors (Figure S11A). Furthermore, flow cytometry analysis demonstrated that CD8^+^ T cells represented approximately 10% of the CD45^+^ immune compartment within PDOs (Figure 6A and Figure S11B).

**Figure 6:**
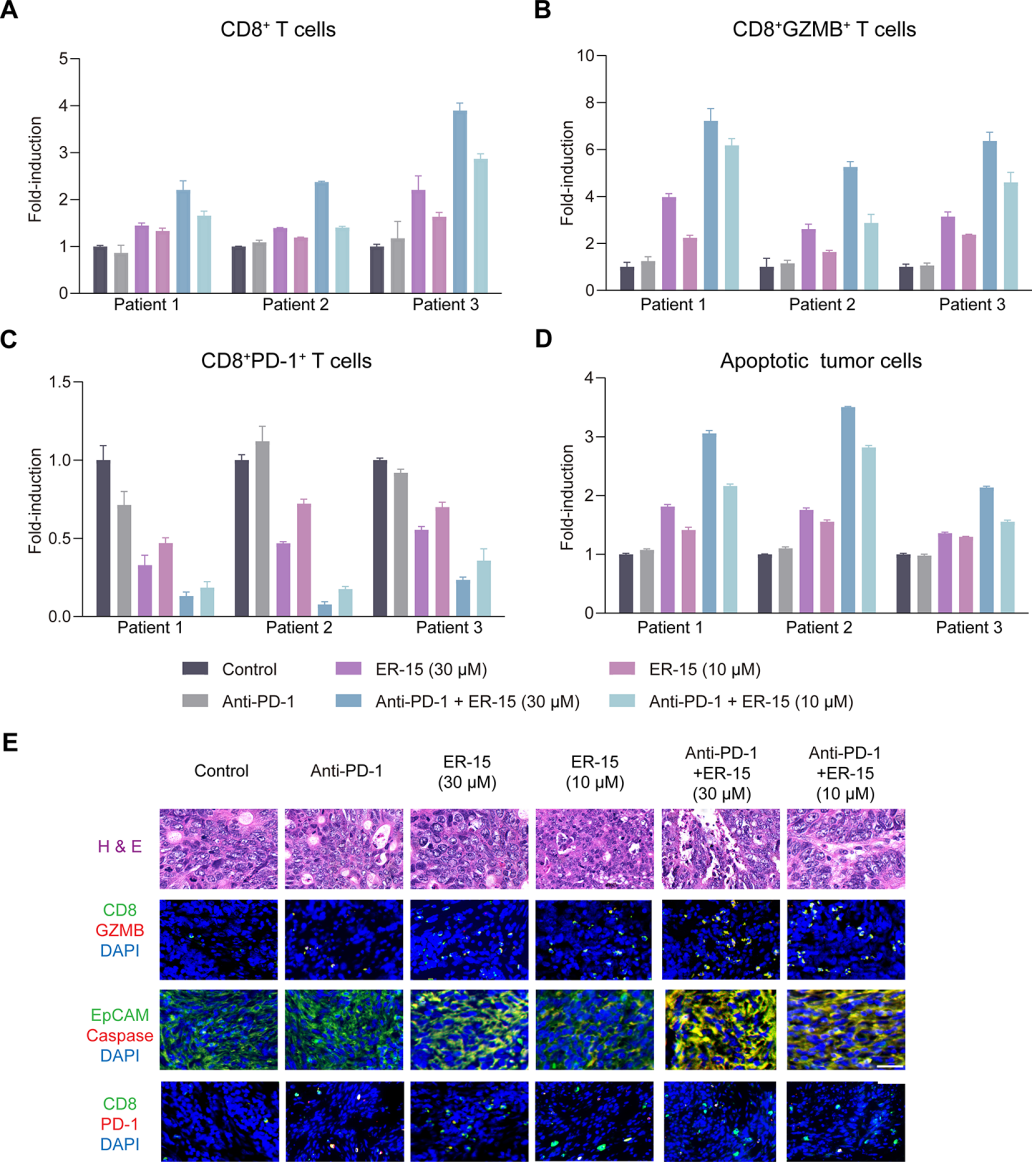
**ER-15 improves the efficacy of anti-PD-1 therapy in PDOs.** (**A-D**) PDOs were established for human colorectal cancer biopsies and were treated with vehicle, ER-15 (30, 10 μM), anti-PD-1 antibody (Pembrolizumab, 5 μg/mL), or their combination for 5 days. Flow cytometry analysis reveals the relative changes in the frequencies of CD8^+^ T cells (**A**), GZMB^+^CD8^+^ T cells (**B**), and PD-1^+^CD8^+^ T cells (**C**) gated on CD45^+^ cells, and the frequencies of annexin-V^+^ cells among EpCAM^+^ tumor cells (**D**) in PDOs. Data in (**A-D**) were from two to three independent experiments. (**E**) Representative images of histological and immunofluorescence analysis of PDOs treated with vehicle, ER-15 (30, 10 μM), anti-PD-1 antibody (Pembrolizumab, 5 μg/mL), or their combination. Scale bar = 40 μm. Data in (A-D) were from two to three independent experiments.

We next evaluated therapeutic interventions by treating PDOs for 5 days with ER-15, anti-PD-1 (pembrolizumab), or their combination. Anti-PD-1 monotherapy failed to augment CD8^+^ or GZMB^+^CD8^+^ T cell frequencies and did not reduce PD-1^+^CD8^+^ T cell proportions (Figures 6A-C, E). In contrast, ER-15 alone significantly expanded the GZMB^+^CD8^+^ T cell subset and lowered PD-1^+^CD8^+^ T cell proportions, concomitant with a pronounced increase in EpCAM^+^ tumor cell apoptosis vs vehicle (Figures 6B-E). Remarkably, coadministration of ER-15 and anti-PD-1 into PDOs elicited a synergistic enhancement of CD8^+^ T cell activation and potent tumor cell killing—far exceeding the effects of either agent alone (Figures 6A-E). Together, these data nominate ER-15 as a promising candidate to potentiate the anti-PD-1 immunotherapy in patient-derived models.

## Discussion

In this study, we established a target-centric multiplexed NP screening platform. While virtual screening predicts ligands bound to a specific target pocket, AS-MS captures ligands that may bind any sites on the target protein. On the other side, metabolomics profiling traces potential active compounds in complex mixtures by correlating subfraction metabolite profiles with bioactivity. Each approach individually generates numerous hits (typically tens to over one hundred), making candidate prioritization from a single method challenging. By intersecting hits identified across all three methods, we reduced false positives and prioritized the highest-confidence hit (ER-15) for subsequent isolation and characterization. Notably, ten other hits identified by one or two approaches displayed much weaker or no activity compared to ER-15, which validates our prioritization strategy. Furthermore, AS-MS analysis played a vital role, beyond initial screening, in guiding the isolation of bioactive NPs by enabling real-time confirmation of the presence of the A_2A_R ligand during multiround fractionation.

By multiplexed screening of the total alkaloid extract from a TCM herb *Evodia rutaecarpa* (ER), we identified a new-scaffold A_2A_R/A_2B_R dual antagonist which robustly induces anticancer immune responses in the tumor microenvironment. Preclinical research investigating ER for potential anticancer applications has concentrated on its major alkaloid constituents, evodiamine and rutaecarpine.^48,49^ These compounds demonstrate antitumor effects, including inducing apoptosis, inhibiting proliferation, invasion, and metastasis of cancer cells.^48,49^ However, both ER-containing TCM products and isolated major constituents raise significant safety concerns, especially regarding potential hepatotoxicity, cardiotoxicity, and drug interactions.^50,51^ Our study discovered a trace constituent in ER, ER-15, which specifically targets A_2A_R/A_2B_R to reverse immunosuppression within the tumor microenvironment. ER-15 is an alkaloidal glucoside initially isolated from nearly ripe fruits of ER (commonly known as ‘WuZhuYu’) without any bioactivity evaluation.^35,52^ To our knowledge, ER-15 is the first natural product that is characterized as an adenosine receptor antagonist and thus functions as an immune checkpoint inhibitor. Although ER-15 showed *in vitro* antagonizing activity 8- or 80-fold weaker than the synthetic clinical compound AB928 (IC_50_ = 11.2 nM for A_2A_R, 6.4 nM for A_2B_R),^28^ we were able to validate the *in vivo* antitumor efficacy of ER-15, particularly synergistic activity with anti-PD-1 therapy, in both animal and patient-derived tumor models.

Conventional phenotypic screening of TCM herbal extracts for T cell activation faces significant challenges: crude extracts often exhibit broad cytotoxicity as shown for the ER extract in this study, and low-abundance constituents contribute minimally to the overall activity. Consequently, it would be impossible to identify a minor active component like ER-15 from the crude extract in such screens. Our study therefore provides new insights into the multitarget anticancer mechanism of a TCM herb by revealing the potent immunomodulatory effect of a minor component in contrast to the cancer cell-killing effects of the major constituents. Furthermore, the distinct binding mode revealed for ER-15 engaging both the orthosteric pocket and ECL2 establishes a structural foundation for future optimization and drug development.

In conclusion, we envision that this multiplexed screening strategy can be generalized to accelerate the discovery of structurally unique natural products with target-annotated bioactivities, enabling their development into novel drugs against GPCRs or other challenging targets.

## Experimental Procedures

### Herbal Extract Preparation

Thirteen TCM herbs were first air-dried and pulverized into powder. The powder (500 g each) was extracted with 750 mL of 85% ethanol by water bath ultrasonication for 30 min three times. Following vacuum filtration, the solvent was removed by vacuum evaporation at 50 °C. The residual material represents the total extract (TE) of this herb.

For six herbs, the residual material was acidified to pH 1.0 with 0.3% (v/v) hydrochloric acid and extracted with ethyl acetate (EtOAc) three times. The EtOAc phase was designated as EP. Then, the aqueous layer was basified with 5% (v/v) ammonia to pH 10.0 and partitioned with EtOAc three times. The resulting mixture was partitioned into an EtOAc phase (total alkaloid, TA) and an aqueous phase (AP). The EtOAc phase was evaporated to dryness, and the powder was stored at −80 °C. For the other seven herbs, the residual material was extracted with EtOAc, and the resulting mixture was partitioned into an EtOAc phase (EP) and an aqueous phase (AP).

The stock solution (100 mg/mL) of each crude extract was prepared by dissolving the powder in DMSO and was stored at −20 °C. All solvents are HPLC-grade purity (Tianjin Concord Technology, China). Information regarding the herb names and the 30 extracts prepared from specific herbs are detailed in Table S1.

### cAMP Accumulation Assay

A_2A_R or A_2B_R-mediated cAMP accumulation was measured using a TR-FRET cAMP kit (Cisbio, 62AM4PEC, France) following the manufacturer’s instructions. In brief, HEK-293 cells were transfected with wild-type (WT) A_2A_R or A_2B_R using calcium phosphate transfection. Twenty-four h post-transfection, cells were resuspended with Dulbecco’s modified Eagle’s medium (DMEM, Gibco, USA) supplemented with 1% (v/v) dialyzed FBS (Gibco, USA). Then, cells were seeded into a 384-shallow well plate at a density of 2500–3000 cells per well. Purified compounds or herbal extracts to be tested were diluted to a 4 × final concentration with 1 × Hank’s Balanced Salt Solution (HBSS, Gibco, USA) and 0.1% (w/v) bovine serum albumin (BSA, Sigma-Aldrich, USA). Cells were incubated with purified compounds or herbal extracts for 15 min followed by addition of NECA (5′-N-Ethylcarboxamidoadenosine) at a final concentration of 2.5 nM for A_2A_R assays, 500 nM for A_2B_R assays or 30 μM forskolin for untransfected cells for 15 min at room temperature (RT). Reactions were terminated with lysis buffer containing d2-labeled cAMP and anti-cAMP cryptate. Plates were incubated for 1 h at RT and time-resolved FRET signals were measured using an EnVision plate reader (PerkinElmer, USA) at 665 and 620 nm. Data were presented and analyzed using GraphPad Prism 10.4.1 (GraphPad Software, USA). In cAMP accumulation assays of A_2A_R mutants, HEK293 cells were transfected with mutants and treated with compounds with the same procedure as for WT, except that the NECA concentration was changed to 2.5 nM for mutant K153A, 50 μM for mutant F168A, 20 nM for mutant W246A, and 5 μM for mutant N253A. In assays of A_2B_R mutants, HEK293 cells were transfected and treated with compounds with the same procedure as for WT.

A_1_R-mediated cAMP accumulation was measured with Promega’s Glosensor cAMP biosensor assay according to the manufacturer’s instructions. In brief, CHO-K1 cells transfected with wild-type A_1_R were resuspended with Ham’s F12 and 1% dialyzed FBS and then were seeded into poly­(lysine)-coated 384-well plates at a density of 10000 cells per well. The next day, the supernatant was removed and 20 μL of luciferin diluted with 1 × HBSS was added to cells followed by 1 h incubation at 37 °C. Purified compounds (4 ×) or herbal extracts (4 ×) to be tested were incubated for 15 min followed by addition of NECA (200 nM) or forskolin (30 μM). Chemiluminescence was measured using an EnVision plate reader (PerkinElmer, USA) immediately. For determination of antagonist concentration–response curves, data were normalized to percentages of WT signals and IC_50_ values were yielded by GraphPad Prism 10.4.1 (GraphPad Software, USA).

### Tango Assay

Tango assay was performed to measure β-arrestin2 recruitment for a panel of GPCRs following a previously described protocol.^53^ Briefly, HTLA cells (about 40,000 cells per well) were transfected with Tango plasmids encoding the corresponding GPCRs using Lipofectamine 2000 in poly-l-lysine coated 96-well white clear-bottom plates. After 24 h of transfection, culture media was replaced with 1% DMEM containing 1% dialyzed FBS. Following that, cells were treated with 20 μL ER-15 dilution buffer at a final concentration of 10 μM for 15 min prior to agonist stimulation. The plates were then incubated overnight at 37 °C. The next day, culture media were decanted and 100 μL BrightGlo reagent (Promega) was added to each well. After 20 min incubation in the dark at room temperature, luminescence was read on an Envision plate reader (PerkinElmer). Cells treated with 0.1% DMSO buffer instead of ER-15 dilution buffer served as a negative control.

### Receptor Surface Expression Measurement

The cell surface expression levels of WT and mutants of A_2A_R or A_2B_R were determined using flow cytometry. HEK293 and CHO-K1 cells were transfected with FLAG-tagged plasmids encoding specific receptors or their mutants. Twenty-four h post-transfection, cells were collected and fixed with 4% paraformaldehyde for 20 min at RT. Cells were blocked with 3% BSA for 20 min at RT and then incubated with monoclonal M2 anti-FLAG–FITC (Sigma-Aldrich, USA, F4049; 1:500 diluted with PBS) for 30–60 min at RT in the dark. Cells were washed with PBS twice, and data were collected on a CytoFLEX Platform (Beckman, USA) and analyzed with FlowJo software (v10.8.1, BD Biosciences, USA).

### Virtual Screening

The high-resolution crystal structure of A_2A_R bound with an antagonist ZM241385 (PDB code: 4EIY) was prepared using Protein Preparation Wizard in Maestro 2021–4 (Schrödinger, USA). A grid box covering the entire ZM241385 molecule was selected as the orthosteric pocket of A_2A_R and generated using Receptor Grid Generation module. All compounds in our ER-focused library were prepared using LigPrep with default settings. Docking calculation was performed using Glide with SP precision. Protein–ligand interactions were analyzed with Maestro Ligand Interaction Diagram Panel. For docking hit selection, 36 compounds with top 10% docking scores were retained. Then MM-GBSA was used to calculate binding free energy for each docking pose. Reference ligands ZM241385 and theophylline were treated with the same procedure, which had binding free energy (MM-GBSA dG Binding) of −81.26 kcal/mol and −54.70 kcal/mol, respectively. Compounds with binding free energy lower than −54.70 kcal/mol were selected for visual inspection, finally yielding 28 docking hits.

### A_2A_R Expression and Purification

Construction, expression, and purification of A_2A_R and the control protein HCAR_2_ have been described previously.^54^ In brief, the A_2A_R construct (residues 2–316) comprises a hemeagglutinin signal peptide, a FLAG tag at the N-terminal end, and a 10 × His tag at the C-terminal end. The third intracellular loop (ICL3) was replaced by thermostabilized *E. coli* apocytochrome b562RIL (BRIL).^55^ The HCAR_2_ construct (residues 1–363) comprises a FLAG tag at the N-terminal end, and a 10 × His tag at the C-terminal end. Its ICL3 was also replaced by BRIL. Both constructs were cloned into a pFastbac1 vector (Invitrogen, USA) and expressed in *Spodoptera frugiperda* (*Sf*9) insect cells using the Bac-to-Bac system (Invitrogen, USA). *Sf*9 cells were infected at a cell density of 2–3 × 10^6^ cells/mL and were harvested by centrifugation after infection for 48 h and stored at −80 °C for future use. Insect cell membranes were thawed and dounced homogeneously once in hypotonic buffer and three times in hypertonic buffer, solubilized with *n*-dodecyl-β-D-maltoside (DDM) and cholesteryl hemisuccinate (CHS), and eluted with imidazole, as described previously.^34,54^ Protein buffer was exchanged to the stock buffer (25 mM HEPES, 800 mM NaCl, 10% glycerol, 0.02% DDM, and 0.004% CHS, pH 7.5) by ultrafiltration. Protein purity and homogeneity were verified by analytical size-exclusion chromatography (aSEC), and the protein stock was then stored at −80 °C.

### AS-MS Screening

Purified protein (3–5 μg) of the target A_2A_R or control HCAR_2_ was immobilized on nickel agarose beads (Sigma-Aldrich, USA) overnight in the incubation buffer (25 mM HEPES (pH 7.5), 800 mM NaCl, 0.025% (w/v) DDM and 0.005% (w/v) CHS) at 4 °C. The protein-immobilized beads were then incubated with the ER extract (0.5 mg/mL), Fr.H (0.5 mg/mL), or a reference mixture (each at 100 nM) in a total volume of 100 μL for 1 h at 4 °C. After removing the supernatant, the beads were washed three times with 150 mM ammonium acetate (pH 7.5). 200 μL of 100% methanol was added to extract the compounds bound to receptors for over 25 min. Finally, compounds were dried in a SpeedVac machine and redissolved in 50% methanol for LC-MS/MS analysis. For affinity selection of each matrix, target and control samples were prepared in four independent replicates.

The LC-MS/MS data were acquired on a Q Exactive mass spectrometer (Thermo Fisher Scientific, USA) coupled to an ACQUITY UPLC system (Waters, USA). For each sample set from affinity selection, we first injected a reference sample (the extract or fraction alone) followed by four pairs of target and control samples. Samples were loaded and separated on ACQUITY UPLC BEH C18 column (1.7 μm, 2.1 × 100 mm, Waters, USA) at a flow rate of 300 μL/min, with mobile phase A of 0.1% formic acid (FA) and mobile phase B of 0.1% FA in acetonitrile. For the reference mixture analysis, the LC gradient was as follows: 0–3 min, 5% B; 3–3.1 min, 5–10% B; 3.1–8 min, 10–25% B; 8–12 min, 25–35% B; 12–14 min, 35–90% B; 14–16 min, 90% B. For AS-MS analysis of the ER extract or subfractions, the LC gradient was as follows: 0–2 min, 5% B; 2–2.5 min, 5–10% B; 2.5–16.5 min, 10–40% B; 16.5–20 min, 40–90% B; 20–22 min, 90% B; 22–22.1 min, 90–5% B; 22.1–25 min, 5% B. Full-scan mass spectra were acquired in the range of 160–1200 *m*/*z* at the resolution of 70,000. Major ESI source parameters were as follows: automatic gain control (AGC) target 3e6, maximum injection time (IT) 200 ms, voltage 3.5 kV; auxiliary gas heater temperature 350 °C; capillary temperature, 320 °C; sheath gas, 35; auxiliary gas flow rate, 10. For MS/MS scans, the top 10 precursors were analyzed with a resolution of 35,000, AGC target of 1e5, maximum IT of 50 ms, and NCE of 20, 40, 60.

Compound assignment and binding index (BI) calculation were performed using previously described procedures.^20,56,57^ In brief, ER-derived compounds in the target (A_2A_R) and control samples (HCAR_2_) were assigned by generating EICs using TraceFinder 4.1 (Thermo Fisher Scientific, USA) according to the accurate mass (< 5 ppm), signal-to-noise ratio (S/N > 10), and RT matching with respective peaks in the reference (< 15 s). Binding index (BI) of each compound is defined as the ratio of EIC responses of the compound detected in the target (A_2A_R) to that in the control (HCAR_2_). Screening hits were identified based on the following criteria: mean BI > 2, *P* < 0.05 (two-tailed Student *t*-test), n = 4.

### Extract Fractionation and Metabolomics Profiling

The powder of the ER crude extract (200 μg) was dissolved in 50% methanol and then fractionated using the same ACQUITY UPLC system (Waters, USA) and the same LC gradient as described in AS-MS screening. Five subfractions collected at a 5 min interval during LC separation are named as F1 to F5. The solvent was removed by vacuum evaporation at 4 °C, and the residue was stored at −80 °C. Later the residue was resuspended in 1 × HBSS (Gibco, USA) and 0.1% (w/v) bovine serum albumin with 4% DMSO for cAMP accumulation assays.

Meanwhile, for metabolomics profiling, subfractions F1 to F5 resuspended in 50% methanol and a solvent blank were injected and analyzed by UPLC-HRMS using the aforementioned method subjected. MS features for ER-derived compounds were assigned by generating EICs using TraceFinder 4.1 (Thermo Fisher Scientific, USA) based on the accurate mass (< 5 ppm), signal-to-noise ratio (S/N > 10), and RT matching with respective peaks in the reference (< 15 s). Then differential analysis of the EIC responses of MS features were conducted between F3 and any of the other fractions (F1, F2, F4, F5) or blank. MS features only detected in F3 or showing a fold-change > 100 over all other fractions and blank were selected as F3-specific features.

### AS-MS-Based Competition Assay

The purified protein A_2A_R (1 μg) was immobilized on nickel agarose beads (Sigma-Aldrich, USA) overnight in the incubation buffer (25 mM HEPES (pH 7.5), 800 mM NaCl, 0.025% (w/v) DDM and 0.005% (w/v) CHS) at 4 °C. The A_2A_R-immobilized beads were then incubated with compound ER-15 (3 μM) in the absence or presence of ZM241385 at an increasing concentration (60 nM, 300 nM, 1 μM) at 4 °C for 1 h. The compounds were then dissociated from the A_2A_R beads, reconstituted, and analyzed by UPLC-HRMS using the aforementioned method. Identification and quantification of ER-15 was achieved by EIC extraction using TraceFinder 4.1 (Thermo Fisher Scientific, USA) based on accurate mass (< 5 ppm), signal-to-noise ratio (S/N > 10), and retention time matching in the reference (< 15 s). Three independent experiments were performed in technical duplicates under each condition. The relative binding percentage of ER-15 was determined from its EIC response in the presence of ZM241385 relative to DMSO.

### Isolation of ER-15

The total alkaloids of ER (2.3 g) were first separated with fast column chromatography on a silica gel column, and eluted with petroleum ether-ethyl acetate (1:0.1–1:1) and dichloromethane-methanol (1:0.1–1:1) to obtain six fractions (Fr.A-Fr.F). Analysis of each fraction by UPLC-PDA/HRMS with the same method for AS-MS screening revealed Fr.F contained the expected feature of ER-15 (*m*/*z* 466.1609, RT 10.5 min). Fraction Fr.F was then loaded and separated using a Shimadzu LC-20A semipreparative HPLC (Pre-HPLC) system (Shimadzu, Japan) on a SunFire C18 OBD Prep column (5 μm, 19 × 250 mm, Waters, USA) at a flow rate of 10 mL/min. The mobile phase consisted of 0.1% trifluoroacetic acid (TFA) in methanol (A) and 0.1% TFA (B). The LC gradient was as follows: 0–0.01 min, 80% B; 0.01–2 min, 80–60% B; 2–15 min, 60–0% B; 15–25 min, 0% B; 25–25.1 min, 0–80% B; 25.1–30.1 min, 80% B; 25.1–35 min, 80% B. The subfractions obtained from Fr.F collected at a 5 min interval were also analyzed by UPLC-PDA/HRMS. One subfraction within the 15–20 min RT range containing the expected feature of ER-15 was designated as Fr.H.

Fr.H was loaded and further separated using the same Pre-HPLC system with the mobile phase A changed to 0.1% TFA in acetonitrile. The LC gradient was as follows: 0–17 min, 65% B; 17–20 min, 65–0% B; 20–25 min, 0% B; 25–25.1 min, 0–65% B; 25.1–35 min, 65% B. Major chromatographic peaks were manually collected and analyzed by UPLC-PDA/HRMS. Only peak fractions containing the expected feature of ER-15 were subjected to iterative Pre-HPLC separation. After three rounds of fractionation tracing the expected feature, the peak eluting at 10.3 min yielded the isolated compound of ER-15 (3.2 mg, > 95% purity). The chemical structure was characterized with 1D (^1^H and ^13^C) and 2D (HSQC, HMBC, ^1^H–^1^H COSY, NOESY) NMR (Avance III HD 800 MHz, Bruker, USA), and data are shown in Note S1.

### Isolation of Other ER-Derived Compounds

The EtOAc phase of ER was dissolved in 90% ethanol to yield a white precipitate that was collected by vacuum filtration to obtain a white powder. The powder (40 mg) was dissolved in DMSO and injected into the same Pre-HPLC system to be separated with the gradient as follows: 0.01–7 min, 80–40% B; 7–20 min, 40–10% B; 20–20.1 min, 10–0% B; 20.1–30 min, 0% B; 30–30.1 min, 0–80% B; 30.1–40 min, 80% B. The mobile phase consisted of 0.1% TFA in methanol (A) and 0.1% TFA (B). Two major peaks (RT at 23.8 and 26.3 min) were collected and dried with vacuum evaporation. After structural elucidation by NMR (400 MHz, see Note S1), we obtained ER-12 (evodiamine, 8.51 mg) and ER-9 (rutaecarpine, 20.53 mg).

The remaining EtOAc phase of ER was similarly fractionated using an Agilent 1260 Infinity Pre-HPLC system as described prevously^58^ to obtain four other ER-derived compounds. In brief, in the first-round fractionation, the EtOAc phase was dissolved in 50% methanol and separated by a modified gradient (0–60 min, 70–0% B; 60–70 min, 0% B). The mobile phase consisted of 0.1% TFA in methanol (A) and 0.1% TFA (B). The major chromatographic peaks were collected into 11 subfractions based on UV absorption at 254 nm. The third to fifth subfractions were subjected to second-round fractionation using a modified gradient (75% methanol in ddH_2_O, 17 min). A major peak (RT 5 min) was collected and designated as ER-60 (dehydroevodiamine, 13.1 mg). Two additional peaks (RT 5.7–10.2 min) were further purified by third-round fractionation with another gradient (51% methanol in ddH_2_O, 30 min). The major peak (RT 21.8 min) was collected and designated as ER-6 (1-hydroxyrutaecarpine, 8 mg). The seventh subfraction was further fractionated using a modified gradient (55% acetonitrile in ddH_2_O, 30 min). A major peak (RT 15.8 min) was collected and designated as ER-33 (14-formyldihydrorutaecarpine, 47.6 mg). The eighth subfraction was further fractionated using another gradient (0–36 min, ddH_2_O/acetonitrile = 44/55; 36–37 min, linearly increase to 95/5; 37–50 min, 95/5). A major peak (RT 15.8 min) was collected and designated as ER-5 (7β-hydroxyrutaecarpine, 15 mg). All chemical structures were confirmed by NMR analysis (500 MHz, see Note S1).

### Purchase of ER-Derived Compounds

ER-56 (skimmianine), ER-58 (obacunone), and ER-63 (higenamine) were purchased from BioBioPha (China), Abphyto (China), and Yongjian Pharmaceutical (China), respectively. ER-64 (rutaecarpine-11-*O-β*-d-glucopyranoside) and ER-8 (10-hydroxyrutaecarpine) were purchased from WuXi AppTec (China).

### Molecular Docking and AF3 Prediction

To predict the binding modes of ER-15 to A_2A_R and A_2B_R, we performed ligand docking and structural prediction using the AF3 pipeline (GitHub). The docking calculations were performed as described in the Virtual screening section above, except that the docking precision was set to the XP mode. For AF3 prediction, the receptor sequence of each model was provided in FASTA format and ER-15 was encoded as a SMILES string. An ER-15-bound A_2A_R model was constructed using the template prepared from the crystal structure^34^ (PDB code: 4EIY) with its BRIL fusion in ICL3 removed. The ER-15-bound A_2B_R model was constructed using the template prepared from the cryo-EM structure^59^ (PDB code: 7XY6). The random model seed was set to 1. The predicted model with the highest ranking score was selected and imported into PyMOL 3.0.3 for ligand binding mode analysis.

### Molecular Dynamics Simulations

In all molecular dynamics (MD) simulations, the receptor was embedded in a lipid bilayer, and all atoms in the system were represented explicitly with solvent. A total of four molecular dynamics simulations were conducted using GROMACS 2023.3 with CHARMM36 force field as detailed below.^60^ Two of these simulations are swimming simulations where a small molecule was initially placed arbitrarily in the solvent and subsequently entered the orthosteric pocket during the simulation. The other two simulations were initiated from the AF3 predicted complex structure. In the simulations, snapshots were saved every 1000 ps to record the coordinates of all atoms at specific time points. Trajectories were visualized and analyzed using VMD 1.9.3.

For simulation starting from the AF3-predicted ER-15–A_2A_R complex, the system was prepared at pH 7.4 using the Protein Preparation Wizard in Maestro (Schrödinger Release 2024–3), with protein termini capped with neutral acetyl and *N*-methylamide groups. The system was solvated in an orthorhombic periodic water box (TIP3P) containing 0.15 M NaCl, with a minimum buffer of 15 Å, and subsequently neutralized. The final system comprised 58,871 atoms, including 152 POPC lipids, 11,215 water molecules, 32 Na^+^ ions, and 31 Cl^–^ ions. CHARMM36 parameters assigned via Viparr were applied to the protein, lipids, and ions, while parameters for ER-15 were generated using the MMFF-based SwissParam web server (https://www.swissparam.ch/).^60–62^ The system was first energy-minimized and then equilibrated following a staged protocol: an initial 100 ps NVT simulation at 10 K with strong restraints on protein heavy atoms to relax water and ions; 2 ns of Brownian method at 100 K, restraining the membrane along the *z*-axis and applying positional restraints on the protein and ligand, with Gaussian forces applied to the membrane region to prevent water permeation; 1 ns at 100 K under an MTK method with light restraints on the membrane and protein heavy atoms; gradual heating from 100 to 300 K over 1.5 ns in an NPgT ensemble, with positional restraints on the protein gradually released; 500 ps at 300 K with weak restraints on the protein backbone and ligand; and 500 ps at 310 K with all restraints removed. Following this pre-equilibration, two independent 500 ns production simulations were conducted in the NPT ensemble (310 K, 1.01325 bar) with a 2 fs integration time step.^63^

For ER-15 swimming simulation, the system was constructed using the CHARMM-GUI web server (https://www.charmm-gui.org/).^64,65^ The prepared receptor was embedded in a pre-equilibrated POPC lipid bilayer, and a single ER-15 molecule was randomly placed in the aqueous phase (TIP3P water model) at least 30 Å away from the binding pocket. Sodium and chloride ions were added to achieve a physiological salt concentration of 0.15 M. The final system was a rectangular box with dimensions of 12.51 × 12.51 × 11.74 nm^3^, comprising a total of 187,890 atoms, including the receptor, 460 POPC lipids, 40,413 TIP3P water molecules, 109 Na^+^ ions, and 119 Cl^–^ ions. All components were parametrized with the CHARMM36 force field, while ligand parameters for ER-15 were generated with SwissParam using an MMFF-based approach.^60,62^ Energy minimization and subsequent molecular dynamics (MD) simulations were performed with GROMACS 2023.3. After minimization, the system was equilibrated following the six-step CHARMM-GUI protocol, in which the first two steps were conducted under an NVT ensemble and the remaining steps under an NPT ensemble, maintaining the temperature at 310 K. The simulations were carried out in the NPT ensemble at 310 K, using a 2 fs integration time step, the v-rescale thermostat, and semi-isotropic C-rescale pressure coupling.^66,67^ Bond constraints involving hydrogen atoms were treated with the LINCS algorithm, long-range electrostatics were calculated using the particle mesh Ewald (PME) method and van der Waals interactions were handled with a force-switch cutoff scheme.^68,69^ To preserve the spatial relationship between ligand and receptor, a flat-bottom potential (k = 500 kJ·mol^–1^·nm^–2^) was applied to restrain the ligand relative to a reference group. Two independent 1 μs production runs were performed for each system.

### T Cell and Cancer Cell Culture

Human Jurkat T cells and MC38 mouse colon cancer cells were purchased from the Cell Bank of the Chinese Academy of Sciences (Shanghai, China). The MC38-OVA cells were generated by overexpressing chicken ovalbumin. Jurkat T cells maintained in RPMI 1640 medium (Gibco, USA), while MC38 and MC38-OVA cells were cultured in DMEM medium (Gibco, USA), supplemented with 10% fetal bovine serum (FBS, Biotech, USA) and 1% penicillin-streptomycin solution (Gibco, USA). The cells were maintained in a humidified incubator at 37 °C with 5% CO_2_.

### Mice

C57BL/6 mice (female, 6–8 weeks old) were obtained from the National Rodent Laboratory Animal Resources (Shanghai, China). OT-I mice were kind gifts from Professor Zhengfang Yi (East China Normal University). All mice were maintained in a specific pathogen-free conditions, housed in groups at a controlled temperature of 20–22 °C and humidity of 60%, with a 12 h light/dark cycle. All animal experiments were conducted in accordance with a protocol that had been approved by the Laboratory Animal Welfare and Ethics Committee of East China Normal University (Approval No. m20250802).

### Q-PCR

Jurkat T cells were seeded into a 12-well plate and pretreated with ER-15 or AB928 for 0.5 h, followed by stimulation with 1 μM NECA for an additional 4 h. Total mRNA was extracted using TRIzol reagent (Invitrogen, USA). cDNA was synthesized using the Hifair II first Strand cDNA Synthesis SuperMix (Yeasen, China) following the manufacturer’s instructions. Real-time PCR was performed using Hieff qPCR SYBR Green Master Mix (Low Rox Plus) (Yeasen, China) with specific primers. GAPDH was used as an internal control for normalization. The primers used in this study are listed as follows:

*GAPDH*-forward primer (AATCCCATCACCATCTTCCA)

*GAPDH*-reverse primer (TGGACTCCACGACGTACTCA)

*GZMB*-forward Primer (TACCATTGAGTTGTGCGTGGG)

*GZMB*-reverse Primer (GCCATTGTTTCGTCCATAGGAGA)

*PD-1*-forward primer (CCAGGATGGTTCTTAGACTCCC)

*PD-1*-reverse primer (TTTAGCACGAAGCTCTCCGAT)

*TIM-3*-forward primer (AGACAGTGGGATCTACTGCTG)

*TIM-3*-reverse primer (CCTGGTGGTAAGCATCCTTGG).

### T Cell-Mediated Killing Assay

OT-I cytotoxic T lymphocytes were isolated from the spleens of 8-week-old female OT-I mice. Briefly, the spleens were harvested and dissociated into a single-cell suspension by filtering through a 40-μm cell strainer. The splenocytes were counted after red blood cell lysis and cocultured in a 12-well plate with preseeded MC38-OVA cells at a ratio of 6:1. After 48 h, the cells were stained with Calcein AM and PI (Yessen, China) to distinguish live and dead cells. Live/dead images were recorded via an OLYMPUS fluorescent microscope and processed by ImageJ (v1.54p; NIH, USA).

### MTT Assay

Cell viability was assessed using the MTT (3-(4,5-dimethylthiazol-2-yl)-2,5-diphenyl tetrazolium bromide) assay. Briefly, MC38-OVA cells (2,000 cells per well) or OT-I cytotoxic T lymphocytes (10^5^ cells per well) were seeded into a 96-well plate and cultured overnight. The cells were then treated with vehicle or various concentrations of ER-15 for 48 h. Subsequently, 20 μL of MTT solution was added to each well and incubated for 2 h. Cell viability was then assessed by measuring the absorbance at 490 nm by a Cytation 5 imaging reader (BioTek, USA).

### Syngeneic Mouse Models

Syngeneic mouse tumor models were generated by subcutaneously injecting mouse MC38 colon cancer cells. The cells were suspended at a density of 1 × 10^6^ in 100 μL PBS and implanted into the back of the mice. When the tumor volume reached approximately 50–100 mm^3^, the mice were randomly assigned to different drug treatment groups. The tumor size and body weight were measured every 2 or 3 days. The tumor volume was calculated using the formula 0.5 × length × width × width.

### Human Clinical Specimens

Colorectal cancer biopsy samples were obtained by The Fifth People’s Hospital of Shanghai, Fudan University. Written informed consent was acquired from all participants prior to sample collection. The study was approved by the Institutional Review Board (Ethics Committee for Medical Research) of The Fifth People’s Hospital of Shanghai, Fudan University (Ethics Review Approval No. 172 (2024)). Detailed participant information is provided in the Table S7.

### Human PDO Culture

Collagen gel matrices were preprepared by mixing collagen matrix (Rat Collagen I), sterile reconstitution buffer (2.2 g NaHCO_3_ in 100 mL of 200 mM HEPES and 0.05 N NaOH) and 10 × concentrated culture medium (Ham’s F-12, Invitrogen, USA) in a ratio of 8:1:1 on ice until use. Under sterile conditions, inserts (Millipore, USA) were placed into 24-well plate. Subsequently, 300 μL of collagen gel matrix was added to the insets to form a tissue-free base layer. The bottom layer was allowed to solidify for 0.5 h in a 37 °C incubator.

Tumor tissues were minced finely on ice and washed twice in ADMEM/F12 medium (Invitrogen, USA) supplemented with 10 × penicillin/streptomycin (Invitrogen, USA) and 1 × Normocin (InvivoGen, USA). The minced tumor fragments were resuspended in 300 μL of collagen gel matrix and layered on top of the presolidified base layer to establish the double-layer air–liquid culture system. The insert containing the tumor tissue and collagen was placed into an outer 24-well plate containing 500 μL of medium (ADMEM/F12 supplemented with RSPO1 (500 ng/mL, Yeasen, China), Wnt3a (10 ng/mL, MCE, China), Noggin (100 ng/mL, Invitrogen, USA), HEPES (1 mM, Invitrogen, USA), Nicotinamide (10 mM, MCE, China), Glutamax (1 ×, Invitrogen, USA), B-27 without vitamin A (1 ×, Invitrogen, USA), N-Acetylcysteine (1 mM, Sigma-Aldrich, USA), Gastrin I (10 nM, Sigma-Aldrich, USA), Pen-Strep Glutamine (1 ×, Invitrogen, USA), SB-202190 (10 mM, MCE, China), A83–01 (0.5 mM, MCE, China), and EGF (50 ng/mL, Invitrogen, USA)). The cultured organoids were treated with vehicle, ER-15 (10, 30 μM), anti-PD-1 antibody (Pembrolizumab, 5 μg/mL, Sigma-Aldrich, USA), or their combination, and were collected after 5 days.

### Flow Cytometry Analysis

Tumor tissues were collected, minced, and digested in FBS-free RPMI 1640 medium containing collagenase I (10 U/ml, Gibco, USA) and collagenase IV (400 U/ml, Gibco, USA) for 30 min at 37 °C. The digested tumor tissues were passed through 40-μm cell strainers (Falcon, USA) to obtain single-cell suspensions. The single-cell suspensions were washed with PBS containing 2 mM EDTA (Sangon, China) and 1% FBS. The cells were then incubated with anti-CD16/32 FcR blocking antibody (Biolegend, USA) for 10 min, followed by staining with 20 μg/mL fluorescence-conjugated antibodies specific to plasma-membrane markers for 30 min at 4 °C in the dark. For intracellular staining of GZMB in CD8^+^ T cells, the cells were stimulated with leukocyte activation cocktail (BD Biosciences, USA) for 6 h. Cells were fixed and permeabilized using the fixation/permeabilization kit (BD Biosciences, USA) for 20 min according to the manufacturer’s instructions, followed by staining with intracellular marker antibodies for 30 min at 4 °C. Data acquisition was performed using an LSR Fortessa flow cytometer (BD Biosciences, USA), and analyzed by FlowJo software (v10.8.1; TreeStar Inc., Ashland, OR, USA).

### Cell Cycle and Cell Apoptosis Analysis

Cell cycle distribution and apoptosis were evaluated using a Cell Cycle Analysis Kit and an Annexin V–FITC Apoptosis Detection Kit (Beyotime), respectively. Flow cytometric acquisition was performed on an LSR Fortessa (BD Biosciences), and data were analyzed using FlowJo software (Tree Star).

### Hematoxylin-Eosin (H&E) and Immunofluorescence Staining

Tumor tissues from fresh patient tumor samples or cultured PDOs were fixed in 4% PFA and dehydrated through a graded alcohol series before embedding in paraffin for section with 10 μm thickness. Tissue sections were stained with hematoxylin and eosin (H&E). For immunofluorescence staining, the tissue sections were incubated with specific primary antibodies, followed by secondary antibodies conjugated to fluorescent probes. All images were obtained by a PathScan combi digital pathology scanner (Excilone, France).

## Supplementary Material















## Data Availability

All data supporting the findings of this study are presented in the Article and its Information files. Raw mass spectrometry files associated with AS-MS screening and metabolomics profiling are available via Figshare at (access for reviewers: https://figshare.com/s/879442d2e5d9f57add81).

## Notes

Additional experimental data are listed in Figure S1–11; characterization of all compounds was described in Note S1 (PDF)
Table S1. 13 TCM herbs; 30 crude extracts (XLSX)
Table S2. Virtual screening hits from an ER-focused compound library (XLSX)
Table S3. AS-MS screening (XLSX)
Table S4. Metabolomics profiling (XLSX)
Table S5. The inhibitory effect of ER-15 against other GPCRs evaluated by Tango assay; Table S6. Potency and efficacy of ER-15 and ZM241385 antagonism related to Figure 4; Table S7. Information of the patients (PDF)
Transparent Peer Review report available (PDF)
