# Supplementary material for: Target-Centric Multiplexed Screening of an Herbal Extract Identifies a Novel Dual A2A/A2B Receptor Antagonist for Cancer Immunotherapy
Source: ACS Cent Sci. 2026 Mar 13;12(3):358–74. doi: 10.1021/acscentsci.5c01843 (PMC13022719; doi:10.1021/acscentsci.5c01843)
Supplement: Supplementary file 1 [file oc5c01843_si_001.pdf]

Supporting Information for Publication

Target-centric multiplexed screening of a herbal extract identifies a novel dual A_{2A}/A_{2B} receptor antagonist for cancer immunotherapy

Hongyue Liu[1,2,3‡], Xinyu Yang[4‡], Jingyi Xu[1,2,3‡], Yuefei Wang[5], Zichen Zhao[1,2], Ke Quan[4], Aoqi Gao[4], Yang Wang[6], Jingbo Wu[6], Fei Li[1], Zhaoyu Zhang[1,2,3], Yuanyuan Ma[1,2,3], Yuan Weng[4], Ying Chen[4], Liping Sun[1], Gaojie Song[4], Yibing, Shan[1,2], Xin Chai[5]*, Bingjie Zhang[1]*, Weiqiang Lu[4,7,8]*, Wenqing Shui[1,2]*

[1]iHuman Institute, ShanghaiTech University, Shanghai 201210, China

[2]School of Life Science and Technology, ShanghaiTech University, Shanghai 201210, China

[3]University of Chinese Academy of Sciences, Beijing 100049, China

[4]Shanghai Key Laboratory of Regulatory Biology, Institute of Biomedical Sciences and School of Life Sciences, East China Normal University, Shanghai 200241, China

[5]Tianjin University of Traditional Chinese Medicine, Tianjin 301617, China

[6]Department of Urology and Department of Pathology, The Fifth People's Hospital of Shanghai, Fudan University, Shanghai 201100, China

[7]Hainan Academy of Medical Sciences, Hainan Medical University, Haikou, Hainan, China; Engineering Research Center of Tropical Medicine Innovation and Transformation of Ministry of Education, Hainan Provincial Key Laboratory for Research and Development of Tropical Herbs and Haikou Key Laboratory of Li Nationality Medicine, School of Pharmacy, Hainan Medical University, Haikou 571199, China

[8]State Key Laboratory of Innovative Immunotherapy, School of Pharmaceutical Sciences, Shanghai Jiao Tong University, Shanghai, China

‡Equal contribution

*To whom correspondence should be addressed:

Wenqing Shui Email: shuiwq@shanghaitech.edu.cn

Weiqiang Lu Email: wqlu@bio.ecnu.edu.cn

Bingjie Zhang Email: zhangbj@shanghaitech.edu.cn

Xin Chai: chaix0622@tjutcm.edu.cn

Table of Content

Figure S1. Bioactivity screening of herbal extracts with cAMP accumulation assays. 3

Figure S2. Docking analysis of two known $A_{2A}R$ ligands in the receptor structure......4

Figure S3. Validation of the AS-MS screening workflow. 5

Figure S4. Additional cAMP accumulation assay results of ER-15............................6

Figure S5. Activity measurement of hit compounds yielded by different screening approaches. 7

Figure S6. Binding conformation of ER-15 generated by MD simulations 8

Figure S7. Cell surface expression levels of $A_{2A}R$ and $A_{2B}R$ mutants relative to the WT measured by flow cytometry...9

Figure S8. ER-15 bound-$A_{2B}R$ structure predicted by AF3 (A) and residue conservation analysis (B)...10

Figure S9. Cell viability assays of ER-15 and ER extracts.......................................11

Figure S10. Representative MC38 tumors and flow cytometry gating strategy. 12

Figure S11. Representative histology of CRC biopsy and flow cytometry gating strategy..14

Note S1. NMR spectra of ER-15 and other ER- derived compounds.......................15

Tables S1-S7 *are provided as separate files.*

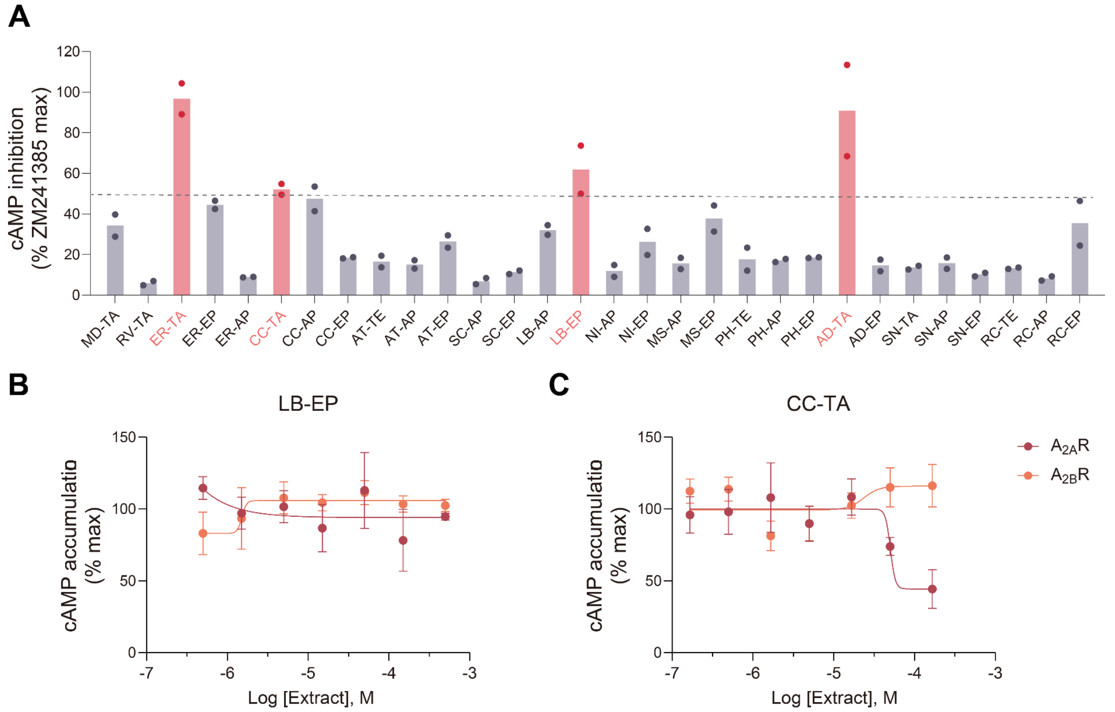

Figure S1. Bioactivity screening of herbal extracts with cAMP accumulation assays.

(**A**) In single-dose cAMP assays on 30 crude extracts, four bioactive extracts showed an antagonistic activity on $A_{2A}R$ (> 50% E_{max} relative to the reference compound), which were ER-TA (total alkaloids of *Evodia rutaecarpa),* CC-TA (total alkaloids of *Coptis chinensis),* LB-EP (ethyl acetate phase of *Lycium barbarum),* and AD-TA (total alkaloids of *Aristolochia debilis*). Data are shown as means from two independent experiments. Full names of other tested herbs are listed in **Table S1**. (**B**) Dose response curves of cAMP accumulation assays in $A_{2A}R$-transfected HEK293 cells treated with CC-TA and LB-EP. The estimated concentrations of herbal extracts were calculated from weight, assuming an average molecular weight of 500 Da for small molecule constituents. Data are shown as means ± SEM from three independent experiments.

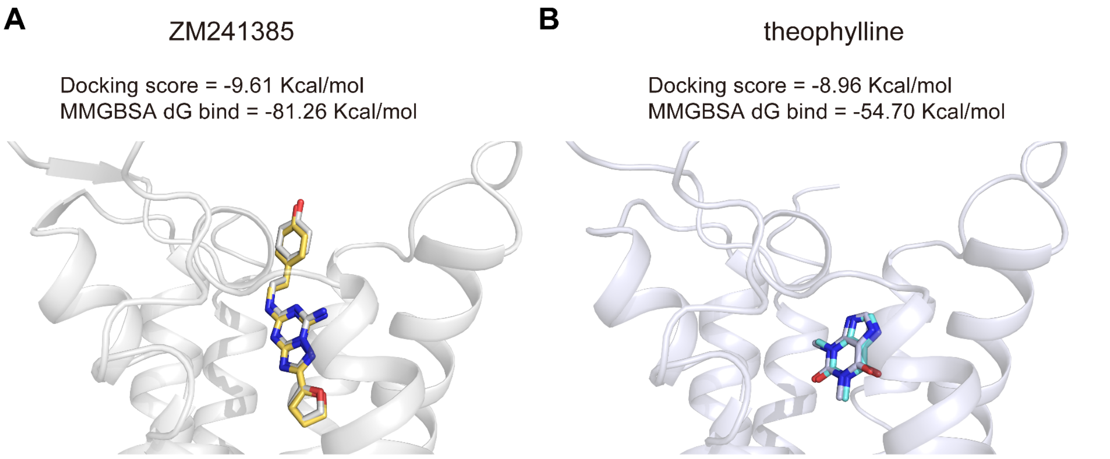

Figure S2. Docking analysis of two known A_{2A}R ligands in the receptor structure. Docking pose of ZM241385 (yellow) overlaid with the compound in the original structure (PDB: 4EIY, left) and that of theophylline (blue) overlaid with the compound in the original structure (PDB: 5MZJ, right). White, the original crystal structure of inactive A_{2A}R. Docking scores and MM-GBSA dG binding energy are shown above the graph.

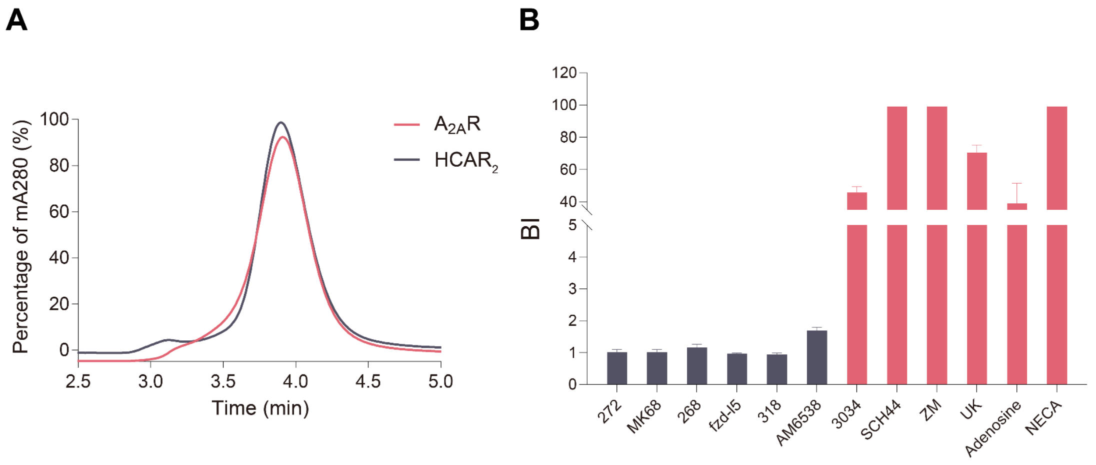

Figure S3. Validation of the AS-MS screening workflow.

(**A**) Analytical size-exclusion chromatography (aSEC) profiles of purified $A_{2A}R$ and $HCAR_2$ protein. (**B**) AS-MS screening of a compound mixture containing six known $A_{2A}R$ ligands (red bars) and six unrelated compounds (grey bars). The binding index (BI) is defined as the ratio of the compound's MS intensity detected in the target *vs* control. Positive binders are defined by the following criteria: mean BI > 2, $P < 0.01$, n = 4.

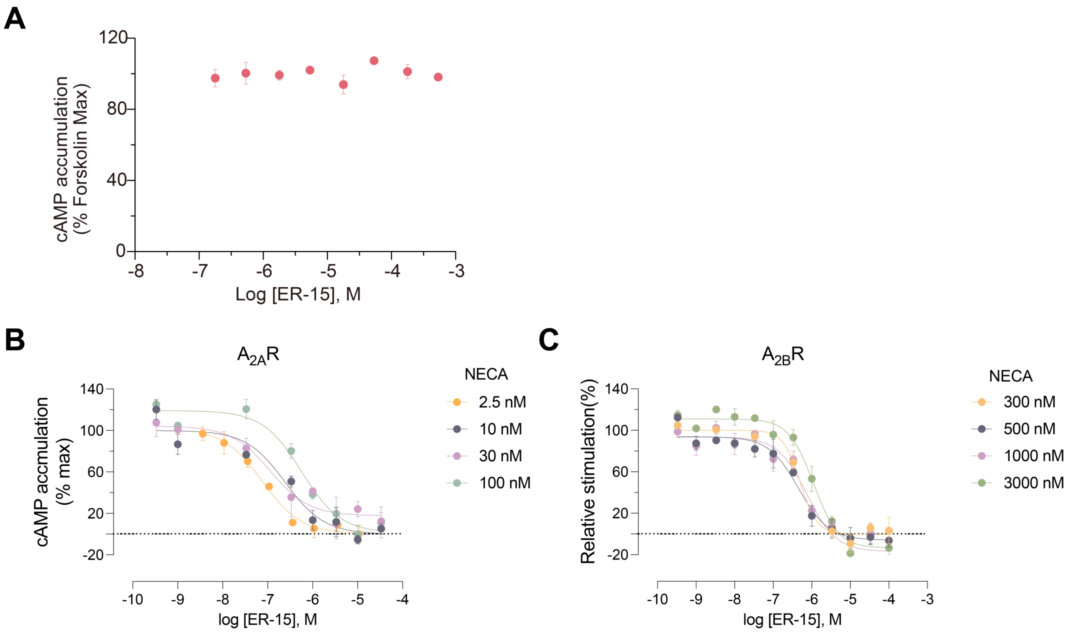

NECA conc	2.5 nM	10 nM	30 nM	100 nM
IC_{50} of ER-15 (nM)	76.2	246.0	113.8	569.8

NECA conc	300 nM	500 nM	1000 nM	3000 nM
IC_{50} of ER-15 (nM)	499.7	456.6	750.6	1110

Figure S4. Additional cAMP accumulation assay results of ER-15.

(**A**) cAMP accumulation assay on forskolin-stimulated HEK293 cells treated with ER-15. ER-15 showed no inhibition of forskolin-stimulated cAMP production in HEK293 cells. Cells were pretreated with forskolin (30 μM) for 15 min. Data are shown as means ± SEM from three independent experiments. (**B-C**) The dose−response curves of ER-15 against $A_{2A}R$ (**B**) and $A_{2B}R$ (**C**) under various levels of NECA concentration. Cells were pretreated with various levels of NECA concentration for 15 min. Data are shown as means ± SEM from three independent experiments. Mean IC_{50} values from experimental triplicate are shown in the tables.

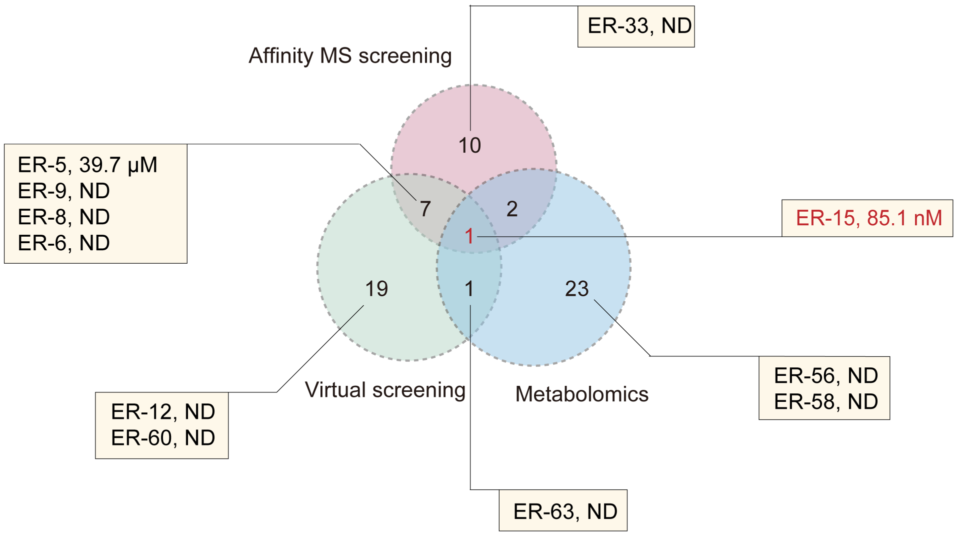

Figure S5. Activity measurement of hit compounds yielded by different screening approaches.

Selected hit compounds and their corresponding IC_{50} values determined by $A_{2A}R$-mediated cAMP accumulation assays are shown in the boxes. The only hit compound co-identified by all three methods was ER-15 (highlighted in red). ND, no antagonistic activity determined. Compound names and structures are provided in **Tables S3 and S4**.

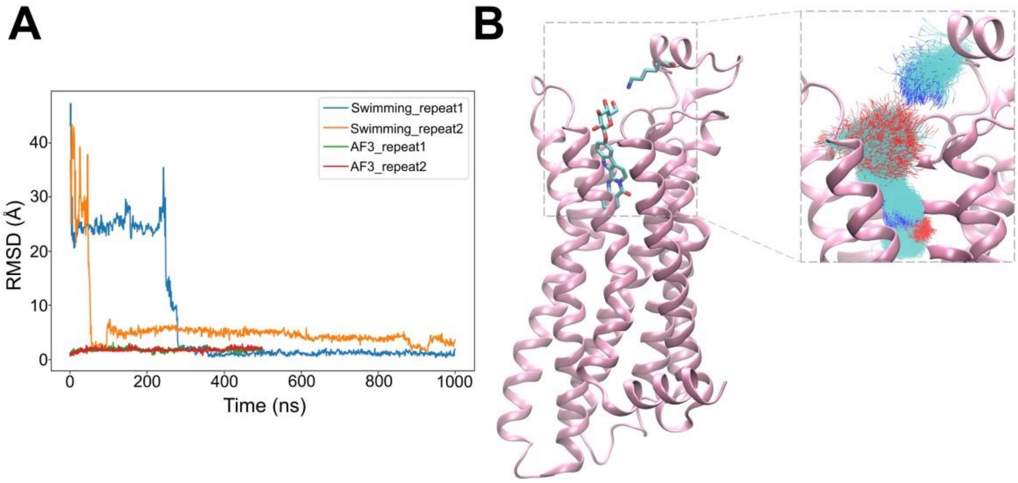

Figure S6. Binding conformation of ER-15 generated by MD simulations.

(**A**) The RMSD of the ER-15 molecule with respect to the binding pose predicted by AF3 in the two ligand-swimming simulations (1000 ns) and in the two simulations starting from the AF3 prediction. The conformationally flexible glucose moiety is not included in the RMSD calculation. (**B**) ER-15 snapshots from another all-atom MD simulation (500 ns) experiment starting from the AF3-predicted $A_{2A}R$ complex structure. The simulations confirm that the glucose moiety of ER-15 is in close and frequent interactions with K153 in ECL2.

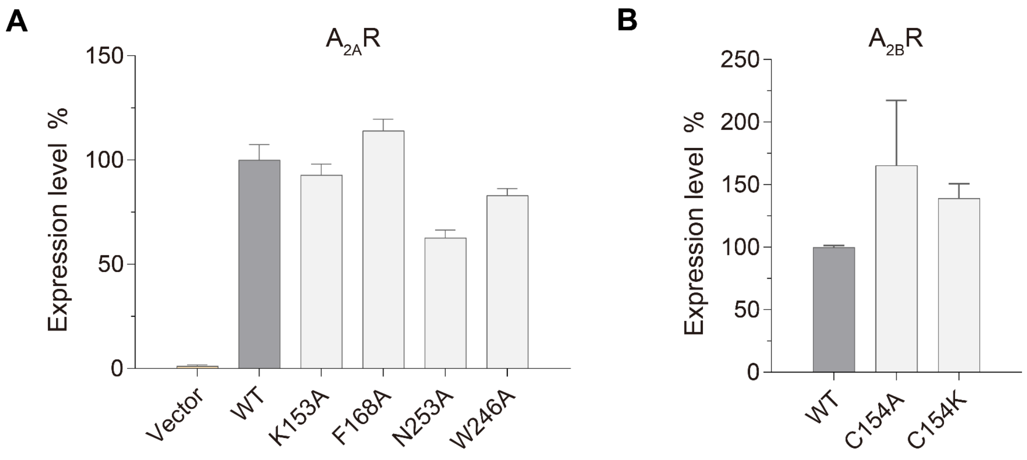

Figure S7. Cell surface expression levels of $A_{2A}R$ and $A_{2B}R$ mutants relative to the WT measured by flow cytometry.

Data are shown as means ± SEM from three independent experiments.

A

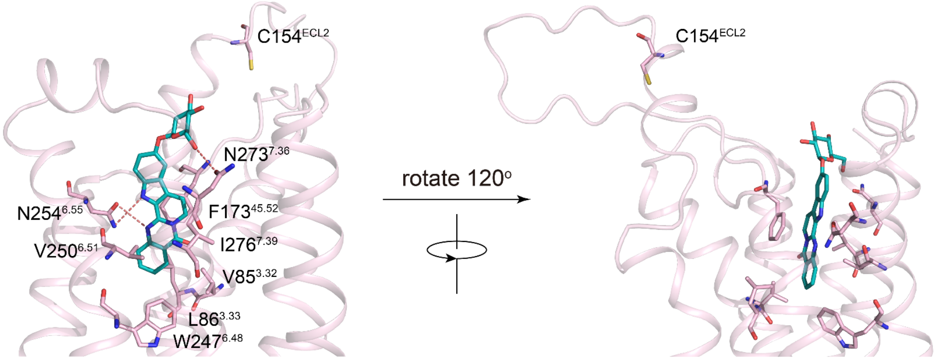

B

BW numbering	3.32	3.33	-	45.52	6.48	6.51	6.55
Number in $A_{2A}R$	84	85	153	168	246	249	253
A_1R	V	L	W	F	W	L	N
$A_{2A}R$	V	L	K	F	W	L	N
$A_{2B}R$	V	L	C	F	W	V	N
A_3R	L	L	R	F	W	L	N

Figure S8. ER-15 bound-$A_{2B}R$ structure predicted by AF3 (A) and residue conservation analysis (B).

(**A**) Hydrogen bonds between ER-15 and specific residues in the pocket of the predicted structure are indicated by dash lines. $C154^{ECL2}$ in $A_{2B}R$ is distant from the ER-15 binding pocket. (**B**) Conservation analysis of $A_{2A}R$ residues predicted to interact with ER-15. $K153^{ECL2}$ ($A_{2A}R$) is not conserved in the adenosine receptor subfamily, while other residues are all conserved.

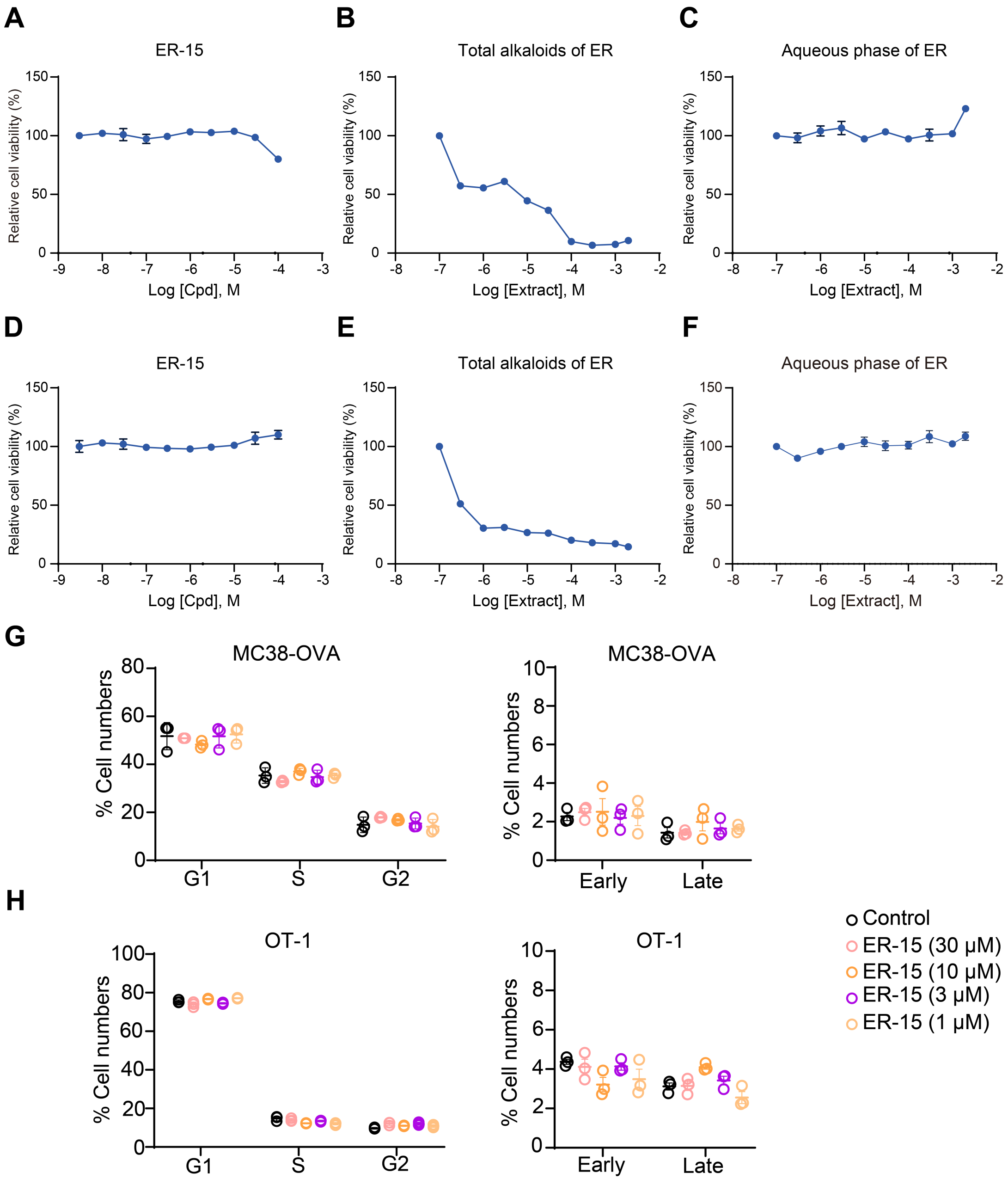

Figure S9. Cell viability assays of ER-15 and ER extracts.

(**A-C**) Cell viability assays of MC38-OVA cells treated with ER-15, the total alkaloids of ER, or aqueous phase of ER for 48 hours. (**D-F**) Cell viability assays of OT-I CTL cells treated with ER-15, total alkaloids of ER or aqueous phase of ER for 48 hours. (**G-H**) Cell cycle and cell apoptosis analysis of MC38-OVA (**G**) and OT-1 (**H**) cells upon ER-15 treatment (1, 3, 10, 30 μM, for 48 h).

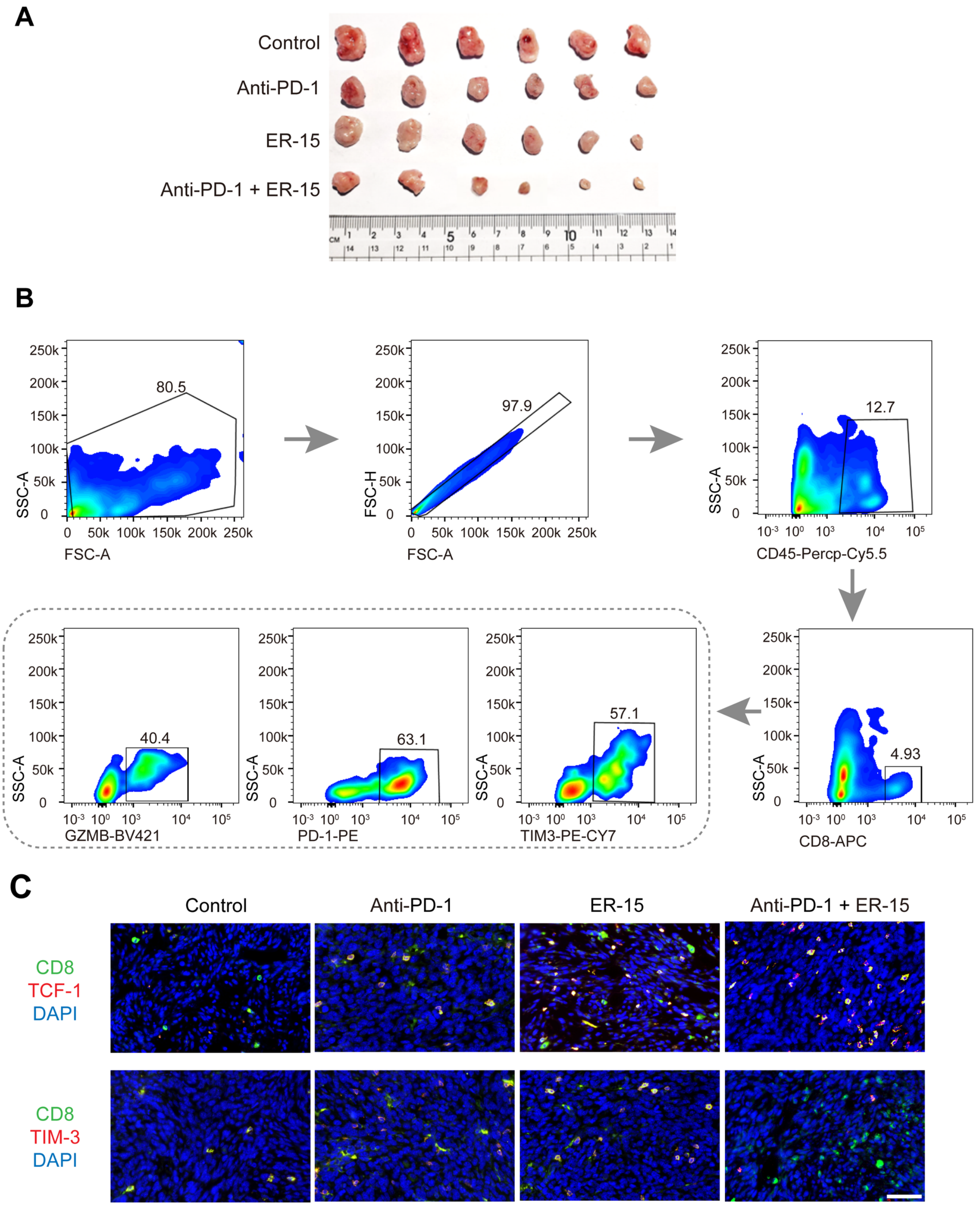

Figure S10. Representative MC38 tumors and flow cytometry gating strategy. (**A**) Representative photograph of freshly isolated MC38 tumors from C57BL/6 mice. C57BL/6 mice were inoculated subcutaneously with 1×10^6 MC38 cells and were injected intraperitoneally with vehicle, 50 mg/kg ER-15, 2.5 mg/kg anti-PD-1 antibody or their combinations starting at day 7 after inoculation. (**B**) Gating strategies for mouse lymphocytes in MC38 model. $CD8^+$ T cells gated on $CD45^+$ cells; $GZMB^+$ cells, $PD\text{-}1^+$ cells and $TIM\text{-}3^+$ cells gated on $CD45^+CD8^+$ cells. (**C**) Representative images of

immunofluorescence analysis of MC38 tumor sections stained for progenitor-exhausted $CD8^+$ T-cells (Tpex, $CD8^+TCF\text{-}1^+$) and terminally exhausted $CD8^+$ T-cells (Ttex, $CD8^+TIM\text{-}3^+$) following treatment with vehicle, anti-PD-1 antibody, ER-15 or their combination as in Figure 5F. Scale bar, 50 μm.

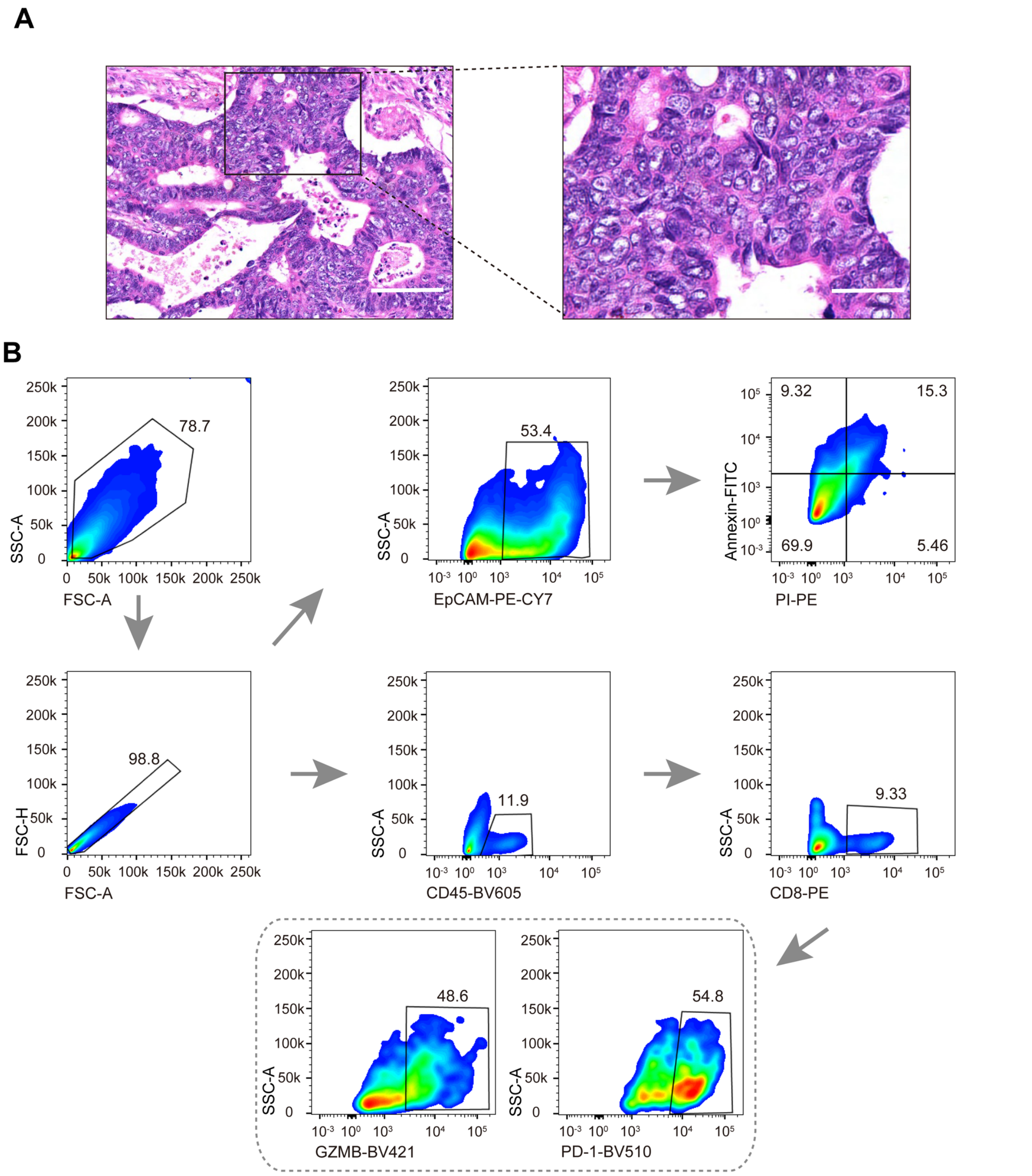

Figure S11. Representative histology of CRC biopsy and flow cytometry gating strategy.

(A) Representative H&E staining of freshly obtained human colorectal (CRC) biopsy (scale bars, 100 μm (left); 50 μm (right)). (B) Gating strategies for human tumor cell and lymphocytes in the PDO model. $CD8^+$ T cells gated on $CD45^+$ cells; $GZMB^+$ cells, $PD\text{-}1^+$ cells gated on $CD45^+CD8^+$ cells; apoptotic cells gated on $EpCAM^+$ tumor cells.

Note S1. NMR spectra of ER-15 and other ER- derived compounds.

Rutaecarpine-10-*O*-*β*-D-glucopyranoside (ER-15)

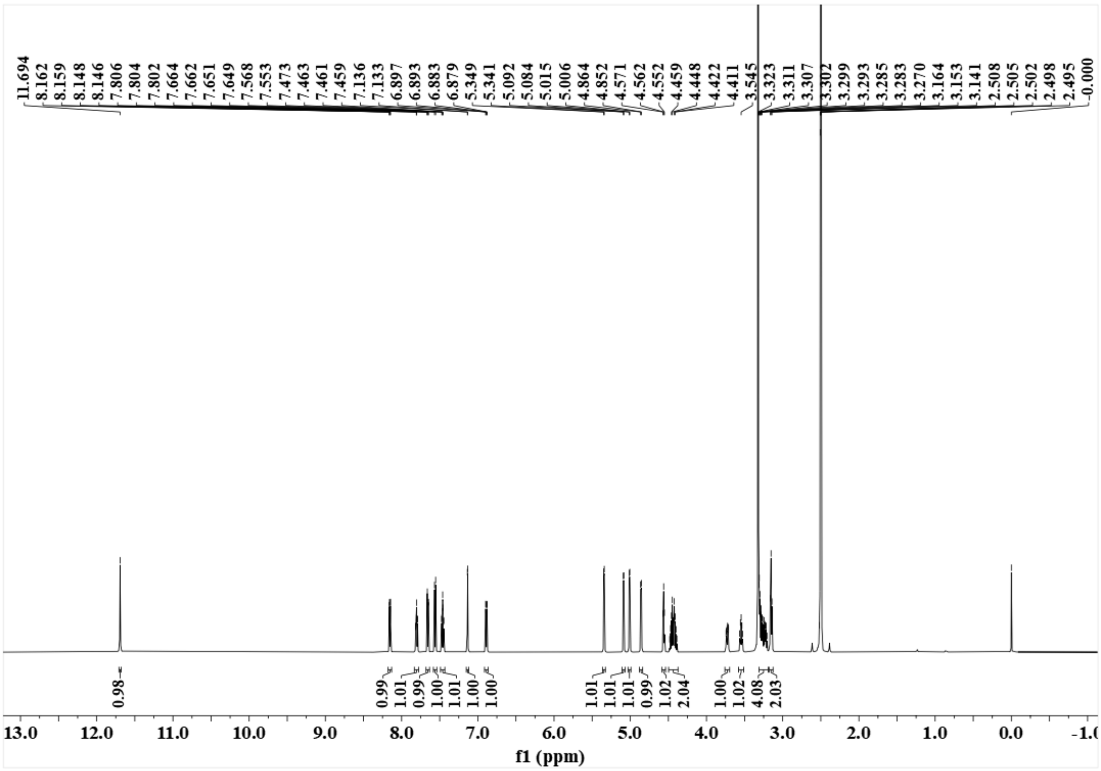

^{1}H-NMR spectrum of rutaecarpine-10-*O*-*β*-D-glucopyranoside

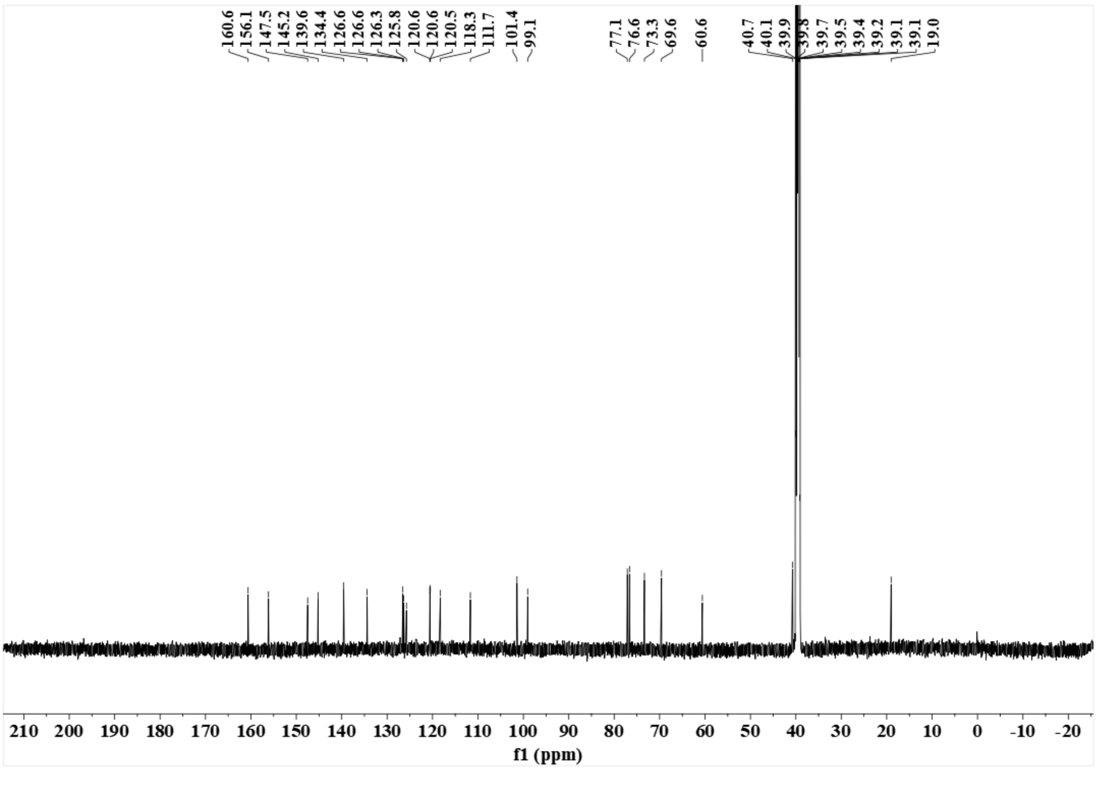

^{13}C-NMR spectrum of rutaecarpine-10-*O*-*β*-D-glucopyranoside

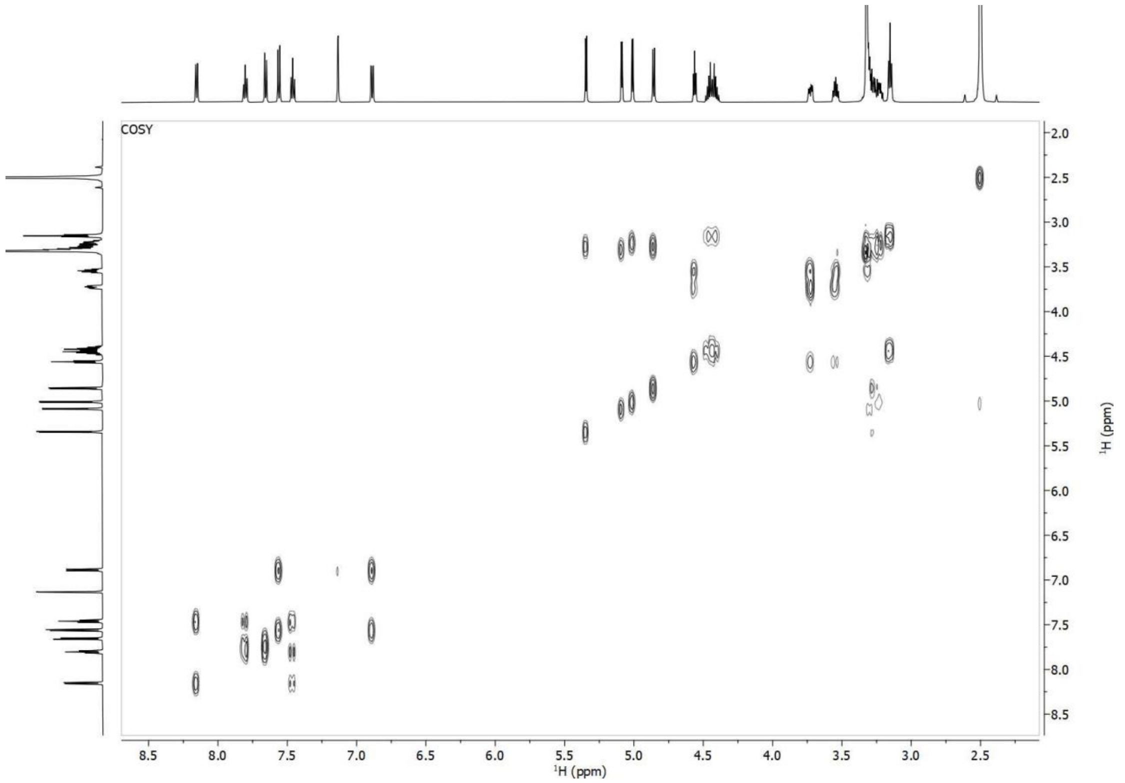

^{1}H-^{1}H COSY-NMR spectrum of rutaecarpine-10-*O*-*β*-D-glucopyranoside

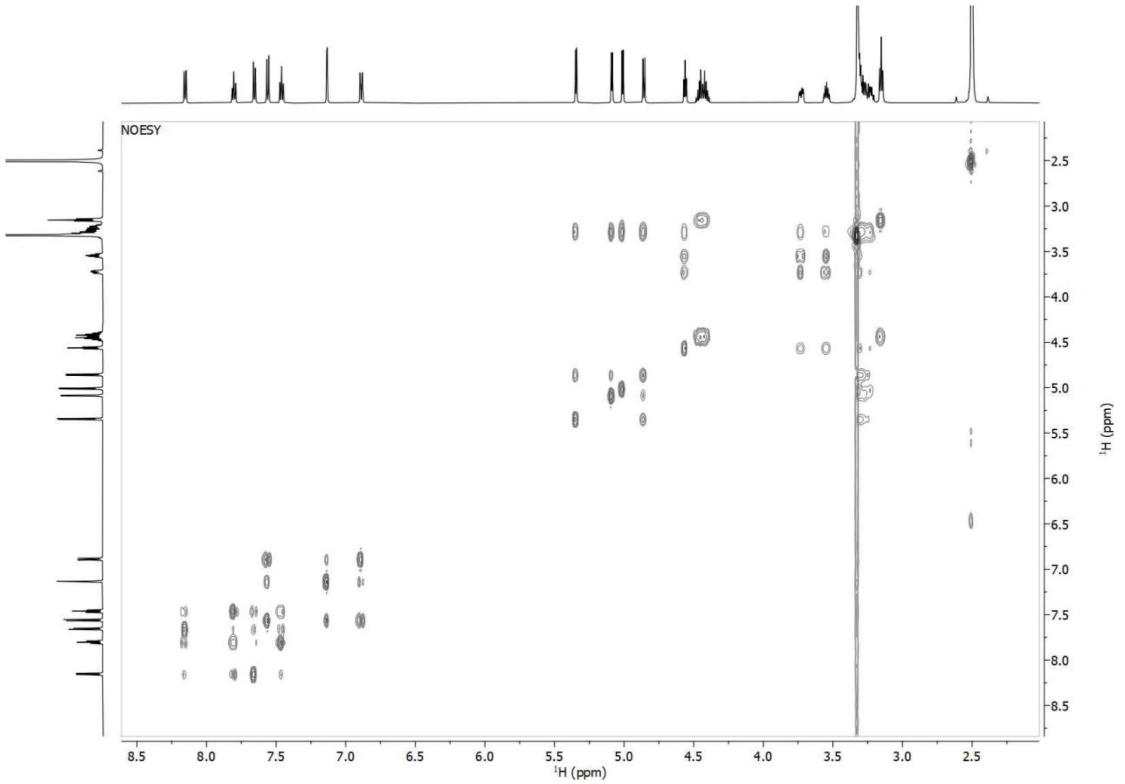

NOESY-NMR spectrum of rutaecarpine-10-*O*-*β*-D-glucopyranoside

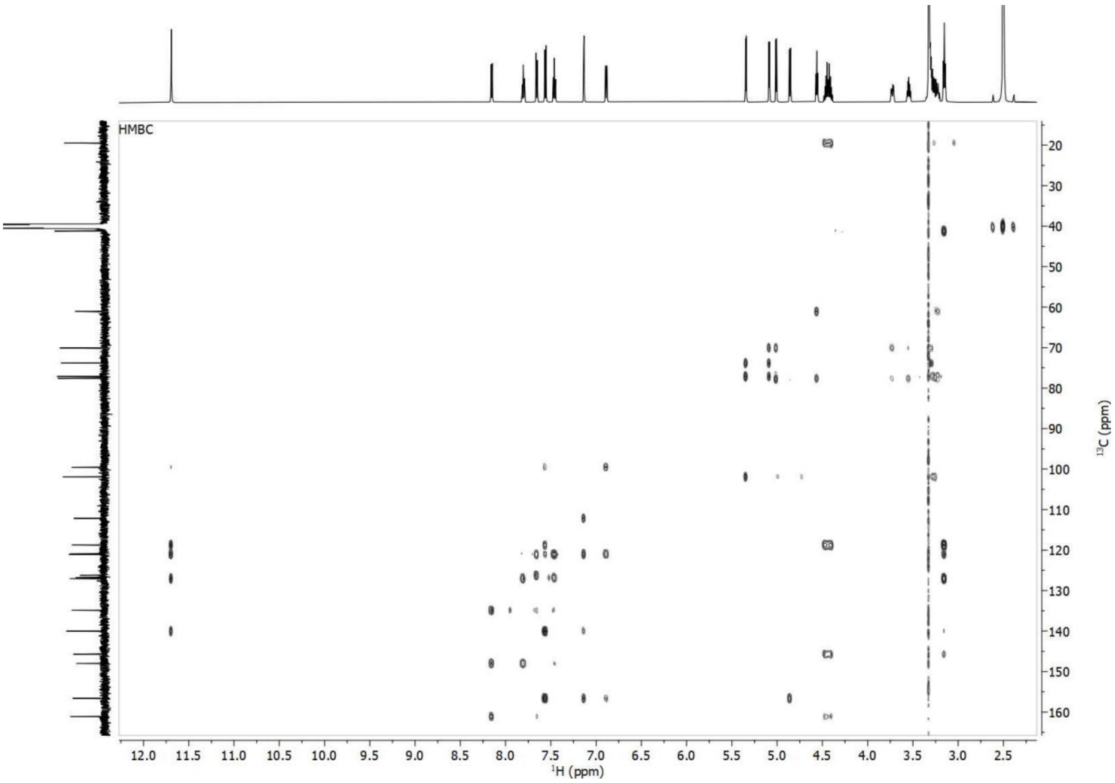

HMBC-NMR spectrum of Rutaecarpine-10-*O*-*β*-D-glucopyranoside

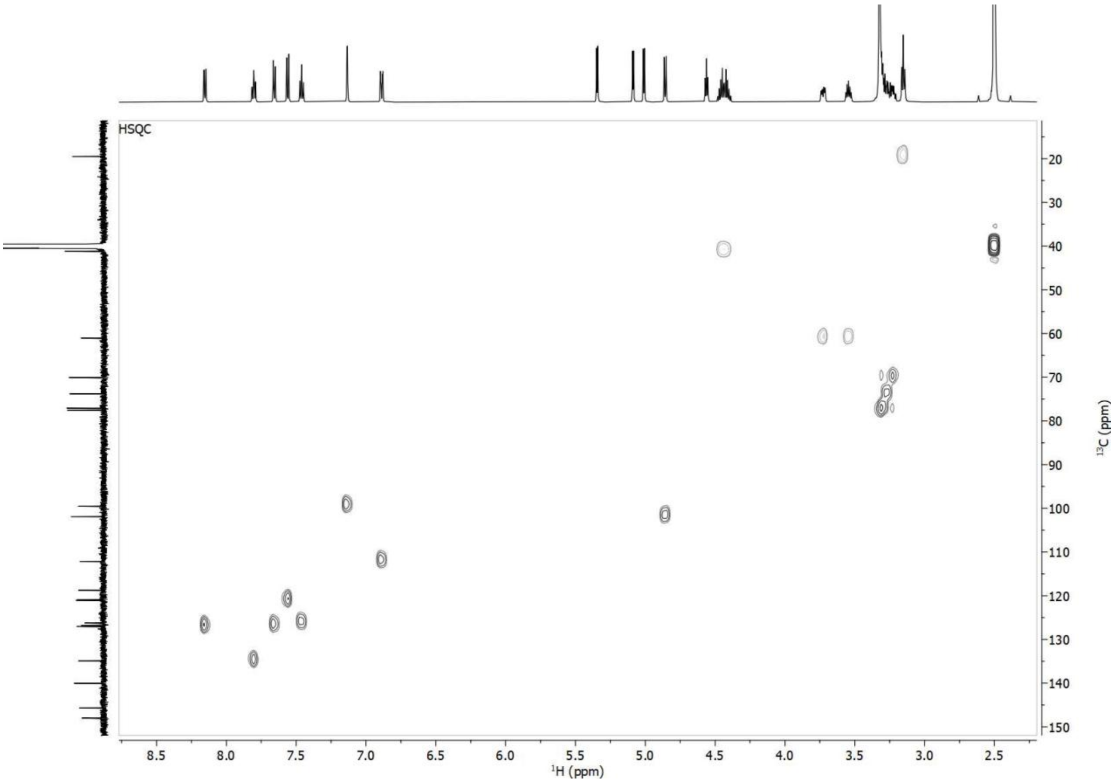

HSQC-NMR spectrum of rutaecarpine-10-*O*-*β*-D-glucopyranoside

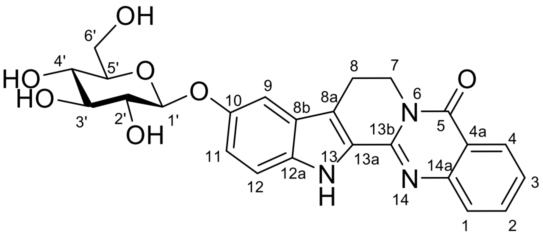

Rutaecarpine-10-*O*-*β*-D-glucopyranoside

^{1}H NMR (800 MHz, DMSO-d_6) δ 11.69 (1H, s, H-13), 8.15 (1H, dd, J = 8.0, 1.6 Hz, H-4), 7.80 (1H, ddd, J = 8.4, 7.1, 1.6 Hz, H-2), 7.66 (1H, dd, J = 8.3, 1.1 Hz, H-1), 7.56 (1H, d, J = 8.7 Hz, H-12), 7.46 (1H, ddd, J = 8.1, 7.1, 1.2 Hz, H-3), 7.13 (1H, d, J = 2.1 Hz, H-9), 6.89 (1H, dd, J = 8.7, 2.2 Hz, H-11), 5.35 (1H, d, J = 5.1 Hz, OH-2'), 5.09 (1H, d, J = 4.6 Hz, OH-4'), 5.01 (1H, d, J = 5.3 Hz, OH-3'), 4.86 (1H, d, J = 7.4 Hz, H-1'), 4.56 (1H, t, J = 5.8 Hz, OH-6'), 4.50 – 4.37 (2H, m, H-7), 3.73 (1H, ddd, J = 11.9, 5.4, 2.1 Hz, H-6'a), 3.54 (1H, dt, J = 11.7, 5.8 Hz, H-6'b), 3.31 – 3.20 (m, 4H, H-2', H-3', H-4' H-5'), 3.15 (2H, t, J = 6.9 Hz, H-8). ^{13}C NMR (150 MHz, DMSO-d_6) δ 160.6 (C-5), 156.1 (C-10), 147.5 (C-14a), 145.2 (C-13b), 139.6 (C-12a), 134.4 (C-2), 126.6 (C-4), 126.6 (C-1), 126.3 (C-13a), 125.8 (C-3), 120.6 (C-8b), 120.6 (C-4a), 120.5 (C-12), 118.3 (C-8a), 111.7 (C-11), 101.4 (C-1'), 99.1 (C-9), 77.1 (C-5'), 76.6 (C-3'), 73.3 (C-2'), 69.6 (C-4'), 60.6 (C-6'), 40.7 (C-7), 19.0 (C-8).

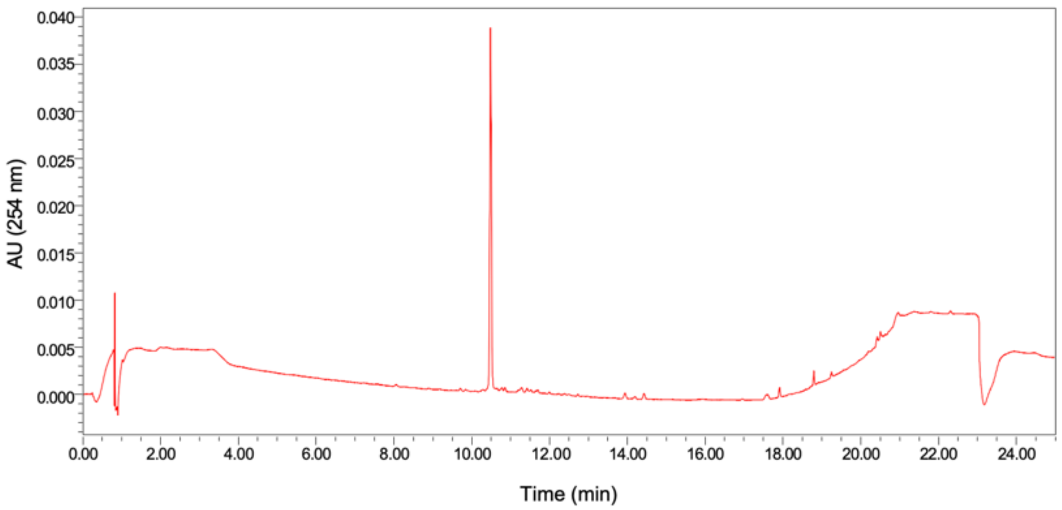

HPLC chromatogram of purified ER-15

7*β*-hydroxyrutaecarpine (ER-5)

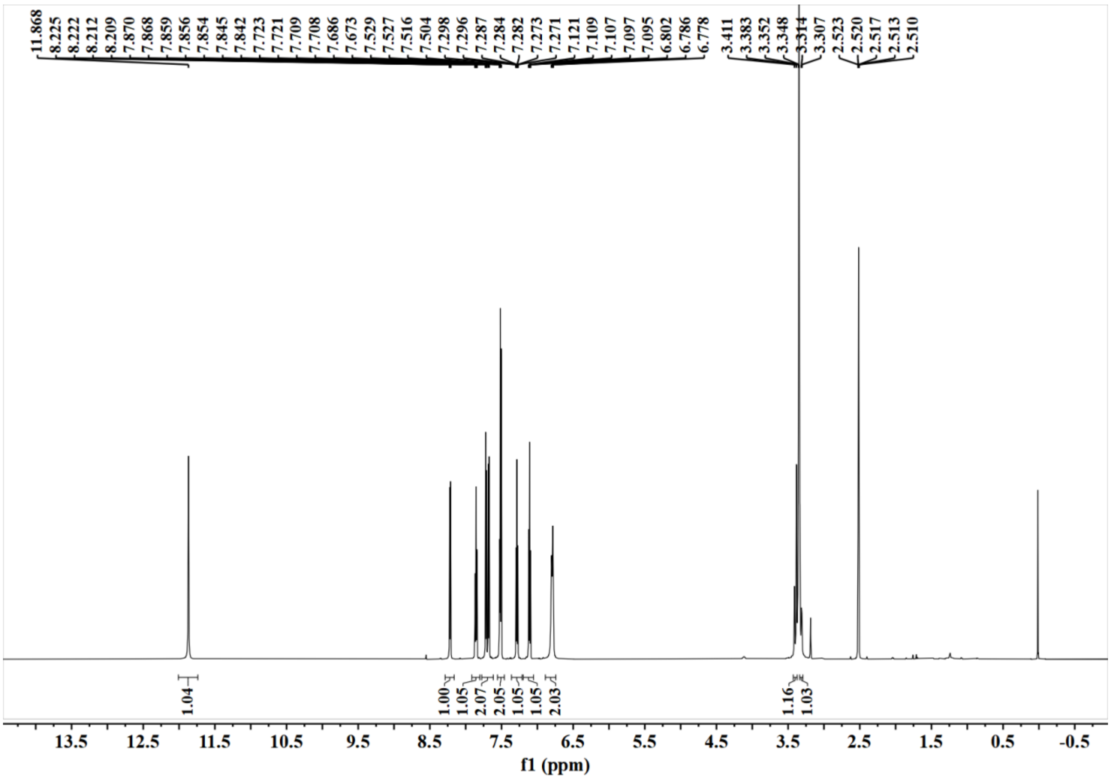

^{1}H-NMR spectrum of 7*β*-hydroxyrutaecarpine

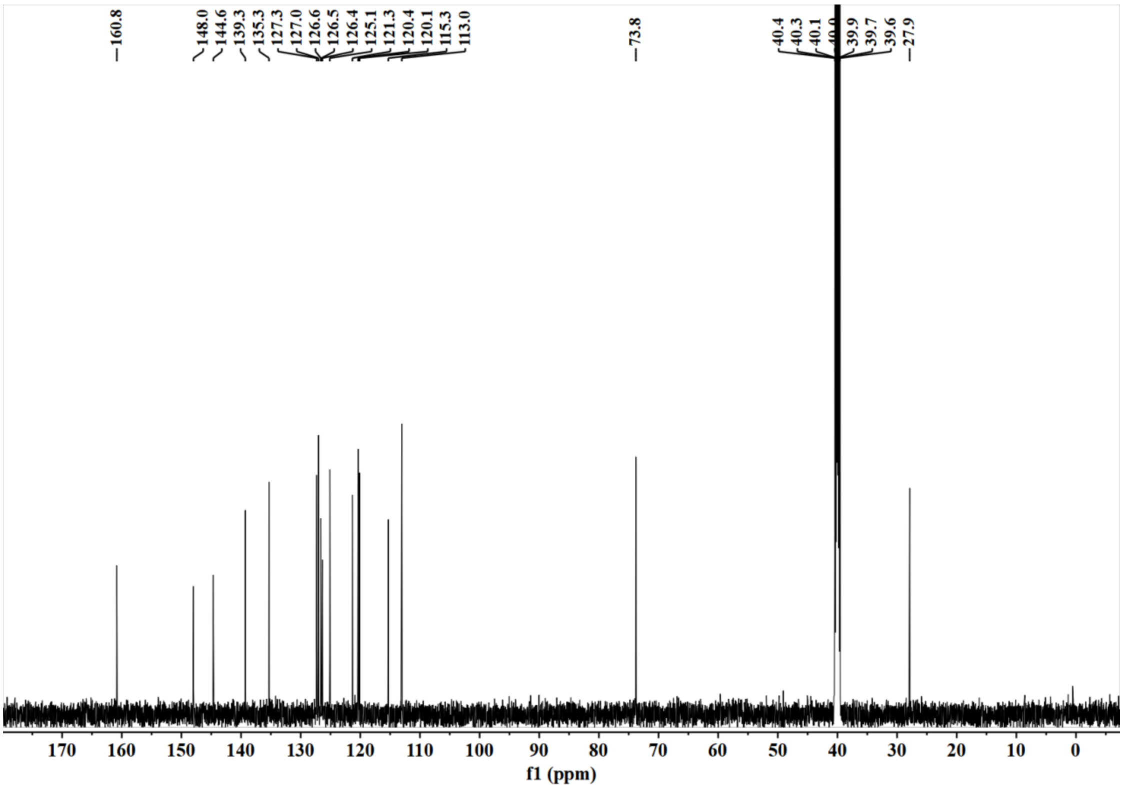

^{13}C-NMR spectrum of 7*β*-hydroxyrutaecarpine

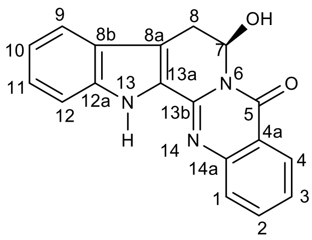

7*β*-hydroxyrutaecarpine

^{1}H NMR (600 MHz, DMSO-d_6) *δ*: 11.87 (1H, s, NH), 8.22 (1H, dd, *J* = 7.8, 1.8 Hz, H-4), 7.86 (1H, ddd, *J* =7.8, 7.8, 1.8 Hz, H-2), 7.72 (1H, d, *J* = 7.8 Hz, H-1), 7.68 (1H, d, *J* = 7.8 Hz, H-9), 7.52 (1H, ddd, *J* = 7.8, 7.8, 1.2 Hz, H-3), 7.51 (1H, d, *J* = 7.8 Hz, H-12), 7.28 (1H, ddd, *J* = 7.8, 7.8, 1.2 Hz, H-11), 7.11 (1H, ddd, *J* = 7.8, 7.8, 1.2 Hz, H-10), 6.80 (1H, brs, 7-OH), 6.79 (1H, brs, H-7_{eq}), 3.39 (1H, d, *J* = 16.8 Hz, H-8_{eq}), 3.34 (1H, dd, *J* = 16.8, 4.2 Hz, H-8_{ax}); ^{13}C NMR (150 MHz, DMSO-d_6) *δ*: 160.8 (C-5), 148.0 (C-14a), 144.6 (C-13b), 139.3 (C-12a), 135.3 (C-2), 127.3 (C-4), 127.0 (C-1), 126.6 (C-3), 126.5 (C-13a), 126.4 (C-8b), 125.1 (C-11), 121.3 (C-4a), 120.4 (C-9), 120.1 (C-10), 115.3 (C-8a), 113.0 (C-12), 73.8 (C-7), 27.9 (C-8).

1-hydroxyrutaecarpine (ER-6)

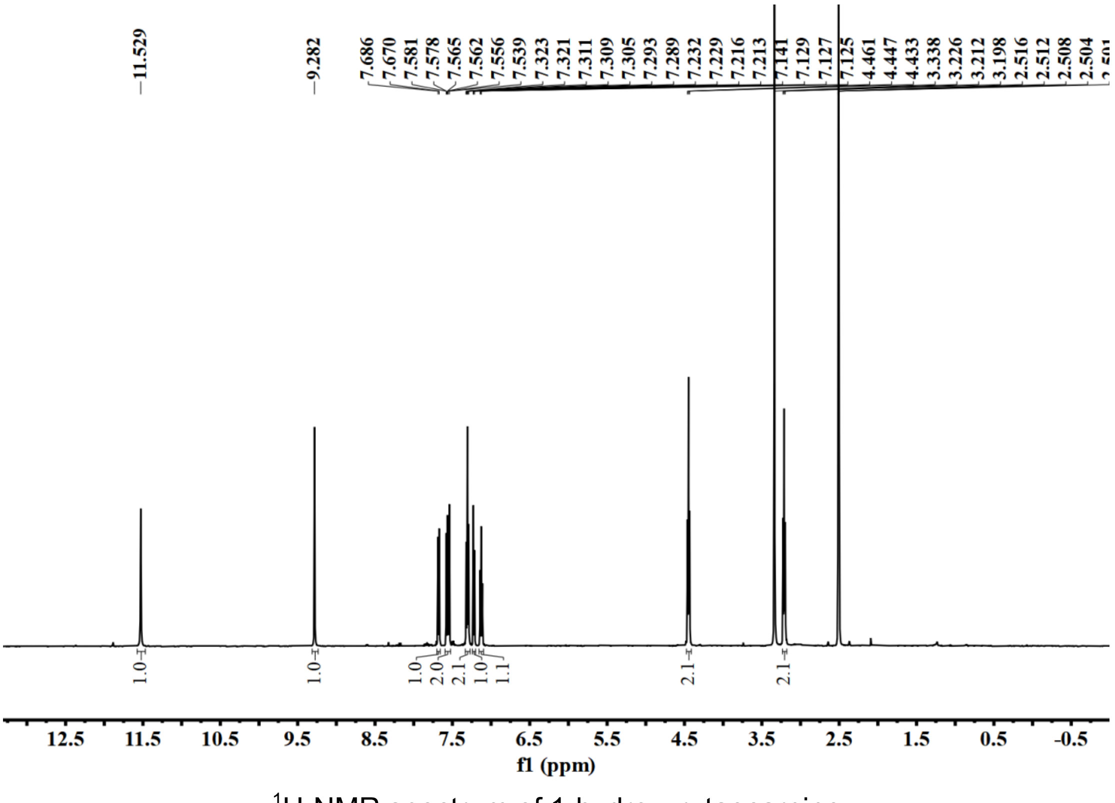

^{1}H-NMR spectrum of 1-hydroxyrutaecarpine

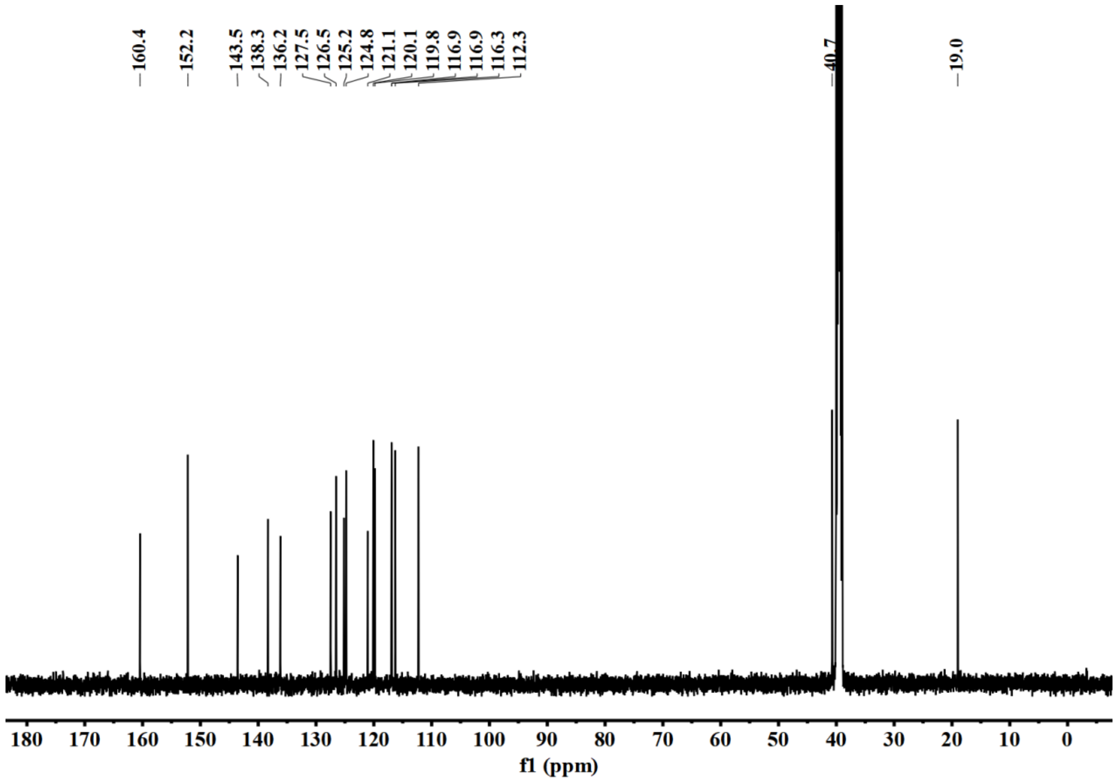

^{13}C-NMR spectrum of 1-hydroxyrutaecarpine

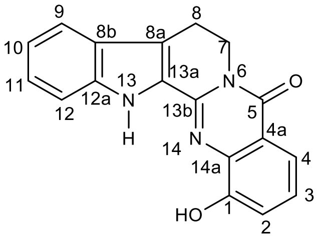

1-hydroxyrutaecarpine

^{1}H NMR (500 MHz, DMSO-d_6) δ: 11.53 (1H, brs, NH), 9.28 (1H, brs, 1-OH), 7.67 (1H, d, J = 8.0 Hz, H-9), 7.57 (1H, dd, J = 8.0, 1.5 Hz, H-4), 7.54 (1H, d, J = 8.5 Hz, H-12), 7.30 (1H, dd, J = 8.5, 8.0 Hz, H-11), 7.30 (1H, dd, J = 8.0, 8.0 Hz, H-3), 7.22 (1H, dd, J =8.0, 1.5 Hz, H-2), 7.13 (1H, dd, J = 8.0, 8.0 Hz, H-10), 4.45 (2H, t, J =7.0 Hz, H-7), 3.21 (2H, t, J = 7.0 Hz, H-8); ^{13}C NMR (125 MHz, DMSO-d_6) δ: 160.4 (C-5), 152.2 (C-1), 143.5 (C-13b), 138.3 (C-12a), 136.2 (C-14a), 127.5 (C-13a), 126.5 (C-3), 125.2 (C-8b), 124.8 (C-11), 121.1 (C-4a), 120.1 (C-9), 119.8 (C-10), 116.9 (C-8a), 116.9 (C-2), 116.3 (C-12), 112.3 (C-4), 40.7 (C-7), 19.0 (C-8).

Dehydroevodiamine (ER-60)

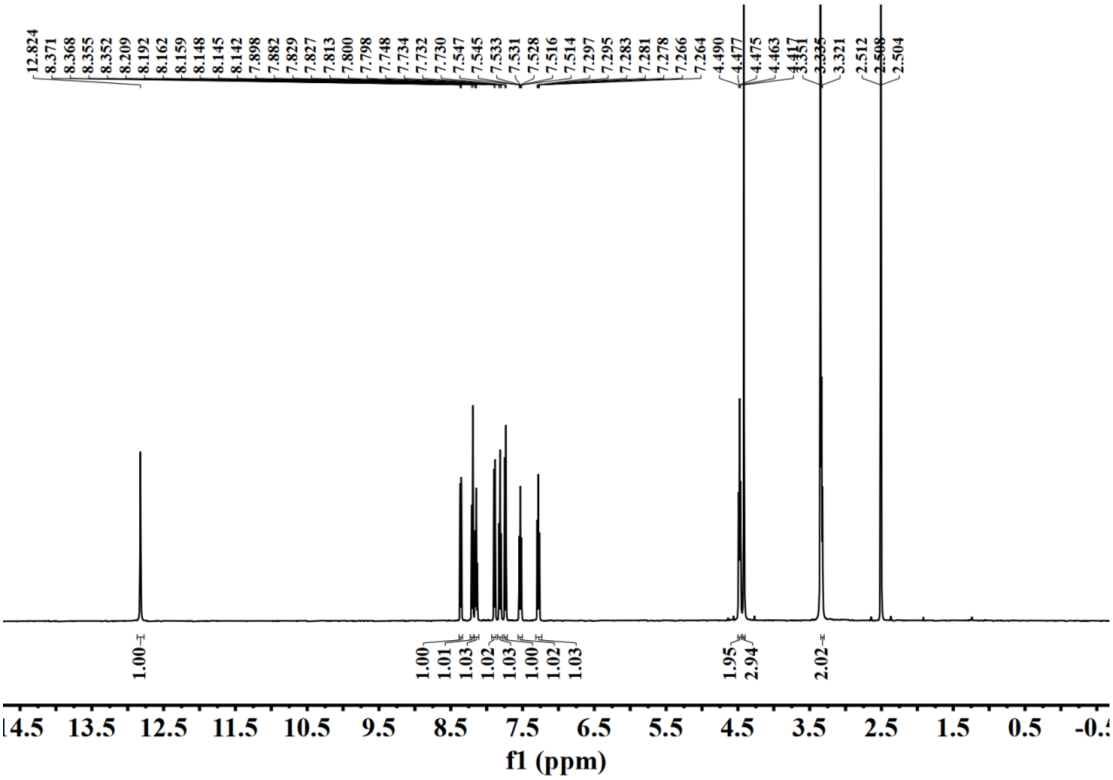

^{1}H-NMR spectrum of dehydroevodiamine

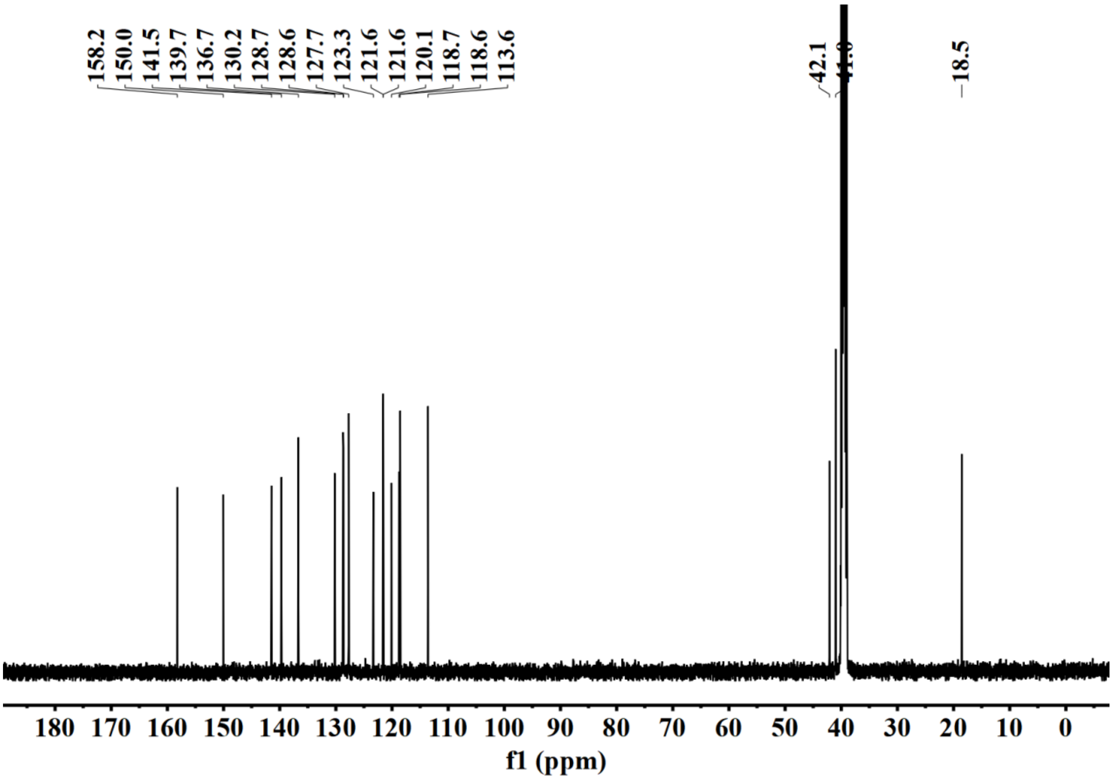

^{13}C-NMR spectrum of dehydroevodiamine

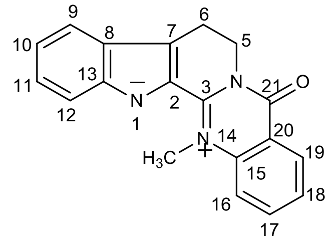

dehydroevodiamine

^{1}H NMR (500 MHz, DMSO-d_6) *δ*: 12.82 (1H, s, NH), 8.36 (1H, dd, *J* = 8.0, 1.5 Hz, H-19), 8.20 (1H, d, *J* = 8.5 Hz, H-16), 8.15 (1H, ddd, *J* = 8.5, 8.5, 1.5 Hz, H-17), 7.89 (1H, d, *J* = 8.0 Hz, H-9), 7.81 (1H, dd, *J* = 8.0, 8.0 Hz, H-18), 7.73 (1H, d, *J* = 8.0 Hz, H-12), 7.53 (1H, dd, *J* = 8.0, 8.0 Hz, H-11), 7.28 (1H, dd, *J* = 8.0, 8.0 Hz, H-10), 4.48 (2H, t, *J* = 7.0 Hz, H-5), 4.41 (3H, s, NCH_3), 3.33 (2H, t, *J* = 7.0 Hz, H-6); ^{13}C NMR (125 MHz, DMSO-d_6) *δ:* 158.2 (C-21), 150.0 (C-3), 141.5 (C-13), 139.7 (C-15), 136.7 (C-17), 130.2 (C-2), 128.7 (C-11), 128.6 (C-18), 127.7 (C-19), 123.3 (C-8), 121.6 (C-9), 121.6 (C-10), 120.1 (C-7), 118.7 (C-20), 118.6 (C-16), 113.6 (C-12), 42.1 (C-5), 41.0 (CH_3), 18.5 (C-6).

Rutaecarpine (ER-9)

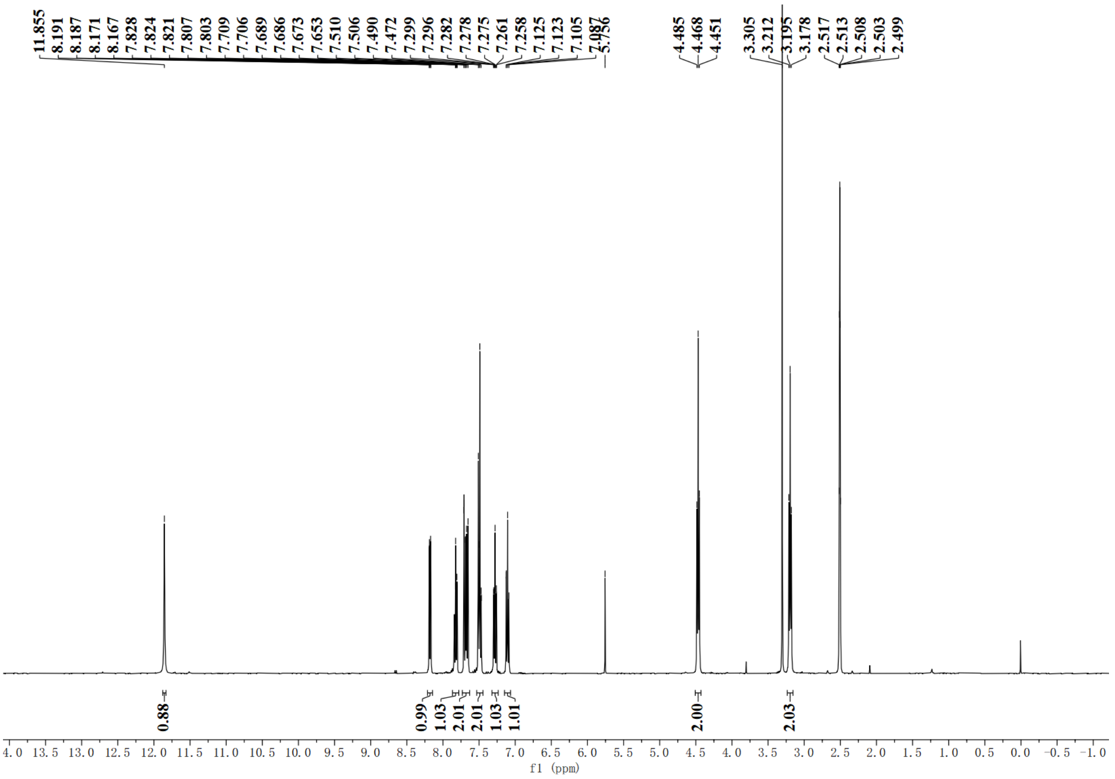

^{1}H-NMR spectrum of rutaecarpine

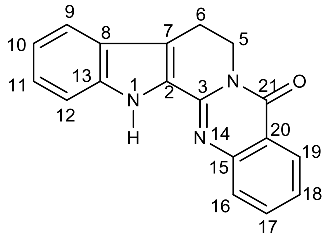

rutaecarpine^{1}H NMR (400 MHz, DMSO-d_6) δ: 11.86 (1H, s, NH), 8.18 (1H, dd, *J* = 7.8, 1.6 Hz, H-19), 7.82 (1H, ddd, *J* = 7.2, 7.2, 1.6 Hz, H-17), 7.70 (1H, d, *J* = 8.0 Hz, H-9), 7.66 (1H, d, *J* = 8.0 Hz, H-16), 7.50 (1H, d, *J* = 8.0 Hz, H-12), 7.48 (1H, ddd, *J* = 8.4, 8.0, 1.2 Hz, H-18), 7.27 (1H, ddd, *J* = 8.4, 8.0, 1.2 Hz, H-11), 7.11 (1H, ddd, *J* = 8.4, 8.0, 1.2 Hz, H-10), 4.47 (2H, t, *J* = 6.8 Hz, H-5), 3.20 (2H, t, *J* = 6.8 Hz, H-6).

Evodiamine (ER-12)

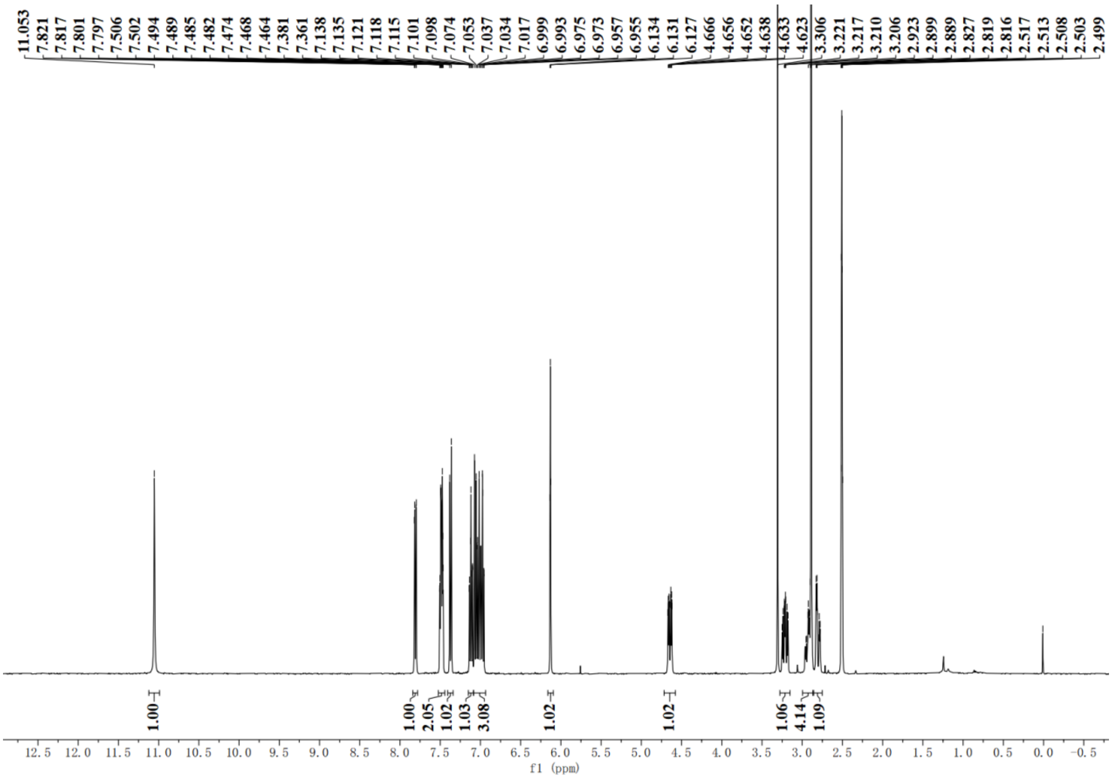

^{1}H-NMR spectrum of evodiamine

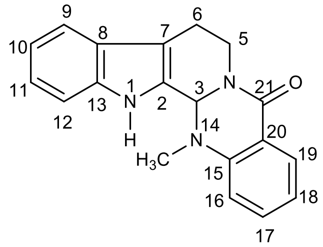

evodiamine

^{1}H NMR (400 MHz, DMSO-d_6) δ: 11.05 (1H, brs, NH), 7.81 (1H, dd, J = 8.0, 1.6 Hz, H-19), 7.49 (1H, m, H-17), 7.47 (1H, m, H-9), 7.37 (1H, d, J = 8.0 Hz, H-12), 7.12 (1H, ddd, J = 8.0, 8.0, 1.2 Hz, H-11), 7.06 (1H, d, J = 8.0 Hz, H-16), 7.02 (1H, dd, J = 8.0, 7.2 Hz, H-10), 6.98 (1H, dd, J = 8.0, 7.2 Hz, H-18), 6.13 (1H, s, H-3), 4.64 (1H, dd, J = 12.8, 5.6 Hz, H-5b), 3.21 (1H, dt, J = 12.4, 4.8 Hz, H-5a), 2.93 (1H, dd, J = 13.6, 4.4 Hz, H-6b), 2.89 (3H, s, CH_3), 2.80 (1H, dd, J = 13.6, 4.4 Hz, H-6a).

14-formyldihydrorutaecarpine (ER-33)

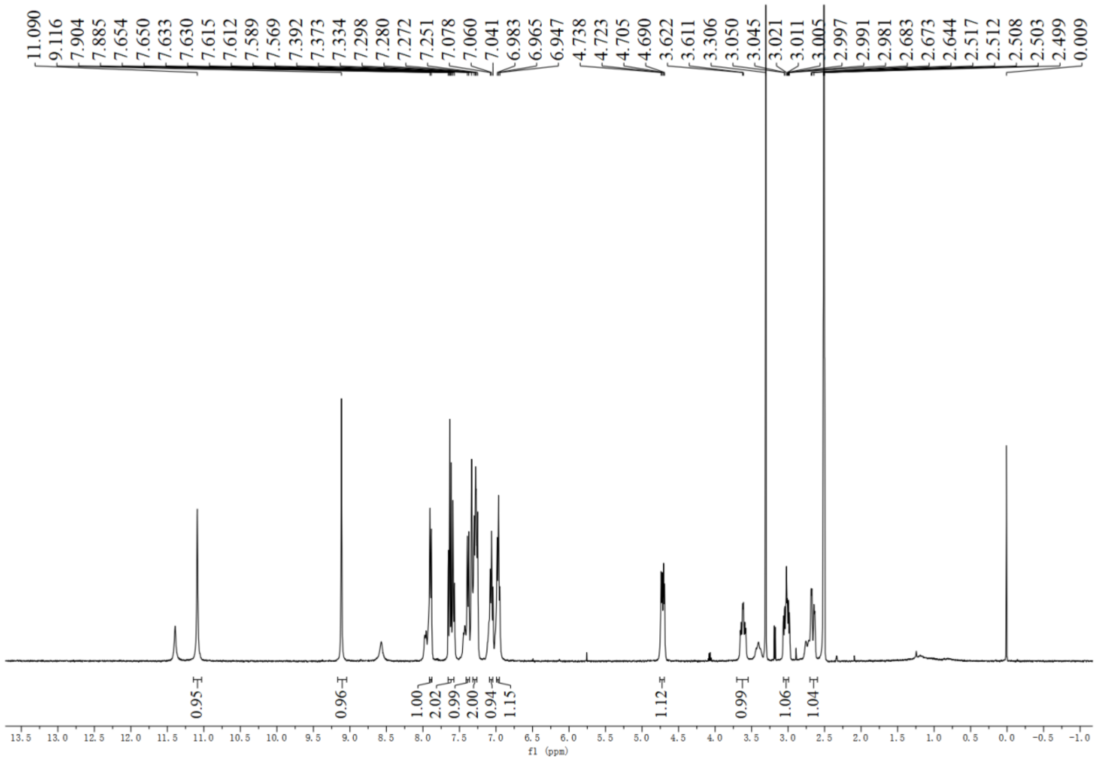

^{1}H-NMR spectrum of 14-formyldihydrorutaecarpine

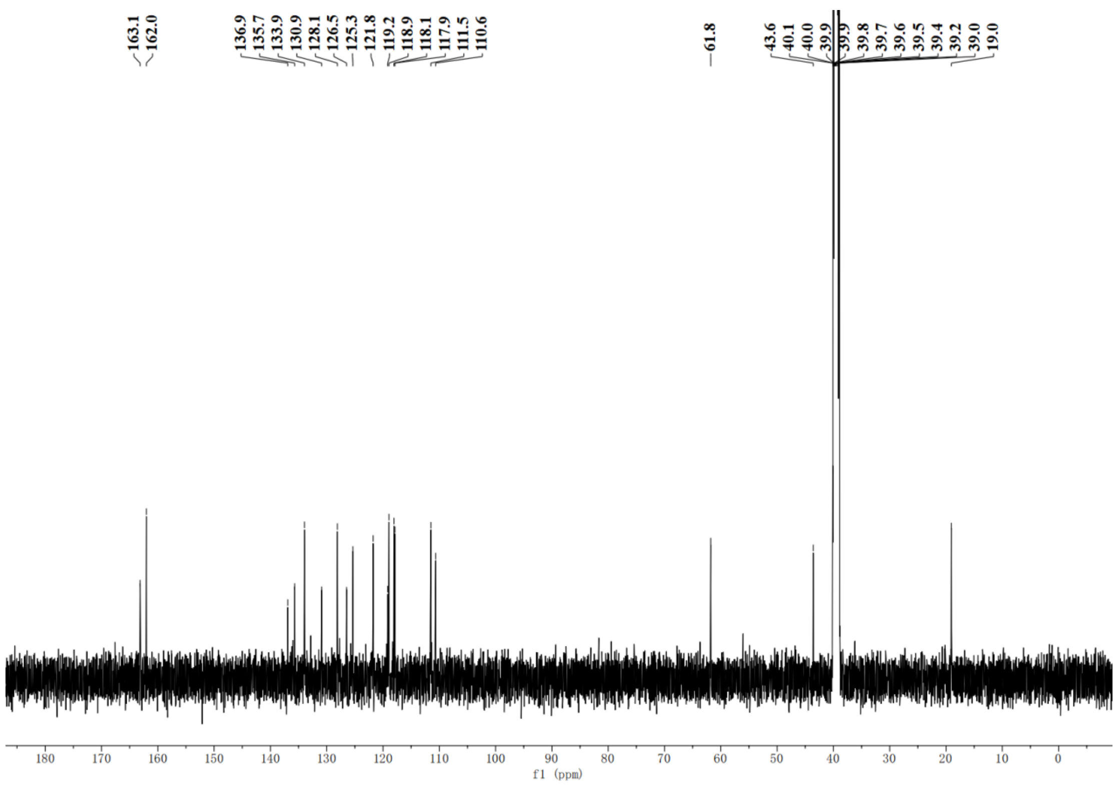

^{13}C-NMR spectrum of 14-formyldihydrorutaecarpine

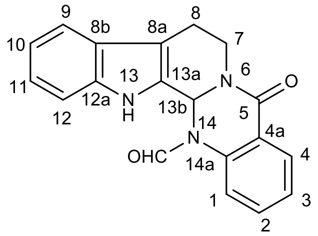

14-formyldihydrorutaecarpine

^{1}H NMR (400 MHz, DMSO-d_6) δ: 11.09 (1H, s, NH), 9.12 (1H, s, CHO), 7.89 (1H, d, J = 7.6 Hz, H-4), 7.64 (1H, ddd, J = 8.4, 8.4, 1.6 Hz, H-2), 7.60 (1H, dd, J = 8.0 Hz, H-9), 7.38 (1H, d, J = 8.0 Hz, H-12), 7.28 (1H, dd, J = 8.0, 8.0 Hz, H-11), 7.26 (1H, d, J = 8.0 Hz, H-1), 7.06 (1H, dd, J = 8.0, 7.2 Hz, H-10), 6.97 (1H, dd, J = 8.0, 7.2 Hz, H-3), 4.71 (1H, dd, J = 13.2, 6.0 Hz, H-7a), 3.62 (1H, ddd, J = 12.4, 12.4, 4.8 Hz, H-7b), 3.01 (1H, m, H-8a), 2.67 (1H, dd, J = 15.6, 4.0 Hz, H-8b); ^{13}C NMR (100 MHz, DMSO-d_6) δ: 163.1 (C-5), 162.0 (N-CHO), 136.9 (C-14a), 136.9 (C-12a), 135.7 (C-2), 133.9 (C-4), 132.9 (C-13a), 130.9 (C-3), 128.1 (C-4a), 125.3 (C-8b), 121.8 (C-11), 119.2 (C-10), 118.9 (C-9), 117.9 (C-1), 111.5 (C-8a), 110.6 (C-12), 61.8 (C-13b), 43.6 (C-7), 19.0 (C-8).
