# Supplementary material for: Target-Centric Multiplexed Screening of an Herbal Extract Identifies a Novel Dual A2A/A2B Receptor Antagonist for Cancer Immunotherapy
Source: ACS Cent Sci. 2026 Mar 13;12(3):358–74. doi: 10.1021/acscentsci.5c01843 (PMC13022719; doi:10.1021/acscentsci.5c01843)
Supplement: Supplementary file 6 [file oc5c01843_si_006.pdf]

Table S5. The inhibitory effect of ER-15 against other GPCRs evaluated by Tango assay.

GPCRs	Know agonists	ER-15 IC_{50} (nM)
ADORA3	NECA	>10000
GPR65	H^+	>10000
GPR81	Lactate	>10000
GPR91	Succinic acid	>10000
GPR183	7α,25-0HC	>10000
EP2	PGE2	>10000
EP4	PGE2	>10000
β1AR	Epinephrine	>10000
α1AR	Epinephrine	>10000
α1BR	Epinephrine	>10000
α2AR	Epinephrine	>10000
5HT2AR	5-HT	>10000
5HT2BR	5-HT	>10000
5HT2CR	5-HT	>10000
DRD4	Dopamine	>10000
CHRM4	Acetylcholine	>10000
HRH1	Histamine	>10000

Table S6. Potency and efficacy of ER-15 and ZM241385 antagonism related to Figure 4.

Potency and efficacy of ER-15 antagonism on $A_{2A}R$ WT and mutants.

$A_{2A}R$	WT	K153A	F168A	W246A	N253A
pIC_{50}	7.07 ± 0.11	6.26 ± 0.12	6.02 ± 0.15	6.35 ± 0.11	5.96 ± 0.12
E_{max}	93.04 ± 6.84	99.50 ± 8.45	107.51 ± 13.79	100.63 ± 7.87	113.54 ± 11.21

Potency and efficacy of ZM241385 antagonism on $A_{2A}R$ WT and mutants.

$A_{2A}R$	WT	K153A	F168A	W246A	N253A
pIC_{50}	7.95 ± 0.05	7.88 ± 0.06	7.07 ± 0.13	6.79 ± 0.05	ND
E_{max}	103.16 ± 5.41	99.80 ± 3.71	102.88 ± 9.32	99.98 ± 3.79	ND

Potency and efficacy of ER-15 antagonism on $A_{2B}R$ WT and mutants.

$A_{2B}R$	WT	C154A	C154K
pIC_{50}	6.31 ± 0.04	6.29 ± 0.11	6.34 ± 0.04
E_{max}	107.40 ± 10.15	100.1 ± 6.65	94.46 ± 7.70

ND, no antagonistic activity determined.

Table S7. Information of the patients.

Identifier	Gender	Age	Cancer types
3788	Male	91	Colorectal cancer
4420	Female	77	Colorectal cancer
8874	Female	53	Colorectal cancer
