# Supplementary material for: Target-Centric Multiplexed Screening of an Herbal Extract Identifies a Novel Dual A2A/A2B Receptor Antagonist for Cancer Immunotherapy
Source: ACS Cent Sci. 2026 Mar 13;12(3):358–74. doi: 10.1021/acscentsci.5c01843 (PMC13022719; doi:10.1021/acscentsci.5c01843)
Supplement: Supplementary file 7 [file oc5c01843_si_007.pdf]

oc-2025-01843c.R1

Name: Peer Review Information for "Target-centric multiplexed screening of a herbal extract identifies a novel dual A2A/A2B receptor antagonist for cancer immunotherapy"

First Round of Reviewer Comments

Reviewer: 1

Comments to the Author

Liu et al. present an innovative, multiplexed platform that integrates virtual screening, affinity selection–mass spectrometry (AS-MS), and metabolomics to discover novel immunotherapeutic natural products. Using this approach, they identify ER-15, a dual A2AR/A2BR antagonist from Evodia rutaecarpa, and demonstrate its immunomodulatory activity and synergy with anti-PD-1 therapy in murine models and patient-derived tumor organoids. The MS is well elaborated and the data is solid and supports the major observations, yet several issues need be addressed prior to publication.

1. While the study characterizes ER-15's antagonistic activity toward A_1, A_2A, and A_2B receptors, counter-screening against a broader GPCR panel would be highly valuable to confirm target specificity.

2. To demonstrate that ER-15 has no toxicity toward tumor cells and T cells, a more comprehensive characterization of MC38-OVA and OT-1 cells—such as cell cycle, and apoptosis—is required, going beyond the viability data shown in S8.

3. Given that adenosine levels are markedly elevated in the tumor microenvironment, it would be important to assess whether ER-15 retains its antagonistic activity toward the A_2A and A_2B receptors under increased adenosine (NECA) concentrations.

4. In Figure 6, it would be helpful to include immunofluorescence staining for PD-1 in the patient-derived tumor organoids sections to further corroborate the flow cytometry data.

Reviewer: 2

Comments to the Author

Shui and colleagues applied a target-centric screening approach to identify an active ingradient from herbal extracts that binds to adenosine 2A receptor (A2aR) and modulates its function in immunotherapy. The screening process includes affinity selection mass spectrometry that developed by the same group previously, virtual docking of the compounds from herbal extracts as well as fractionated active metabolite profiling. The resulting compound ER-15 was convincingly shown to bind A2aR and its homologue A2bR via biochemical and structural validation. More interestingly, the compound was able to module A2aR funnction and demonstrates a potential in augmenting immunotherapy. The study was elegantly and rigorously performed with a combination of interdisciplinary experimental and computational techniques and the manuscript was well-written with clarity. I support its publication with a few minor points addressed:

1. Figure 1, ZM241358 was shown as the A2aR agonists, however, it was discussed in the main text as atagonists. So this needs clarification.

2. The other compound CGS15943 was mentioned in Figure 1 legend, but it does not seem to be described in the maintext at all.

3. Figure 1A and Figure 3G, why are the three receptors analyzed with different controls compounds, ZM241358 vs CGS15943? If possible, they should be done with the same control.

4. Figure 1E is not very informative with the ending of a cartoon mass spectrometer. What is the actual analysis by MS should be presented in the figure.

5. ER-15 should be labeled in Figure 1G.

6. Figure 2G is also confusing. What are the actual numbers shown above each bar? And what are the black dots under the barplot?

Reviewer: 3

Comments to the Author

This manuscript by Liu et al. presents a comprehensive and technically sophisticated study that identifies ER-15, a novel natural product isolated from the traditional Chinese medicinal herb Evodia rutaecarpa, as a dual antagonist of adenosine receptors A2AR and A2BR. The authors employ a powerful, integrated discovery pipeline combining virtual screening, affinity selection–mass spectrometry (AS-MS), and metabolomics profiling. The work is rigorous, progressing seamlessly from in silico and in vitro identification to detailed mechanistic characterization—including binding mode analysis, mutagenesis, and structure–activity relationship (SAR) studies—and ultimately to robust functional validation in cellular, animal, and patient-derived organoid (PDO) models. The findings hold substantial significance for cancer immunotherapy, particularly in addressing resistance to anti–PD-1 therapy.

Major Comments

1. A2AR blockade is known to remodel multiple immune compartments within the TME beyond $CD8^+$ T cells. It would significantly strengthen this study to examine at least one of the following populations: regulatory T cells (Tregs), macrophages, or dendritic cells (DCs). Assessing potential reductions in Treg abundance, enhancement of DC antigen presentation, or polarization of macrophages toward an M1-like phenotype would provide valuable mechanistic insight into how ER-15 reprograms the TME.

2. Quantification of progenitor-exhausted (Tpex) and terminally exhausted (Ttex) $CD8^+$ T cell subsets in the tumor and tumor-draining lymph nodes would provide more convincing evidence for improved T cell function following ER-15 treatment. If these analyses are technically challenging, please include a rationale or discuss this limitation in the revised manuscript.

Minor Comments

1. While NMR data confirm the chemical structure of ER-15, it would be standard practice to include HPLC purity data (≥95%) for the isolated compound used in biological assays. This ensures that the reported activities can be confidently attributed to ER-15 itself.

2. The reported potency of ER-15 (IC_{50} = 87.7 nM for A2AR) is encouraging. However, a side-by-side comparison with the clinical dual A2AR/A2BR antagonist AB928—either experimentally or through reference to prior studies—would help contextualize ER-15’s pharmacological profile and potential for further development. The authors should discuss

whether ER-15 demonstrates comparable potency or efficacy, or if additional optimization is warranted.

Author's Response to Peer Review Comments:

> Reviewer(s)' Comments to Author:
> Reviewer: 1 >
> Recommendation: Publish in ACS Central Science after minor revisions noted. >
> Comments:
> Liu et al. present an innovative, multiplexed platform that integrates virtual screening, affinity selection–mass spectrometry (AS-MS), and metabolomics to discover novel immunotherapeutic natural products. Using this approach, they identify ER-15, a dual A2AR/A2BR antagonist from Evodia rutaecarpa, and demonstrate its immunomodulatory activity and synergy with anti-PD-1 therapy in murine models and patient-derived tumor organoids. The MS is well elaborated and the data is solid and supports the major observations, yet several issues need be addressed prior to publication.

1. While the study characterizes ER-15's antagonistic activity toward A_1, A_2A, and A_2B receptors, counter-screening against a broader GPCR panel would be highly valuable to confirm target specificity.

Response: We thank the reviewer for raising this important point regarding compound selectivity. To address this concern, we conducted a broad counterscreen of ER-15 against a panel of 17 additional class A GPCRs. As shown below, ER-15 had barely any antagonistic activity against these GPCRs (IC_{50} > 10 μM) in Tango assays which measured β-arrestin2 recruitment to receptors. These new results confirm that ER-15 is a selective antagonist for A2A/A2B receptors and have been incorporated into the revised manuscript (Table S5).

Table S5. The inhibitory effect of ER-15 against other GPCRs evaluated by Tango assays.

GPCRs	Reference agonists	ER-15 IC_{50} (nM)
ADORA3	NECA	>10000
GPR65	H^+	>10000
GPR81	Lactate	>10000
GPR91	Succinic acid	>10000

GPR183	7α,25-OHC	>10000
EP2	PGE2	>10000
EP4	PGE2	>10000
β1AR	Epinephrine	>10000
α1AR	Epinephrine	>10000
α1BR	Epinephrine	>10000
α2AR	Epinephrine	>10000
5HT2AR	5-HT	>10000
5HT2BR	5-HT	>10000
5HT2CR	5-HT	>10000
DRD4	Dopamine	>10000
CHRM4	Acetylcholine	>10000
HRH1	Histamine	>10000

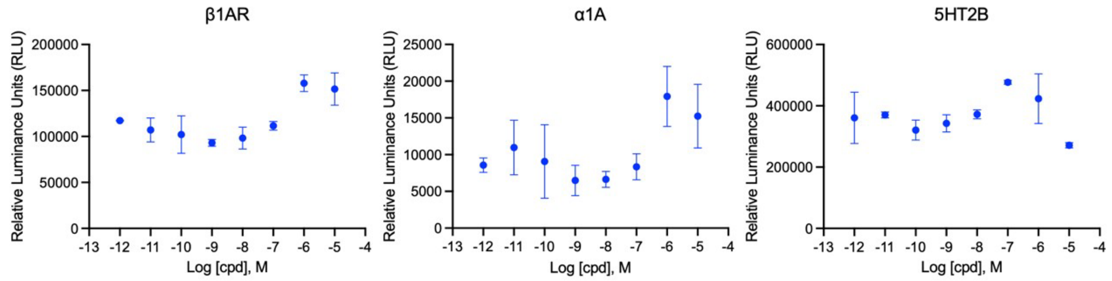

Figure R1. Representative dose-response curves of ER15 on several aminergic receptors measured by Tango assays. ER15 displayed no significant antagonist activity up to 10 μM.

2. To demonstrate that ER-15 has no toxicity toward tumor cells and T cells, a more comprehensive characterization of MC38-OVA and OT-1 cells—such as cell cycle, and apoptosis—is required, going beyond the viability data shown in S8.

Response: We thank this reviewer's excellent suggestion. We have now conducted additional assays to assess ER-15–associated cytotoxicity. As shown in Figure S9, ER-15 does not induce cell-cycle arrest or apoptosis in either MC38-OVA tumor cells or OT-I T cells. These results, now added to Supplementary Fig. 8, substantiate that ER-15 lacks direct cytotoxic effects and instead acts through an immunomodulatory mechanism.

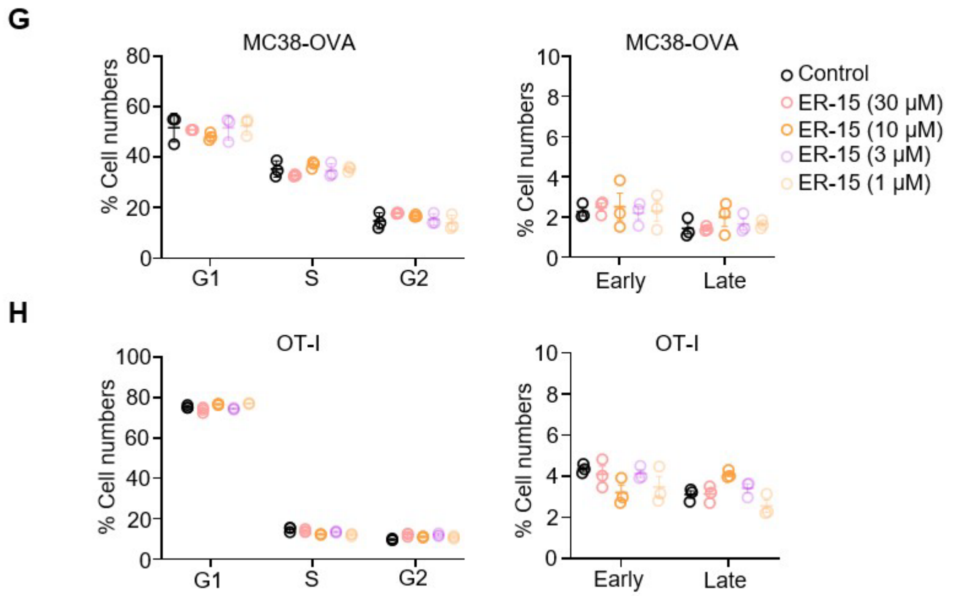

Figure S9. (**G-H**) Cell cycle and cell apoptosis analysis of MC38-OVA (**G**) and OT-I (**H**) cells upon ER-15 treatment (1, 3, 10, 30 μM, for 48 h).

3. Given that adenosine levels are markedly elevated in the tumor microenvironment, it would be important to assess whether ER-15 retains its antagonistic activity toward the A_2A and A_2B receptors under increased adenosine (NECA) concentrations.

Response: We thank this reviewer for this critical point. To address this concern, we have performed new cellular assays with increasing NECA concentrations. Our data show that raising the NECA concentration moderately reduced the potency of ER-15 compared to the original condition (2.5 nM for $A_{2A}R$, 300 nM for $A_{2B}R$). Specifically, for $A_{2A}R$, the IC_{50} shifted from 76.2 nM (at 2.5 nM NECA) to 569.8 nM (at 100 nM NECA). For $A_{2B}R$, the IC_{50} shifted from 499.7 nM (at 300 nM NECA) to 1110 nM (at 3000 nM NECA). This indicates that its antagonizing activity could be largely retained at an elevated adenosine level. The new data have been incorporated into the revised Figure S4.

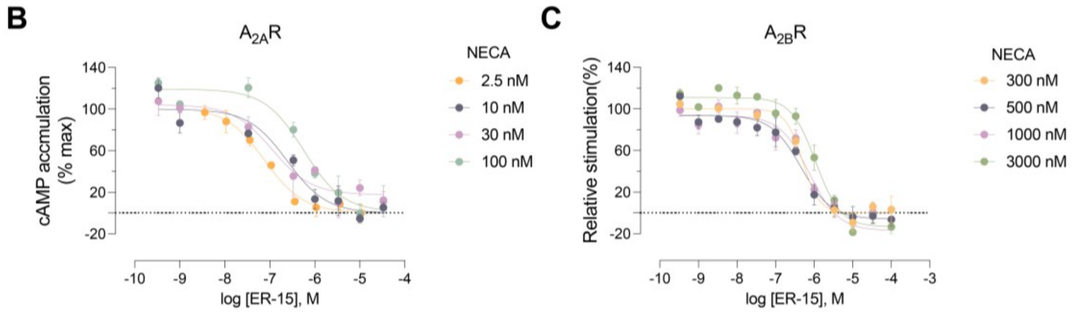

NECA conc	2.5 nM	10 nM	30 nM	100 nM
IC_{50} of ER-15 (nM)	76.2	246.0	113.8	569.8

NECA conc	300 nM	500 nM	1000 nM	3000 nM
IC_{50} of ER-15 (nM)	499.7	456.6	750.6	1110

Figure S4: (**B-C**) The dose−response curves of ER-15 against $A_{2A}R$ (**B**) and $A_{2B}R$ (**C**) under various levels of NECA concentration. Cells were pretreated with various levels of NECA concentration for 15 min. Data are shown as means ± SEM from three independent experiments. Mean IC_{50} values from experimental triplicate are shown in the tables.

4. In Figure 6, it would be helpful to include immunofluorescence staining for PD-1 in the patient-derived tumor organoids sections to further corroborate the flow cytometry data.

Response: We thank the reviewer for this insightful suggestion. Following the recommendation, we have now performed immunofluorescence staining for PD-1 on sections of the patient-derived tumor organoids analyzed in Figure 6. The immunofluorescence results are fully consistent with our flow cytometry findings, showing a clear and reproducible reduction in PD-1 expression upon ER-15 treatment. We have included the new results in the revised manuscript (Figure 6E).

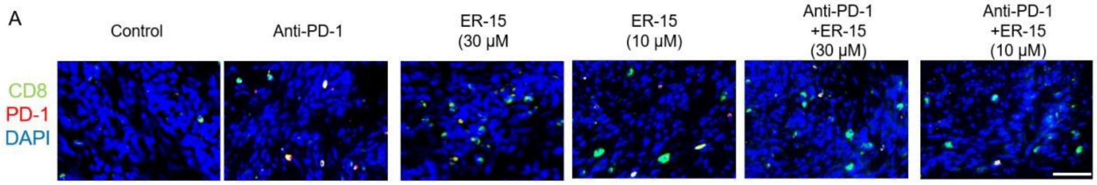

Figure 6. (**E**) Representative images of immunofluorescence analysis of PDOs stained for CD8/PD-1 following treatment with vehicle, ER-15 (30, 10 μM), antiPD-1 antibody (Pembrolizumab, 5 μg/mL), or their combination. Scale bar = 40 μm.

>
>
> Additional Questions:
> Quality of experimental data, technical rigor: Top 10%
>
> Significance to chemistry researchers in this and related fields: Top 10% >
> Broad interest to other researchers: Top 10%
>
> Novelty: Top 10%
>
> Is this research study suitable for media coverage or a First Reactions (a News & Views piece in the journal)?: Yes
>
>

> Reviewer: 2 >

> Recommendation: Publish in ACS Central Science after minor revisions noted. >

> Comments:

> Shui and colleagues applied a target-centric screening approach to identify an active ingradient from herbal extracts that binds to adenosine 2A receptor (A2aR) and modulates its function in immunotherapy. The screening process includes affinity selection mass spectrometry that developed by the same group previously, virtual docking of the compounds from herbal extracts as well as fractionated active metabolite profiling. The resulting compound ER-15 was convincingly shown to bind A2aR and its homologue A2bR via biochemical and structural validation. More interestingly, the compound was able to module A2aR funnction and demonstrates a potential in augmenting immunotherapy. The study was elegantly and rigorously performed with a combination of interdisciplinary experimental and computational techniques and the manuscript was well-written with clarity. I support its publication with a few minor points addressed:

>

> 1. Figure 1, ZM241358 was shown as the A2aR agonists, however, it was discussed in the main text as atagonists. So this needs clarification.

Response: We thank the reviewer for pointing out this error in our writing. We have corrected on Page 46: “ZM241358 is the reference antagonist for $A_{2A}R$ and $A_{2B}R$”

> 2. The other compound CGS15943 was mentioned in Figure 1 legend, but it does not seem to be described in the maintext at all.

Response: Because they are reference compounds, we added specific description to Figure 1 legend: “ZM241358 is the reference antagonist for $A_{2A}R$ and $A_{2B}R$; CGS15943 is the reference antagonist for A_1R.”.

> 3. Figure 1A and Figure 3G, why are the three receptors analyzed with different controls compounds, ZM241358 vs CGS15943? If possible, they should be done with the same control.

Response: We thank the reviewer for raising this question. While ZM241358 exhibits a low-nM potency at $A_{2A}R$, it showed a much weaker potency at A_1R (IC_{50} ~ 1 μM) in our assay. Since CGS15943 has a potency higher than ZM241358 for A_1R (IC_{50}= 71 nM) in the same system, it was selected as the reference for A_1R.

4. Figure 1E is not very informative with the ending of a cartoon mass spectrometer. What is the actual analysis by MS should be presented in the figure.

Response: We thank the reviewer for this helpful suggestion. We have revised Figure 1E by replacing the cartoon with a schematic of LC-MS data from affinity MS analysis.

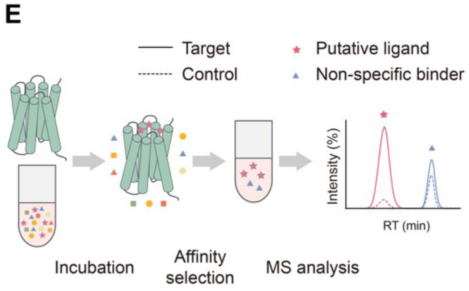

5. ER-15 should be labeled in Figure 1G.

Response: We have labeled ER-15 in Figure 1G.

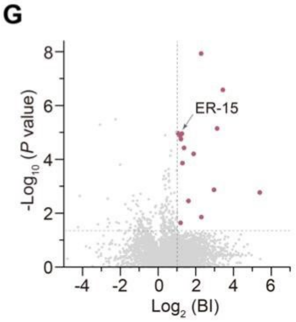

6. Figure 2G is also confusing. What are the actual numbers shown above each bar? And what are the black dots under the barplot?

Response: We have revised the legend as follows for a clearer description: (**G**) Total numbers of screening hits yielded by three different approaches (left three bars) and numbers of intersecting hits co-identified from two or three approaches (right four bars). Intersecting hits from three approaches yielded only one hit, ER-15, co-identified by all.

>

> Additional Questions:

> Quality of experimental data, technical rigor: Top 10%

>

> Significance to chemistry researchers in this and related fields: Top 10% >

> Broad interest to other researchers: Top 10%

>

> Novelty: Top 10%

>

> Is this research study suitable for media coverage or a First Reactions (a News & Views piece in the journal)?: Yes
>
>
> Reviewer: 3 >
> Recommendation: Publish in ACS Central Science after minor revisions noted. >
> Comments:
> This manuscript by Liu et al. presents a comprehensive and technically sophisticated study that identifies ER-15, a novel natural product isolated from the traditional Chinese medicinal herb Evodia rutaecarpa, as a dual antagonist of adenosine receptors A2AR and A2BR. The authors employ a powerful, integrated discovery pipeline combining virtual screening, affinity selection– mass spectrometry (AS-MS), and metabolomics profiling. The work is rigorous, progressing seamlessly from in silico and in vitro identification to detailed mechanistic characterization—including binding mode analysis, mutagenesis, and structure–activity relationship (SAR) studies—and ultimately to robust functional validation in cellular, animal, and patient-derived organoid (PDO) models. The findings hold substantial significance for cancer immunotherapy, particularly in addressing resistance to anti–PD-1 therapy.
>
> Major Comments
>1. A2AR blockade is known to remodel multiple immune compartments within the TME beyond $CD8^+$ T cells. It would significantly strengthen this study to examine at least one of the following populations: regulatory T cells (Tregs), macrophages, or dendritic cells (DCs). Assessing potential reductions in Treg abundance, enhancement of DC antigen presentation, or polarization of macrophages toward an M1-like phenotype would provide valuable mechanistic insight into how ER-15 reprograms the TME.

Response: We thank the reviewer for this very insightful comment. In line with the suggestion, we examined the impact of ER-15 on macrophage polarization within the TME. Specifically, we assessed M1-like macrophages, which support antitumor immunity by enhancing the cytotoxic activity of $CD8^+$ T cells and NK cells. Using immunofluorescence staining with established M1 markers, we found that ER-15 treatment markedly increased the content of M1-like macrophages compared with the vehicle group (revised Figure R2). These data indicate that ER-15 can remodel the tumor microenvironment beyond $CD8^+$ Tcell regulation. We agree with reviewer that a comprehensive characterization of additional immune compartments—such as Tregs and dendritic cells—will further refine this mechanistic understanding and represents an important direction for future work. Due to the scope of this study and page limit, this data will not be included in the revised manuscript.

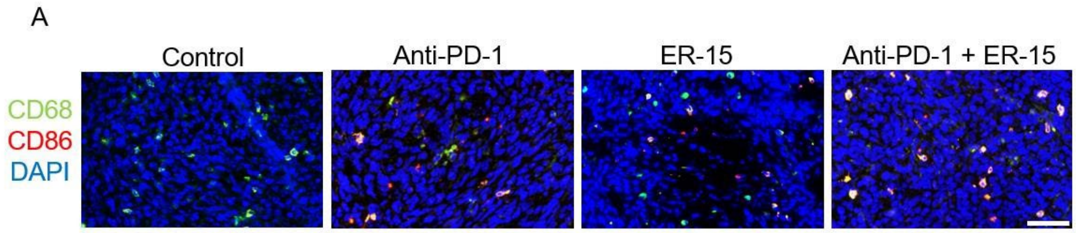

Figure R2: Representative images of immunofluorescence analysis of MC38 tumor sections stained for CD68/CD86 following treatment with control, antiPD-1 antibody, ER-15 or their combination as in Figure 5F. Scale bar, 50 μm.

2. Quantification of progenitor-exhausted (Tpex) and terminally exhausted (Ttex) $CD8^+$ T cell subsets in the tumor and tumor-draining lymph nodes would provide more convincing evidence for improved T cell function following ER-15 treatment. If these analyses are technically challenging, please include a rationale or discuss this limitation in the revised manuscript.

Response: We very much thank the reviewer for this important suggestion. To further delineate the impact of ER-15 on $CD8^+$ T-cell differentiation states, we examined progenitor-exhausted (Tpex) and terminally exhausted (Ttex) $CD8^+$ T-cell subsets in tumor tissues. Using immunofluorescence with established markers ($CD8^+TCF\text{-}1^+$ for Tpex and $CD8^+TIM\text{-}3^+$ for Ttex), we found that ER15 treatment significantly increased the proportion of Tpex cells while reducing the abundance of Ttex cells (Figure S10). These findings indicate that ER-15 not only alleviates $CD8^+$ T-cell exhaustion but also shifts the differentiation trajectory toward a more stem-like, therapeutically responsive state. The new data have been incorporated into the revised Figure S10.

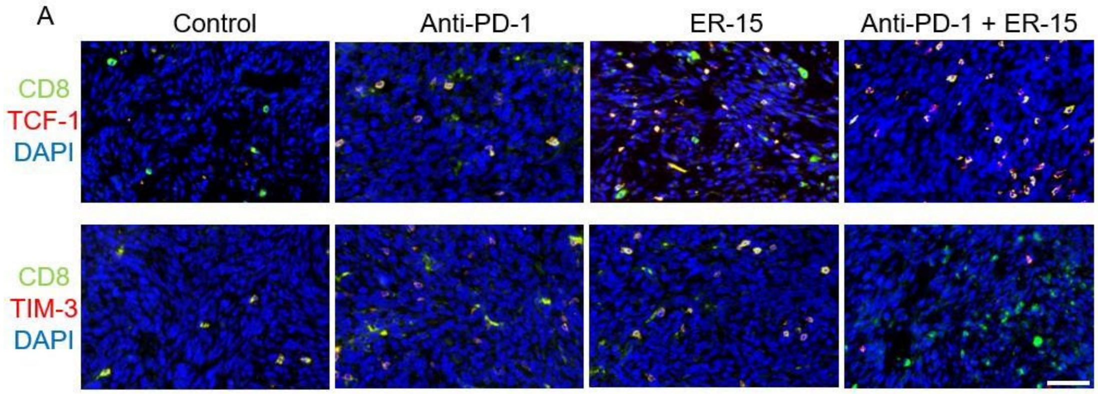

Figure S10. (**C**) Representative images of immunofluorescence analysis of MC38 tumor sections stained for progenitor-exhausted $CD8^+$ T-cells (Tpex, $CD8^+TCF\text{-}1^+$) and terminally exhausted $CD8^+$ T-cells (Ttex, $CD8^+TIM\text{-}3^+$) following treatment with vehicle, anti-PD-1 antibody, ER-15 or their combination as in Figure 5F. Scale bar, 50 μm.

> Minor Comments

1. While NMR data confirm the chemical structure of ER-15, it would be standard practice to include HPLC purity data (≥95%) for the isolated compound used in biological assays. This ensures that the reported activities can be confidently attributed to ER-15 itself.

Response: We thank this reviewer for this helpful suggestion and have included the HPLC purity data in Note S1.

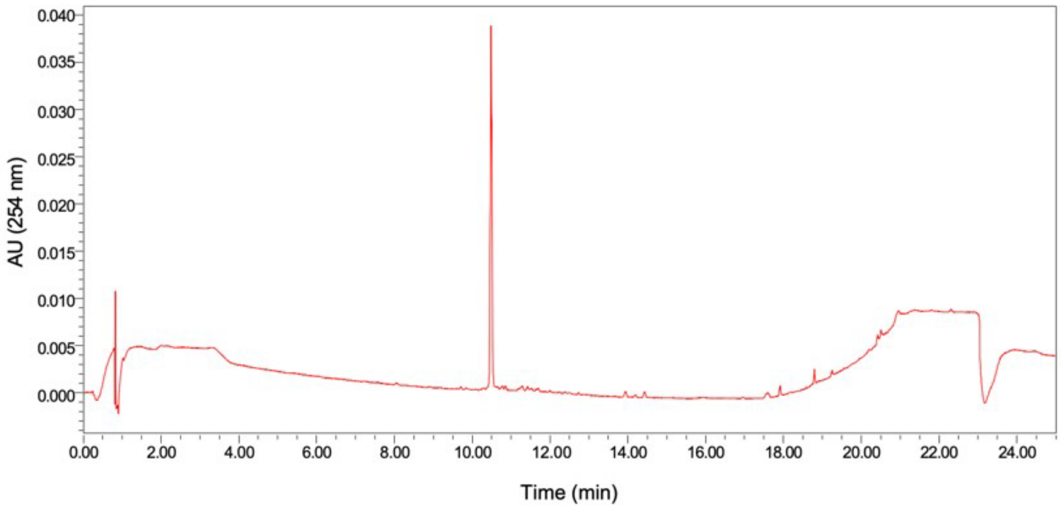

HPLC chromatogram of purified ER-15

2. The reported potency of ER-15 (IC_{50} = 87.7 nM for A2AR) is encouraging. However, a side-by-side comparison with the clinical dual A2AR/A2BR antagonist AB928—either experimentally or through reference to prior studies—would help contextualize ER-15's pharmacological profile and potential for further development. The authors should discuss whether ER-15 demonstrates comparable potency or efficacy, or if additional optimization is warranted.

Response: Following the reviewer's great suggestion, we have added a short discussion to the revised manuscript:

"Although ER-15 showed *in vitro* antagonizing activity 8 or 80-fold weaker than the synthetic clinical compound AB928 (IC_{50} = 11.2 nM for $A_{2A}R$, 6.4 nM for $A_{2B}R$), we were able to validate the *in vivo* anti-tumor efficacy of ER-15, particularly synergistic activity with anti-PD-1 therapy, in both animal and patient-derived tumor models." We do agree with the reviewer that the activity of ER-15 has large room for improvement.

oc-2025-01843c.R2

Name: Peer Review Information for "Target-centric multiplexed screening of a herbal extract identifies a novel dual A2A/A2B receptor antagonist for cancer immunotherapy"

Second Round of Reviewer Comments

Reviewer: 1

Comments to the Author

The authors have addressed my concerns.

Reviewer: 3

Comments to the Author

The revised manuscript has addressed all major concerns, it is ready to be published.

Reviewer: 2

Comments to the Author

The authors did a good job in revising the manuscript and I support its publication

Author's Response to Peer Review Comments:

Dear Editor,

We have submitted the revision with the authorship change form and designed cover art for your consideration.

Thank you very much for your great effort in handling our manuscript. We really appreciated all reviewer's comments and feedback.

-Sincerely yours,

Wenqing Shui
